# Observation of localization reversal and harmonic generation in nonlinear non-Hermitian skin effect

**DOI:** 10.1038/s41467-026-74610-y

**Published:** 2026-06-26

**Authors:** Junyao Wu, Rui-Chang Shen, Li Zhang, Fujia Chen, Bingbing Wang, Hongsheng Chen, Yihao Yang, Haoran Xue

**Affiliations:** 1 State Key Laboratory of Extreme Photonics and Instrumentation, ZJU-Hangzhou Global Scientific and Technological Innovation Center, Zhejiang University, Hangzhou, 310027 China; 2 The Electromagnetics Academy at Zhejiang University, College of Information Science and Electronic Engineering, Zhejiang University, Hangzhou, 310027 China; 3 Zhejiang Key Lab. of Intelligent Electromagnetic Control and Advanced Electronic Integration, Jinhua Institute of Zhejiang University, Zhejiang University, Jinhua, 321099 China; 4 Department of Physics, The Chinese University of Hong Kong, Hong Kong SAR, China; 5 State Key Laboratory of Quantum Information Technologies and Materials, The Chinese University of Hong Kong, Hong Kong SAR, China

**Keywords:** Nonlinear phenomena, Topological matter, Photonic devices

## Abstract

The interplay between band topology and material nonlinearity gives rise to a variety of novel phenomena, such as topological solitons and nonlinearity-induced topological phase transitions. However, most previous studies fall within the Hermitian regime, leaving the impact of nonlinearity on non-Hermitian topology seldom explored. Here, we investigate the effects of nonlinearity on the non-Hermitian skin effect, a well-known non-Hermitian phenomenon induced by the point-gap topology unique to non-Hermitian systems. We discover a nonlinearity-induced point-gap topological phase transition accompanied by a reversal of the skin mode localization. This phenomenon is experimentally demonstrated in a nonlinear microwave metamaterial, where electromagnetic waves are localized around one end of the sample under a low-intensity pump, whereas at a high-intensity pump, the waves are localized around the other end. We also observe third harmonic generation signal induced by the skin modes, whose spatial distribution consistently shows the localization reversal. Furthermore, we extend our setup to build a nonlinear non-Hermitian Su-Schrieffer-Heeger model with both nonlinearity-induced point-gap and line-gap phase transitions. Our results open a new route towards nonlinear topological physics in non-Hermitian systems and are promising for reconfigurable topological wave manipulation and frequency conversion.

## Abstract (web summary)

Nonlinearity strongly affects topological phases, yet its role in non-Hermitian topology remains little explored. Here, authors show that nonlinearity drives a point-gap topological transition, reversing the non-Hermitian skin effect and controlling harmonic generation in a microwave metamaterial.

## Introduction

Topological photonics, a field where the concept of band topology developed in condensed matter is utilized to design photonic structures, offers a promising route towards robust manipulation of electromagnetic waves^1,2^. Since the realization of photonic Chern insulators at microwave frequencies^3,4^, various topological phases have been successfully implemented in photonics, including the quantum spin Hall phase^5–7^, valley Hall phase^8–10^, Floquet topological phase^11–13^, among others. A key factor behind these rapid developments is that the source-free Maxwell equation can be cast into a linear eigenproblem, whose eigenmodes can be used to define topology using standard topological band theory. However, there are two noticeable exceptions, i.e., non-Hermitian and nonlinear systems, where conventional topological band theory is not applicable. The study of these systems has led to a new revolution in topological photonics, together with various novel topological phenomena^14–16^.

Non-Hermiticity, resulting from nonconservation of energy, is ubiquitous in photonic systems due to material loss and/or gain. When it is taken into consideration, the topological classification and even the notion of topology will be significantly altered, leading to many topological phenomena without Hermitian counterparts^17–19^. One prominent example is the non-Hermitian skin effect (NHSE), in which an extensive number of eigenmodes are localized at an open boundary due to the nontrivial point-gap topology under the periodic boundary condition (PBC)^20–25^. The NHSE has been realized in photonics using different platforms^26–31^, with potential applications in lasing and sensing^32–37^.

Meanwhile, nonlinearity has also been shown to have a significant impact on topological photonic systems. While proper definitions of band topology in nonlinear systems are still an ongoing topic, several interesting phenomena from the interplay between nonlinearity and band topology have already been discovered, such as modifications of topological modes from nonlinearity (e.g., to form solitons)^38–44^, frequency conversion using topological modes^45–49^, and topological phase transitions driven by nonlinearity^50–54^. In particular, nonlinearity-induced topological phase transitions, which go beyond the straightforward nonlinearity-topological mode interaction picture, suggest a deep and subtle interplay between band topology and nonlinearity and offer an appealing method to control topological propagation by tuning input power instead of system parameters.

While previous studies have revealed fruitful topological physics from non-Hermitian or nonlinear topological systems separately, little is known about systems that are simultaneously non-Hermitian and nonlinear^55,56^. As non-Hermitian systems can host point-gap topological phases that are absent in Hermitian ones, new physics is expected to emerge when they are enriched by nonlinearity. Recently, considerable efforts have been put into studying the impact of nonlinearity on the NHSE, showing that the skin modes can be significantly tuned by nonlinearity^57–65^. Particularly, previous studies in low-frequency systems have shown that nonlinearity can lead to the opening of a point gap, and thus, the emergence of skin modes^64,65^. However, whether nonlinearity can induce a topological phase transition between different nontrivial point-gap phases remains unexplored.

**Fig. 1:**
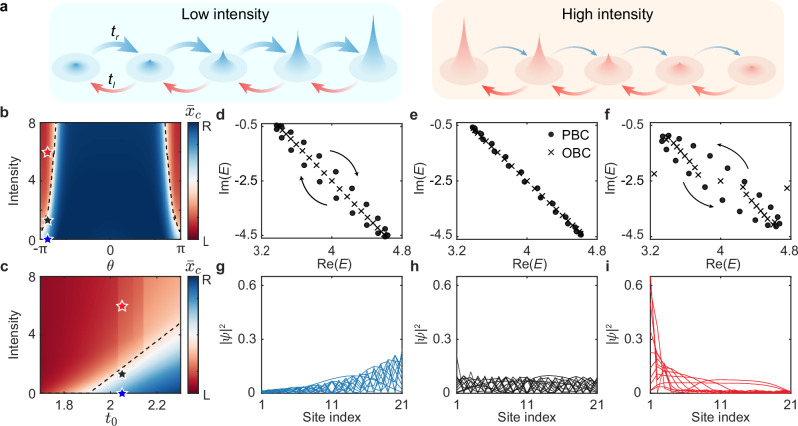
Nonlinear Hatano-Nelson model. **a** Schematic of the nonlinear Hatano-Nelson model and nonlinearity-induced reversal of skin localization. Here, the *t*_*r*(*l*)_ represents the rightward (leftward) coupling, which is nonlinear (linear). **b**, **c** Plot of the average position of all OBC eigenvectors as functions of intensity *I* and *θ* (and *t*_0_), with a fixed *t*_0_ = 2.05 (*θ* = − 0.9*π*). The black dashed lines denote the point-gap closing points obtained from PBC spectra. **d**–**f** Plots of eigenvalues on the complex plane under PBC (dots) and OBC (crosses) for the three intensity values highlighted by the blue (**d**), black (**e**), and red (**f**) star markers in (**b**, **c**). **g**–**i** The corresponding OBC eigenvectors for (**d**–**f**). In all calculations, we take *f*_0_ = − 2.5*i*, *t*_*l*_ = 1, *t*_*∞*_ = 0.2 and *t*_*c*_ = 1.

In this work, we theoretically and experimentally discover that nonlinearity can induce a topological phase transition between two nontrivial point-gap topological phases in an electromagnetic non-Hermitian metamaterial at gigahertz frequencies. This phase transition leads to a localization reversal phenomenon: the modes originally localize at the right boundary are relocated to the left boundary. Specifically, we introduce a nonlinear Hatano-Nelson model with saturable nonreciprocal couplings, which is known as the minimal model for the NHSE in the linear limit^66^. In our nonlinear case, we find that the direction of the NHSE, or equivalently, the point-gap topology, can be solely controlled by nonlinearity. Consequently, the field localization from the NHSE can be reversed by simply increasing the input power (see Fig. 1a). This phenomenon is experimentally observed in a microwave metamaterial through port-resolved voltage measurements and near-field scanning of the electric field distribution. We also demonstrate for the first time the harmonic generation of the skin modes at high pump strength, and successfully capture the localized fields at the harmonic frequency that resemble the skin localization. Moreover, we construct a nonlinear non-Hermitian Su-Schrieffer-Heeger (SSH) model^67^ and discover that the point-gap and line-gap topology can be controlled by nonlinearity simultaneously. These findings extend the study of nonlinear topological phenomena to the point-gap regime.

## Results

### Nonlinear Hatano-Nelson model

Consider a classical nonlinear Hatano-Nelson model as depicted in Fig. 1a, which generically describes an array of *N* coupled photonic resonators with linear reciprocal couplings and nonlinear nonreciprocal couplings. The system is governed by the coupled-mode equations:

1 $$i\frac{d{\Psi }_{n}}{dt}={f}_{0}{\Psi }_{n}+{t}_{r,n}{\Psi }_{n-1}+{t}_{l,n}{\Psi }_{n+1},$$

where *n* is the site index, *Ψ*_*n*_ is the complex field amplitude at the *n*-th site, *f*_0_ is the resonant frequency, and *t*_*l*(*r*),*n*_ denotes the leftward (rightward) nearest-neighbor coupling. Here, the leftward coupling *t*_*l*,*n*_ = *κ*_1,*n*_ while the rightward coupling ${t}_{r,n}={\kappa }_{1,n}+{\widetilde{\kappa }}_{2,n}$, where *κ*_1,*n*_ is a conventional linear and reciprocal coupling (e.g., an evanescent coupling) and ${\widetilde{\kappa }}_{2,n}$ is a specially engineered nonlinear coupling that only exists in the rightward hopping process. A concrete realization of ${\widetilde{\kappa }}_{2,n}$ will be introduced later. Since *κ*_1,*n*_ is uniform across all sites, we write it as *κ*_1_ hereafter. In the theoretical model, we set *κ*_1_ = 1 as the normalization unit, while in the experimental sections *κ*_1_ takes its physical value retrieved from measurements. The nonlinear nonreciprocal coupling, which depends on the field intensity, is given by

2 $${\widetilde{\kappa }}_{2,n}({I}_{n})=\left(\frac{{t}_{0}-{t}_{\infty }}{1+{I}_{n}/{t}_{c}}+{t}_{\infty }\right){e}^{i\theta }.$$

Here, *I*_*n*_ = ∣*Ψ*_*n*_∣^2^ is the field intensity at the *n*-th site, *t*_0_ and *t*_*∞*_ correspond to the coupling strengths at zero and infinite intensity, respectively, *t*_c_ governs how coupling varies with intensity, and *θ* is the phase of the coupling. Note that, due to the unidirectional nature of this coupling, its strength is only affected by the field intensity on the *n*-th site but not the (*n* + 1)-th site. This modeling is also consistent with our experimental implementation, as we will illustrate later. In the linear Hatano-Nelson model, the localization direction of the skin modes is simply determined by the relative strength between the leftward and rightward couplings. Hence, in our case, as only one of them is nonlinear, it is intuitive to expect an intensity-driven reversal of the NHSE.

To investigate the NHSE in this model, we search for time-harmonic solutions of the form *Ψ*_*n*_(*t*) = *ψ*_*n*_*e*^−*iEt*^ to Eq. (1). This leads to the nonlinear eigenproblem *Hψ* = *Eψ*, where *E* is the eigenvalue, $\psi={({\psi }_{1},{\psi }_{2},\ldots,{\psi }_{N})}^{T}$ is the eigenvector, and *H* is the Hamiltonian containing the on-site and coupling terms (see Methods for more numerical details). To account for the nonuniform coupling distribution induced by intensity fluctuation in space, we adopt a chain with *N* = 21 sites and connect the first and last sites to form a closed loop to compute the PBC spectrum. To characterize the collective localization behavior of the eigenmodes, we calculate the average position of all modes under open boundary conditions (OBCs), defined as

3 $${\bar{x}}_{c}=\left\langle {\sum }_{j=1}^{N}| {\psi }_{j}{| }^{2}\frac{(j-(N+1)/2)}{(N-1)/2}\right\rangle,$$

where *ψ*_*j*_ is the amplitude of the eigenmode at site *j*, 〈 ⋅ 〉 denotes the average over all eigenmodes, and ${\bar{x}}_{c}\in [-1,1]$. A positive (negative) value of ${\bar{x}}_{c}$ indicates that the mode is localized at the right (left) end of the lattice.

Figure 1b shows the calculated ${\bar{x}}_{c}$ as functions of the coupling angle *θ* and the total intensity *I* = ∑_*n*_∣*ψ*_*n*_∣^2^. The topological phase transition points of the NHSE are captured by ${\bar{x}}_{c}=0$, separating modes localized at opposite ends. Meanwhile, the transition can also be judged from the closing of the point-gap under PBC, as denoted by the black dashed lines, which coincide well with the ${\bar{x}}_{c}=0$ boundaries of the OBC modes. Notably, the intensity-driven reversal of the NHSE occurs near *θ* = ± *π*, where the interference between reciprocal and nonreciprocal couplings is significant. Whereas at *θ* = 0, *κ*_1_ and ${\widetilde{\kappa }}_{2}$ have the same sign regardless of the mode intensity, resulting in an absence of topological phase transition. In Fig. 1c, we plot the distribution of ${\bar{x}}_{c}$ in the *I* − *t*_0_ plane for a fixed *θ* = − 0.9*π*, where a reversal of the NHSE is also found. A threshold, given by $| {\kappa }_{1}+{\widetilde{\kappa }}_{2,n}({I}_{n}\to 0)| > {\kappa }_{1}$, is required, such that rightward couplings are dominant at zero intensity. In addition, the high-intensity coupling needs to satisfy $| {\kappa }_{1}+{\widetilde{\kappa }}_{2,n}({I}_{n}\to \infty )| < {\kappa }_{1}$ to make the reversal happen.

To see in detail the reversal process, we pick up three points in the phase diagrams (see the markers in Fig. 1b, c) and plot the corresponding PBC and OBC eigenvalues and the OBC eigenmodes. As shown in Fig. 1d-i, the modes are localized at opposite ends before and after the phase transition. A complementary winding-number characterization of the PBC Hamiltonian is provided in Suppl. Note 1. Furthermore, the eigenvalues under OBC fall inside the loops of the PBC spectra, consistent with the feature of the NHSE. All these numerical results suggest a point-gap topological phase transition induced by nonlinearity.

### Implementation of nonlinear nonreciprocal coupling at microwave frequencies

**Fig. 2:**
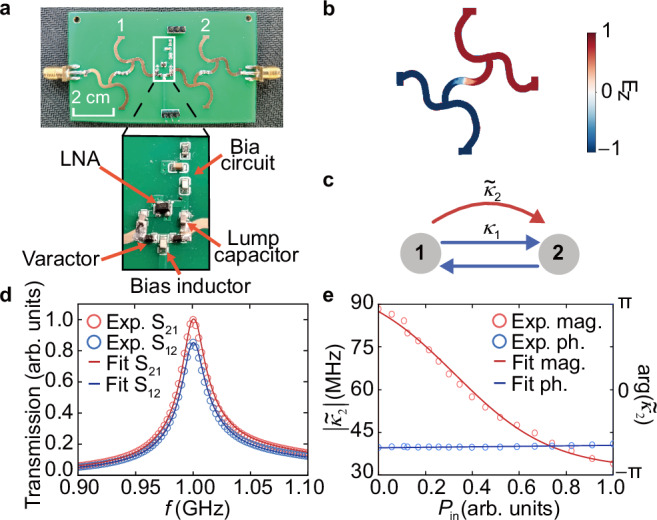
Implementation of microwave nonlinear nonreciprocal couplings. **a** Photograph of a sample consisting of two coupled microwave resonators (labeled 1 and 2). The lower panel shows the details of the coupling region. **b** Simulated eigenmode profile of a single microwave resonator. **c** Simplified tight-binding model for the setup in (**a**). **d** Experimentally measured (circles) and numerically calculated (curves) response spectra for the setup in (**a**). The transmission is normalized to maximum linear response of *S*_21_. **e** Experimentally measured (circles) and numerically calculated (curves) magnitude and phase as a function of input power *P*_in_ at 1.056 GHz. The source and probe are at resonators 1 and 2, respectively.

To realize the theoretical model, we first propose a design to realize the nonlinear non-Hermitian coupling using two coupled microwave resonators. As illustrated in Fig. 2a, the system consists of two identical resonators (labeled 1 and 2) made of copper strips on a FR4 substrate, with each supporting a dipolar mode at 1.2 GHz^68^ (see Fig. 2b for the mode profile and Suppl. Note 4 for more sample details). The coupling between them is enabled in two ways. Firstly, the two resonators are connected by series varactors, which provide a reciprocal coupling *κ*_1_ (see the lower panel of Fig. 2a). This coupling can be adjusted by tuning the DC bias voltage (see Suppl. Note 3). Secondly, a unidirectional coupling, denoted by $\widetilde{{\kappa }}_{2}$, is introduced by an RF amplifier circuit equipped with a low-noise amplifier chip (LNA) and a corresponding bias structure, as depicted in the lower panel of Fig. 2a. The microwave signal from resonator 1 traverses the lumped capacitor and is detected by the LNA. Then it is unidirectionally amplified and coupled to resonator 2 via the other lumped capacitor. The amplitude gain and phase shift introduced by the LNA are captured by the complex coefficient $\widetilde{{\kappa }}_{2}$. At low input power, the LNA operates in the linear regime, and $\widetilde{{\kappa }}_{2}$ remains effectively constant. As the input power increases, nonlinear gain compression occurs, where the fundamental amplification is reduced, and waveform distortion emerges, leading to a power-dependent complex coupling coefficient. In this way, a nonlinear nonreciprocal coupling is realized in the present setup. In practice, the experimentally accessible parameter window of the coupling is limited by component tolerances, amplifier nonlinear operating range, and measurement noise, which may induce resonator detuning and introduce additional effective gain or loss. However, by choosing high-quality LNA and conducting careful calibration, the desired parameter values can be reached.

Under the dipolar mode basis, the Hamiltonian of this system can be written as (see Fig. 2c and Supplementary Note 3 for the circuit-based derivation)

4 $${H}_{{{{\rm{eff}}}}}=\left(\begin{array}{rcl}{f}_{0}-i{\gamma }_{0}-i{\gamma }_{1} & & {\kappa }_{1}\\ {\kappa }_{1}+{\widetilde{\kappa }}_{2} & & {f}_{0}-i{\gamma }_{0}-i{\gamma }_{1}\end{array}\right)$$

where *f*_0_ is the resonance frequency of the individual resonators, *κ*_1_ and ${\widetilde{\kappa }}_{2}$ are the reciprocal and nonreciprocal couplings. *γ*_0_ arises from the intrinsic loss, including the conductor loss, dielectric loss, and lumped resistor loss, and *γ*_1_ is the external decay rate. Next, we measure the transmission spectra ∣*S*_21_∣ and ∣*S*_12_∣ under different input powers to validate the modeling and retrieve the model parameters. The measured spectra at input power *P*_in_ = − 25 dBm are shown in Fig. 2d, which are not identical when the excitation and source positions are exchanged, indicating the presence of nonreciprocity. Using a Green’s function method, we can establish the relationship between the transmission spectra and the tight-binding coefficients, allowing us to extract model parameters from experimental transmission data (see Suppl. Note 2). Figure 2e plots the retrieved magnitude and phase of ${\widetilde{\kappa }}_{2}$ against the input power, which fit well with the nonlinear coupling model (i.e., Eq. (2)). Note that the phase of ${\widetilde{\kappa }}_{2}$ is carefully engineered to ensure the reversal of the NHSE (see Fig. 1b).

### Reversal of the NHSE driven by nonlinearity

**Fig. 3:**
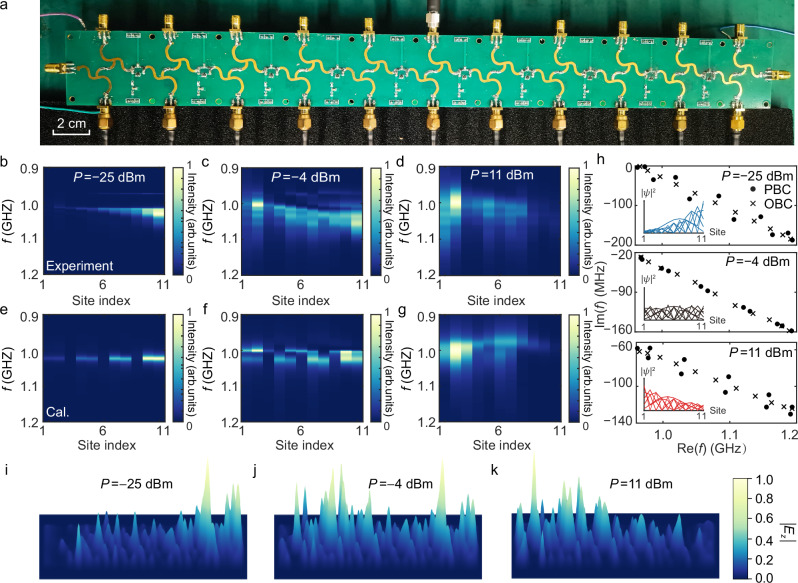
Observation of nonlinearity-induced reversal of the NHSE. **a** Photograph of a sample composed of 11 identical resonators. The source is connected to the lower ports, whereas the probe is affixed to the upper ports. **b**–**d** Measured field distributions at *P*_in_ = − 25 dBm (**b**), *P*_in_ = − 4 dBm (**c**), and *P*_in_ = 11 dBm (**d**), each normalized to its maximum response at the corresponding input power. **e**–**g** Simulated field distributions at *P*_in_ = − 25 dBm (**e**), *P*_in_ = − 4 dBm (**f**), and *P*_in_ = 11 dBm (**g**), likewise normalized at each power level. **h** Calculated PBC (circles) and OBC (crosses) eigenfrequencies and eigenmodes (insets) for the three cases in (**b**–**d**). **i**–**k** Experimentally measured electric field distributions at 1.013 GHz (∣*E*_*z*_∣ component) with *P*_in_ = − 25 dBm (**i**), *P*_in_ = − 4 dBm (**j**), and *P*_in_ = 11 dBm (**k**), each normalized to the maximum field amplitude at the corresponding power.

We expand our system to a one-dimensional crystal consisting of 11 microwave resonators, as shown in Fig. 3a, with the coupling configuration between two neighboring resonators the same as the one in Fig. 2a. Note that we use fewer resonators (*N* = 11) to facilitate the experimental implementation, and the localization reversal phenomenon persists under the size change of the system. To excite the skin modes, a power splitter uniformly divides a source with tunable power into 11 distinct segments, which are then simultaneously introduced into the resonators through the lower SMA ports (see Methods for more experimental details). Such an excitation averages the initial energy of the system and mitigates the effect of input ports. We use two methods to obtain the field distribution in the sample, as described below.

We first measure the transmission spectrum at each resonator through the other SMA ports as the data for each lattice site. With a low input power (*P*_in_ = −25 dBm), the rightward coupling takes the lead, and a skin localization around the right end of the sample is experimentally observed (Fig. 3b), which is also consistent with the numerical eigenmodes and field distribution obtained using retrieved tight-binding parameters (Fig. 3e, h). With increased power, the NHSE gradually becomes weaker, and the field becomes almost delocalized at around *P*_in_ = −4 dBm, as can be seen in Fig. 3c, f, h. Furthermore, when the input power exceeds this transition value (*P*_in_ = 11 dBm), a reversal of the NHSE, highlighted by a field localization around the left end of the sample, occurs as predicted (Fig. 3d, g, h).

Additionally, we map out the electric field distribution (out-of-plane component) near the surface of the sample to more precisely capture the NHSE. The experimental results at 1.013 GHz for the three input power values are shown in Fig. 3i–k. Despite now being two-dimensional field plots, the key characteristics (i.e., the localization behaviors) are the same as those revealed by the one-dimensional field plots given in Fig. 3b–d using port measurements. Specifically, from low input power to high input power, we see a reversal of the NHSE from the right to the left end of the sample. The associated localization behavior is further quantified in Suppl. Note 7 through the localization length.

The nonlinear coupling strength sets how sensitively localization responds to increasing input power, while its phase determines whether this change preserves or reverses the original localization direction. A nonlinear contribution with the same sign as the intrinsic nonreciprocal bias maintains the localization direction, whereas an opposite sign enables reversal. Larger systems or higher-dimensional geometries can further enhance this behavior by allowing stronger energy accumulation under nonreciprocal transport. Its robustness against disorder is further examined in Suppl. Note 6.

### Harmonic generation produced by skin modes

**Fig. 4:**
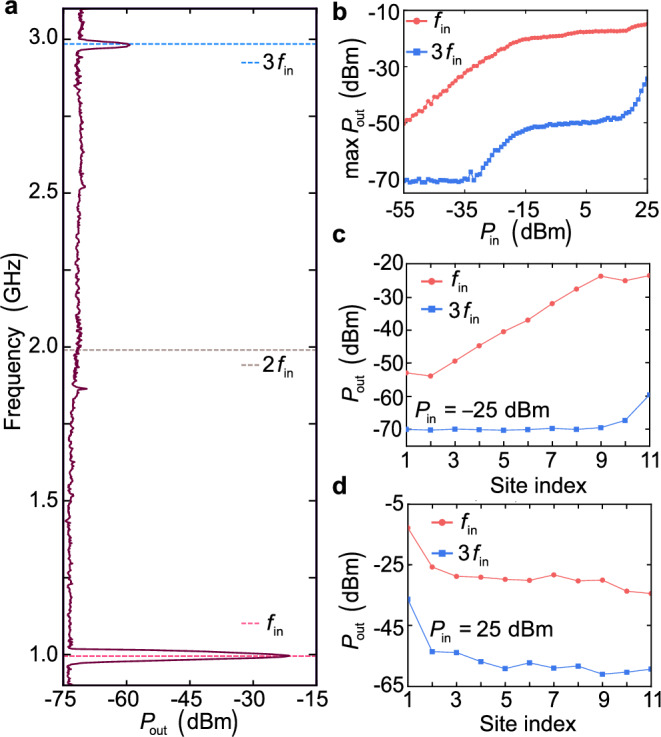
Observation of harmonic signals produced by the skin modes. **a** Measured output spectrum at site 11 under input power *P*_in_ = − 25 dBm, driven at *f*_in_ = 0.994 GHz (slightly shifted from 1.013 GHz due to the change in the measurement setup). **b** Maximum output power among all sites at fundamental (*f*_in_) and third (3*f*_in_) harmonics as a function of increasing input power *P*_in_. **c**, **d** Field localization behavior of fundamental and third harmonics, measured at *P*_in_ = − 25 dBm and *P*_in_ = 25 dBm, respectively.

Next, we investigate harmonic generation of the skin modes, which has not been discussed in previous literature. For this purpose, we supply a sinusoidal continuous-wave input voltage signal with tunable frequency *f*_in_ and power *P*_in_ at site 1, then collect the spectrum of all sites. The single-port excitation scheme is adopted intentionally because if multiple ports are driven simultaneously, each excitation channel may introduce weak parasitic higher-harmonic components from the source and feeding network, thereby leading to unwanted harmonic contributions in the measured signal. A typical measurement result at port 11 is shown in Fig. 4a, for *P*_in_ = − 25 dBm and input frequency *f*_in_ ranging from 0.95 to 1.05 GHz (i.e., at the frequencies of the skin modes). A clear peak at the third harmonic frequency (3*f*_in_) is observed, as indicated by the blue dashed line. The harmonic signal stems from the saturable nonlinearity of the LNAs, and even harmonics are suppressed due to the symmetry of the RF chip (see Suppl. Note 5). The third harmonic component appears only after the power of the fundamental mode exceeds a threshold level, and then grows concurrently with the fundamental as the input power increases, as demonstrated in Fig. 4b. More interestingly, the field distributions at the third harmonic frequency inherit the localization property of the skin modes at the fundamental frequency. This is shown in Fig. 4c, d, where the localization direction of the third harmonic signals reverses following the nonlinearity-induced topological phase transition at the fundamental frequency when the input power increases from −25 dBm to 25 dBm. These observations indicate that we can control the spatial distribution of the harmonic fields using a tunable NHSE at fundamental frequencies.

### Nonlinear nonreciprocal SSH model

**Fig. 5:**
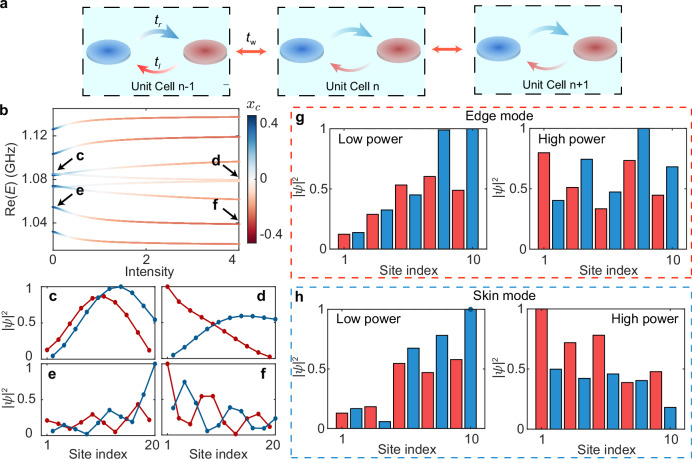
Nonlinear nonreciprocal SSH model. **a** Schematic of the nonlinear SSH model. **b** Evolution of the real part of eigenvalues with respect to the total intensity *I*. **c**–**f** The evolution of the eigenmodes, with their corresponding eigenvalues and intensities labeled in (**b**). Red and blue curves correspond to the odd and even sites, respectively. **g**, **h** Experimentally measured field distributions obtained by exciting the system with an input field whose frequency and phase correspond to the edge mode (**g**), and the skin mode (**h**). The parameters used in the calculations are *t*_*l*_ = *t*_*w*_ = 66 MHz, *t*_0_ = 98.9 MHz, *t*_*∞*_ = 32 MHz, *θ* = − 2.13, and *t*_*c*_ = 0.022.

Finally, we extend our discussion to a nonlinear nonreciprocal SSH, whose linear version exhibits a series of novel phenomena from the interplay between point-gap and line-gap topologies, such as the breakdown of bulk-boundary correspondence and morphing of topological edge modes^20,69^. Specifically, the point-gap topological phase transition manifests as the reversal of the NHSE, while the line-gap phase transition governs the emergence of topological edge states. As shown in Fig. 5a, a rightward nonlinear coupling *t*_*r*_ (following Eq. (2)) is introduced to the intracell coupling. All other couplings remain linear. The eigenvalues and eigenmodes as functions of total intensity are plotted in Fig. 5b–f for a finite chain under OBC. With initial parameter conditions ∣*t*_*r*_∣ ⋅ ∣*t*_*l*_∣ > ∣*t*_*w*_∣^2^ and ∣*t*_*r*_∣ > ∣*t*_*l*_∣, the system is topologically trivial at low intensities, and the skin modes are localized around the right end (Fig. 5c, e). As the total intensity increases, the real parts of the eigenvalues of two modes gradually approach each other, corresponding to a line-gap phase transition. However, due to inevitable mode coupling in finite-length chains, these two modes are not exactly degenerate^50^. Meanwhile, a point-gap phase transition also occurs, as can be seen from the blue-to-red color change in Fig. 5b and the eigenmodes plots in Fig. 5e, f.

To further distinguish between edge modes and skin modes, we label the odd and even lattice sites with different colors. When the coupling strengths satisfy $| \ln(| {t}_{r}| /| {t}_{l}| )| < 2$, the edge modes exhibit opposite localization directions on odd and even sites, whereas the skin modes do not possess this property^70^. In our model, the nonlinear coupling strength ensures that this condition holds for all sites under arbitrary system intensities. As shown in Fig. 5d, f, at high intensities, the edge modes exhibit opposite localization directions on odd and even sites, while the skin modes localize in the same direction. This confirms the emergence of edge modes. In the experiment, we clearly observe that, as the input power increased, the field distribution on the odd and even sites at the frequency corresponding to the edge mode changes from same-side localization to opposite-side localization (Fig. 5g), accompanied by a reversal of the NHSE (Fig. 5h). This indicates the simultaneous realization of both point-gap and line-gap phase transitions in the nonlinear SSH model.

## Discussion

In summary, we have proposed a nonlinearity-induced topological phase transition of the unique non-Hermitian point-gap topology and experimentally observed the reversal of the NHSE driven by input power and the harmonic generation linked to the NHSE. On the fundamental level, our results demonstrate that nonlinearity can drive point-gap topological phase transitions in non-Hermitian systems, enabling the reversal of the NHSE through input power rather than structural modification. The harmonic signals generated by the skin modes further inherit this reversal, revealing an intrinsic connection between nonlinear frequency conversion and non-Hermitian topology. Practically, our scheme to realize the nonreciprocal coupling, which is much more compact and controllable compared to previous designs that require feedback controls^63,65^, is promising for reconfigurable and nonreciprocal manipulation of electromagnetic waves. Meanwhile, the successful generation of harmonic signals may open a new route for frequency conversion and generation using novel non-Hermitian mechanisms.

The key element in our system, i.e., amplifier-based nonreciprocal coupling combined with saturable nonlinearity, is not restricted to this specific implementation and is in principle applicable to other active wave platforms. Moreover, the current four-arm H-shaped resonator design naturally extends to two-dimensional networks, as each arm offers an independent coupling channel, facilitating planar connectivity^68,71^. Hence, it would be highly desirable to extend our system to two dimensions, where novel physics emerging from the interplay among nonlinearity, non-Hermiticity, and band topology can be further explored.

## Methods

### Nonlinear eigenproblem

We employ a self-consistent method implemented in MATLAB to solve the nonlinear eigen equation *H*(*ψ*)*ψ* = *Eψ* to obtain the eigenvalues and eigenmodes shown in Figs. 1, 5. For a one-dimensional chain with *N* sites, the eigen problem involves 2*N* + 2 unknown real variables to be solved, including the real and imaginary parts of each component of the wavefunction (i.e., Re(*ψ*_*n*_) and Im(*ψ*_*n*_)), and the real and imaginary parts of the eigenvalue (i.e., Re(*E*) and Im(*E*)). Yet there are only 2*N* + 1 equations, including 2*N* equations from the eigen equation and the intensity condition *I* = ∑_*n*_∣*ψ*_*n*_∣^2^. To solve this issue, consider a modification to the eigen problem, *H*(*ψ*)*e*^*iϕ*^*ψ* = *Ee*^*iϕ*^*ψ*, which states that when *ψ* is a solution, *e*^*iϕ*^*ψ* is also a solution. Thus, we can also find a proper *ϕ* to make the first component of the wavefunction be a real number (i.e., Im(*ψ*_1_) = 0), which reduces the unknown variables to 2*N* + 1. In our calculation, we start with a small intensity value *I* = 0.02 and use the linear eigenvalues and eigenmodes as initial guesses to input into the solver. Then, the intensity is gradually increased (*δI* = 0.02), with the initial guess for each step being the solutions from the previous step to ensure accuracy and computational efficiency.

### Sample details

The microwave resonators in this work are fabricated with printed circuit board (PCB) technology. Both sides of the microstrip board are coated with 35 *μ*m-thick copper layers, and the dielectric substrate is composed of 0.8 mm-thick FR4 material with a dielectric constant of 4.6 and a loss tangent of 0.02. The resonator is H-shaped^68^, where each section has the same length (2 cm), width (0.1 cm), and characteristic impedance (about 64 Ω). The unloaded resonance frequency (1.2 GHz) is decreased by coupling capacitors. A resistor is patched in the middle of the resonator for system stability. The LNA chip (MGA-53543) is designed with a bias circuit, while back-to-back varactors (SMV2201) are blocked with an isolated inductor (see Suppl. Note 4).

### Measurement setup

Measurements of fundamental frequencies are performed using a two-port vector network analyzer (VNA) (AV3672E) with tunable input power. In the measurement of the S-parameters (i.e., Fig. 2), the source and detector are directly connected to the two SMA ports of the resonators via RF flexible coax cables. In the port measurement of the lattice sample (i.e., Fig. 3b–d), the source signal is divided into 11 parts with identical magnitude by power dividers before input into the 11 resonators. Then the detector collects the transmission spectra one by one at the other port of each resonator. The measured frequency range is from 0.9 GHz to 1.3 GHz, with a total of 401 frequency points measured across this bandwidth. In the near-field measurement (i.e., Fig. 3i–k), the detector probe is 1 mm above the sample, and the scanning setup involves a 6-point grid in the *x* direction and a 71-point grid in the *y* direction, with a spacing of 5.6 mm between each point.

Measurements of harmonics (Fig. 4) are performed using a signal generator (SSG5060X-V) with tunable output power and frequency and a signal analyzer (N9020A). A continuous-wave source connects to resonator 1, generating a sinusoidal signal with frequencies from 0.95 GHz to 1.05 GHz in 2 MHz steps, and varying power from −55 dBm to 25 dBm in 1 dBm steps. The output spectrum is recorded using a signal analyzer over the frequency range of 0.9–3.1 GHz with a frequency resolution of 2 MHz. To avoid overloading and maintain precise power measurements, we employ a 15 dB attenuator in front of the analyzer’s input port.

## Supplementary information

Supplementary Information

Peer Review File

## Data Availability

The raw data used to generate the plots are available at Zenodo: 10.5281/zenodo.20559726^72^. Other data supporting this study’s findings are available from the corresponding authors upon request.

## Code availability

The codes used to generate the plots are available in the same Zenodo record: 10.5281/zenodo.20559726^72^. Other codes supporting this study’s findings are available from the corresponding authors upon request.
