# Supplementary material for: Observation of localization reversal and harmonic generation in nonlinear non-Hermitian skin effect
Source: Nat Commun. 2026 Jun 26;17:8028. doi: 10.1038/s41467-026-74610-y (PMC13454480; doi:10.1038/s41467-026-74610-y)
Supplement: Supplementary file 1 [file 41467_2026_74610_MOESM1_ESM.pdf]

Supplementary Information for
Observation of localization reversal and harmonic generation in nonlinear non-Hermitian skin effect

Junyao Wu,[1,2,3,4,*] Rui-Chang Shen,[5,*] Li Zhang,[1,2,3,4] Fujia Chen,[1,2,3,4] Bingbing Wang,[5] Hongsheng Chen,[1,2,3,4] Yihao Yang,[1,2,3,4,†] and Haoran Xue[5,6,‡]

[1]*Interdisciplinary Center for Quantum Information, State Key Laboratory of Modern Optical Instrumentation, Zhejiang University, Hangzhou 310027, China*

[2]*International Joint Innovation Center, The Electromagnetics Academy at Zhejiang University, Zhejiang University, Haining 314400, China*

[3]*Key Lab. of Advanced Micro/Nano Electronic Devices & Smart Systems of Zhejiang, Jinhua Institute of Zhejiang University, Zhejiang University, Jinhua 321099, China*

[4]*Shaoxing Institute of Zhejiang University, Zhejiang University, Shaoxing 312000, China*

[5]*Department of Physics, The Chinese University of Hong Kong, Shatin, Hong Kong SAR, China*

[6]*State Key Laboratory of Quantum Information Technologies and Materials, The Chinese University of Hong Kong, Shatin, Hong Kong SAR, China*

CONTENTS

Supplementary Note 1: Winding number for the nonlinear Hatano-Nelson model 3

Supplementary Note 2: Derivation of the transmission coefficients 3

Supplementary Note 3: Derivation of the coupled-mode equation 6

Supplementary Note 4: Experimental details of resonator components 8

Supplementary Note 5: Harmonics from Nonlinearity 9

Supplementary Note 6: Robustness of the localization reversal 12

Supplementary Note 7: Characterization of the skin depth 13

Supplementary Note 8: Nonlinear nonreciprocal SSH model 15

Supplementary References 17

SUPPLEMENTARY NOTE 1: WINDING NUMBER FOR THE NONLINEAR HATANO-NELSON MODEL

In this section, we introduce a topological index (i.e., the winding number) to characterize the point-gap topological phase transition and the localization reversal in the nonlinear Hatano-Nelson model. To this end, we introduce a magnetic flux Φ in the PBC Hamiltonian via multiplying the rightward and leftward coupling terms by the phase factors $e^{-i\frac{\Phi}{N}}$ and $e^{i\frac{\Phi}{N}}$, respectively, where N is the number of lattice sites [1]. This allows us to define the winding number of the system as:

$$w \equiv \int_0^{2\pi} \frac{d\Phi}{2\pi i} \partial_\Phi \ln \det \left(H(\Phi) - E_B \right), \tag{S1}$$

where $H(\Phi)$ is the Hamiltonian obtained by threading the magnetic flux Φ into the PBC Hamiltonian whose coupling strengths are determined by the intensity distribution of a given PBC eigenmode via Eq. (2) in the main text. E_B is chosen inside the point gap, and the winding number is invariant to its specific value. In our calculations, we take $E_B = 4 - 2.5\text{i}$. Here, the intensity-dependent coupling terms in the PBC Hamiltonian are obtained according to the intensity distribution of a certain PBC eigenmode. We note that the eigenmodes with the same total intensity yield the same winding number. As shown in Supplementary Fig. 1, the winding number of the PBC Hamiltonian undergoes a transition from -1 to 1 at an intensity around 1.82, corresponding to a change in the NHSE from right-sided to left-sided localization. This result is consistent with the findings presented in Fig. 1 in the main text.

SUPPLEMENTARY NOTE 2: DERIVATION OF THE TRANSMISSION COEFFICIENTS

In this section, we present the formulas for the transmission coefficients and explain the process of retrieving the linear and nonlinear system parameters. The frequency-domain Green's function of the two-resonator model in Fig. 2 of the main text can be written as [2, 3]:

$$G(f) = \left[fI - H_{\text{eff}} \right]^{-1}, \tag{S2}$$

where f denotes the driving frequency, I is the identity matrix, and H_{eff} is the Hamiltonian given in Eq. (4) of the main text. Consider two input/output channels connected to the

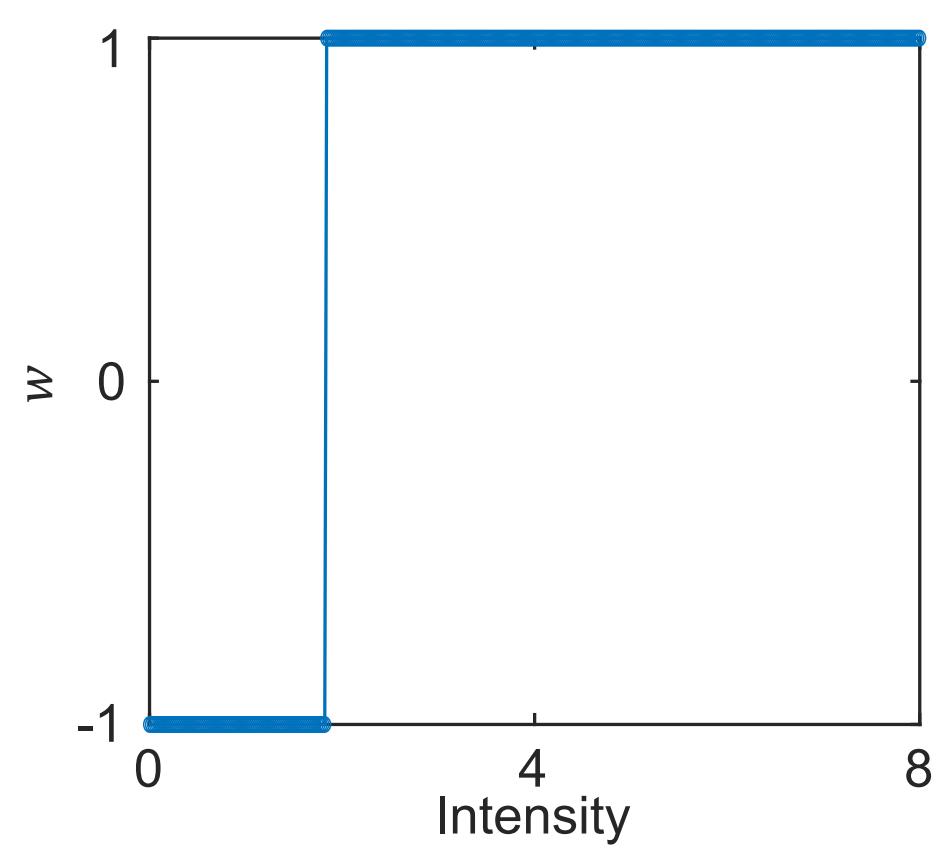

Supplementary Fig. 1 PBC winding number of the nonlinear Hatano-Nelson model. Plot of the winding number w of the PBC Hamiltonian as a function of total intensity.

two SMA ports of the system as shown in Fig. 2a in the main text. Under the Mahaux-Weidenmüller formula [4], we can obtain the transmission coefficients as

$$S(f) = I - W^{\dagger} G(f) W, \tag{S3}$$

where W is the channel–mode coupling matrix. Assuming symmetric external coupling rate γ_1 and that port 1 (port 2) couples only to resonator 1 (resonator 2), we take

$$W = \sqrt{2\gamma_1} \begin{pmatrix} 1 & 0 \\ 0 & 1 \end{pmatrix}, \tag{S4}$$

which leads to scattering coefficients upon substituting the matrix elements of $G(f)$ [5]:

$$S_{21} = -2\gamma_1 G_{21} = -\frac{2\gamma_1 \left(\kappa_1 + \tilde{\kappa}_2\right)}{\left(f - f_0 + i(\gamma_1 + \gamma_0)\right)^2 - \left(\kappa_1 + \tilde{\kappa}_2\right)\kappa_1}, \tag{S5}$$

$$S_{12} = -2\gamma_1 G_{12} = -\frac{2\gamma_1 \kappa_1}{\left(f - f_0 + i(\gamma_1 + \gamma_0)\right)^2 - \left(\kappa_1 + \tilde{\kappa}_2\right)\kappa_1}. \tag{S6}$$

Without the unidirectional coupling (i.e., $\tilde{\kappa}_2 = 0$), the transmission coefficients are reciprocal,

$$S_{21} = S_{12} = -\frac{2\gamma_1 \kappa_1}{\left(f - f_0 + i(\gamma_1 + \gamma_0)\right)^2 - {\kappa_1}^2}. \tag{S7}$$

We use Eq. (S7) to fit the measured transmission spectra. The retrieved system parameters are: the resonance frequency $f_0 = 1056\,\text{MHz}$, the reciprocal coupling $\kappa_1 = 66.2\,\text{MHz}$, the intrinsic loss $\gamma_0 = 42.3\,\text{MHz}$ and the coupling strength $\gamma_1 = 11\,\text{MHz}$.

Parameter	15 V	20 V	25 V
γ_1	16.67	16.59	17.10
γ_0	14.54	13.45	12.99
κ_1	46.00	36.66	13.99
f_0	1099.16	1106.77	1127.97

Supplementary Table 1 Retrieved parameters under different bias voltages.

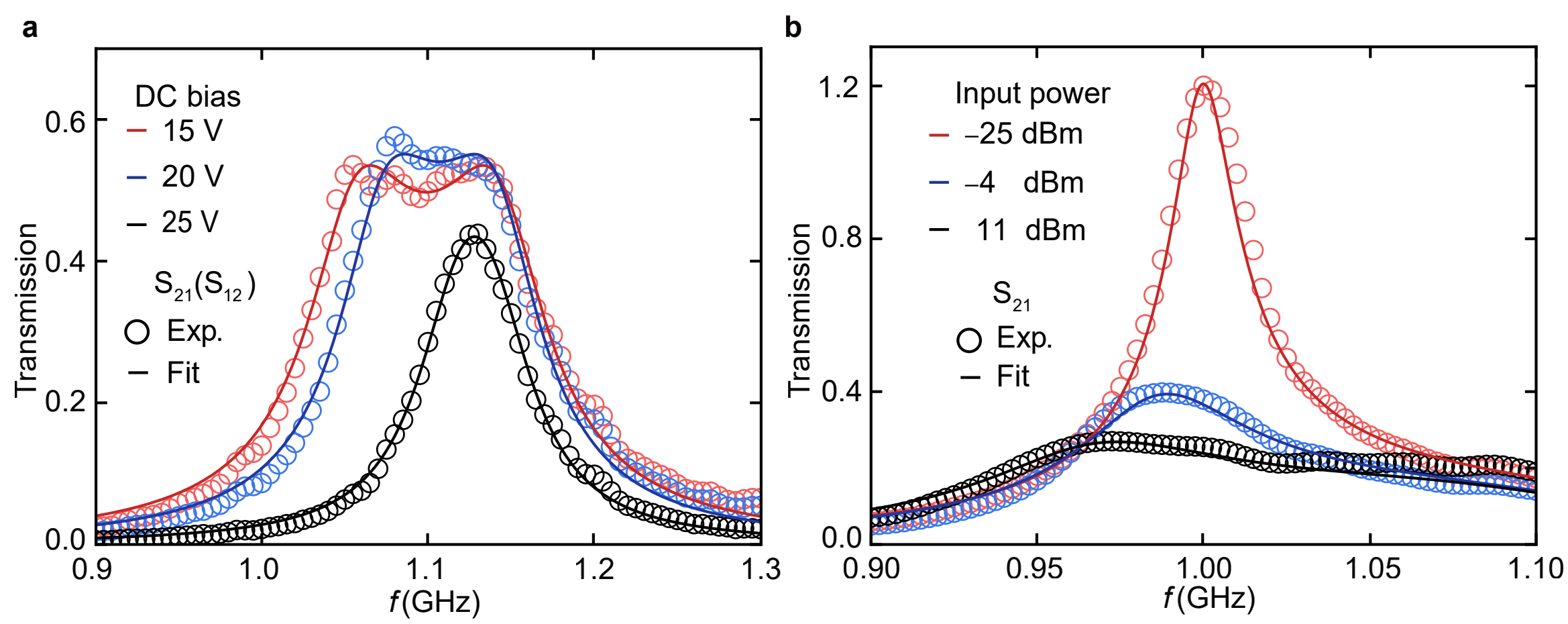

Supplementary Fig. 2 Regulation of reciprocal and nonreciprocal couplings. a, Experimentally measured (circles) and numerically fitted (solid lines) transmission spectra when $\tilde{\kappa}_2 = 0$ with different DC biases. b, Experimentally measured (circles) and numerically fitted (solid lines) transmission spectra when $\tilde{\kappa}_2 \neq 0$ with different input powers.

It is worth noting that, in our design, the capacitance of the varactors can be tuned by the DC bias. Therefore, the reciprocal coupling κ_1 can be well-controlled. Supplementary Fig. 2a shows the measured transmissions under different DC biases when the amplifier is disabled (i.e., $\tilde{\kappa}_2 = 0$). We can see that the splitting between the peaks in the spectra increases as the DC bias decreases, suggesting an increased coupling strength. Furthermore, other parameters are only slightly affected by the DC bias, as given in Supplementary Table 1. In our experiments, we set the DC bias to be 12 V, which corresponds to $\kappa_1 = 66.2$ MHz, to obtain a clear phenomenon.

Next, we turn on the amplifier and measure the transmission spectra under different input powers to obtain the nonlinear nonreciprocal coupling coefficient $\tilde{\kappa}_2$ from Eq. (S5) and

Eq. (S6). Several examples of measured spectra are shown in Supplementary Fig. 2b. For each input power, we can retrieve a corresponding $\tilde{\kappa}_2$. The complex number $\tilde{\kappa}_2$ is decomposed into its magnitude and phase as $\tilde{\kappa}_2 = Ae^{i\theta}$. Finally, we can fit the nonlinear model,

$$A(P) = \frac{a-b}{1+P/c} + b, \tag{S8}$$

$$\theta(P) = dP + e, \tag{S9}$$

where A and θ represent the magnitude and phase respectively, and P is the input power. The retrieved nonlinear parameters are $a = 98.94, b = 32.02, c = 0.022, d = 0.09, e = -2.13$. The setup features strong non-Hermiticity, characterized by a maximum eigen energy imaginary-to-real ratio of 8.77 %. We further demonstrate the tunability of the nonreciprocal strength by adjusting the reciprocal coupling κ_1 via the DC bias. At a bias of 25 V, κ_1 is reduced to 13.99 MHz, significantly amplifying the transport asymmetry. Consequently, the asymmetry factor $|S_{21}/S_{12}|$ reaches a maximum of 5.85, realizing robust and controllable directional transmission.

SUPPLEMENTARY NOTE 3: DERIVATION OF THE COUPLED-MODE EQUATION

In this section, we derive the Hamiltonian for the two-resonator system (i.e., Eq. (4) in the main text) using Kirchhoff's equations. The equivalent circuit model for the two-resonator system is depicted in Supplementary Fig. 3a, where resonators 1 and 2 are simplified as RLC elements [6, 7]. The source and detector of the vector network analyzer with 50 Ω internal resistance are labeled as R_1 and R_2 (assumed to be equal, $R_1 = R_2 = R_o$). Assuming a time dependence of $e^{-i2\pi ft}$, the Kirchhoff current equations are given by

$$\begin{aligned} I_{L,A} + \frac{V_A}{R_A} - i2\pi f C_A V_A - i2\pi f C_C (V_A - V_B) + \frac{V_A}{\frac{1}{-i2\pi f C_O} + R_1} &= 0, \\ I_{L,B} + \frac{V_B}{R_B} - i2\pi f C_B V_B - i2\pi f C_C (V_B - V_A) + Y_{21} V_A + \frac{V_B}{\frac{1}{-i2\pi f C_O} + R_2} &= 0. \end{aligned} \tag{S10}$$

where $I_{L,X} = \frac{V_X}{-i2\pi f L_X}$ with $X = A, B$ are currents flowing through the inductors. Considering the fact that the two resonators are identical, we have $R_A = R_B = R$, $L_A = L_B = L$, $C_A = C_B = C$, and $C_{eq} = C_O + C_C + C$. In the analysis of the admittance matrix, the self-admittance components Y_{11} and Y_{22}, as well as the reverse transmission admittance Y_{12}, are

sufficiently small to be neglected. Consequently, only the forward transmission admittance Y_{21} is taken into consideration. Eq. (S10) can be reformulated in a matrix form as

$$\begin{pmatrix} i2\pi\frac{f_0^2-f^2}{f}+\frac{1}{RC_{eq}}+\frac{4\pi^2 R_o f^2 C_O^2}{C_{eq}} & i\frac{2\pi f C_C}{C_{eq}} \\ i\frac{2\pi f C_C}{C_{eq}}+\frac{Y_{21}}{C_{eq}} & i2\pi\frac{f_0^2-f^2}{f}+\frac{1}{RC_{eq}}+\frac{4\pi^2 R_o f^2 C_O^2}{C_{eq}} \end{pmatrix}\begin{pmatrix} V_A \\ V_B \end{pmatrix}=0. \tag{S11}$$

Considering the working frequency near the linear resonance frequency $f_0=\frac{1}{2\pi\sqrt{LC_{eq}}}$, where $|f_0-f|\ll f$, we can simplify the equation as [8]

$$\begin{pmatrix} f_0-f-i\frac{\pi R_o f_0^2 C_O^2}{C_{eq}}-\frac{i}{4\pi RC_{eq}} & \frac{f_0 C_C}{2C_{eq}} \\ \frac{f_0 C_C}{2C_{eq}}-i\frac{Y_{21}}{4\pi C_{eq}} & f_0-f-i\frac{\pi R_o f_0^2 C_O^2}{C_{eq}}-\frac{i}{4\pi RC_{eq}} \end{pmatrix}\begin{pmatrix} V_A \\ V_B \end{pmatrix}=0. \tag{S12}$$

By taking $f_0=\frac{1}{2\pi\sqrt{LC_{eq}}}$, $\gamma_1=\frac{\pi R_o f_0^2 C_O^2}{C_{eq}}$, $\gamma_0=\frac{1}{4\pi RC_{eq}}$, $\kappa_1=\frac{f_0 C_C}{2C_{eq}}$, and $\tilde{\kappa}_2=-i\,\frac{Y_{21}}{4\pi C_{eq}}$, Eq. (S12) becomes

$$\begin{pmatrix} f_0-f-i(\gamma_1+\gamma_0) & \kappa_1 \\ \kappa_1+\tilde{\kappa}_2 & f_0-f-i(\gamma_1+\gamma_0) \end{pmatrix}\begin{pmatrix} V_A \\ V_B \end{pmatrix}=0. \tag{S13}$$

To explicitly formulate this system in the standard eigenvalue problem, we rearrange the equation as

$$[H_{\text{eff}}-fI]\begin{pmatrix} V_A \\ V_B \end{pmatrix}=0, \tag{S14}$$

where the Hamiltonian is defined as

$$H_{\text{eff}}=\begin{pmatrix} f_0-i(\gamma_0+\gamma_1) & \kappa_1 \\ \kappa_1+\tilde{\kappa}_2 & f_0-i(\gamma_0+\gamma_1) \end{pmatrix}. \tag{S15}$$

This is the Hamiltonian given in Eq. (4) in the main text.

Obviously, the nonlinear coupling $\tilde{\kappa}_2$ originates from the nonlinear behavior of the amplifier (i.e., Y_{21}). Supplementary Fig. 3b shows the measured gain (or amplification ratio) and phase of the amplifier as a function of input power. For weak input power, the gain seems stable. Nonetheless, as the input power increases beyond a certain threshold, gain compression occurs. The measured data also fit well with Eq. (S8) and Eq. (S9) (see solid curves in Supplementary Fig. 3b).

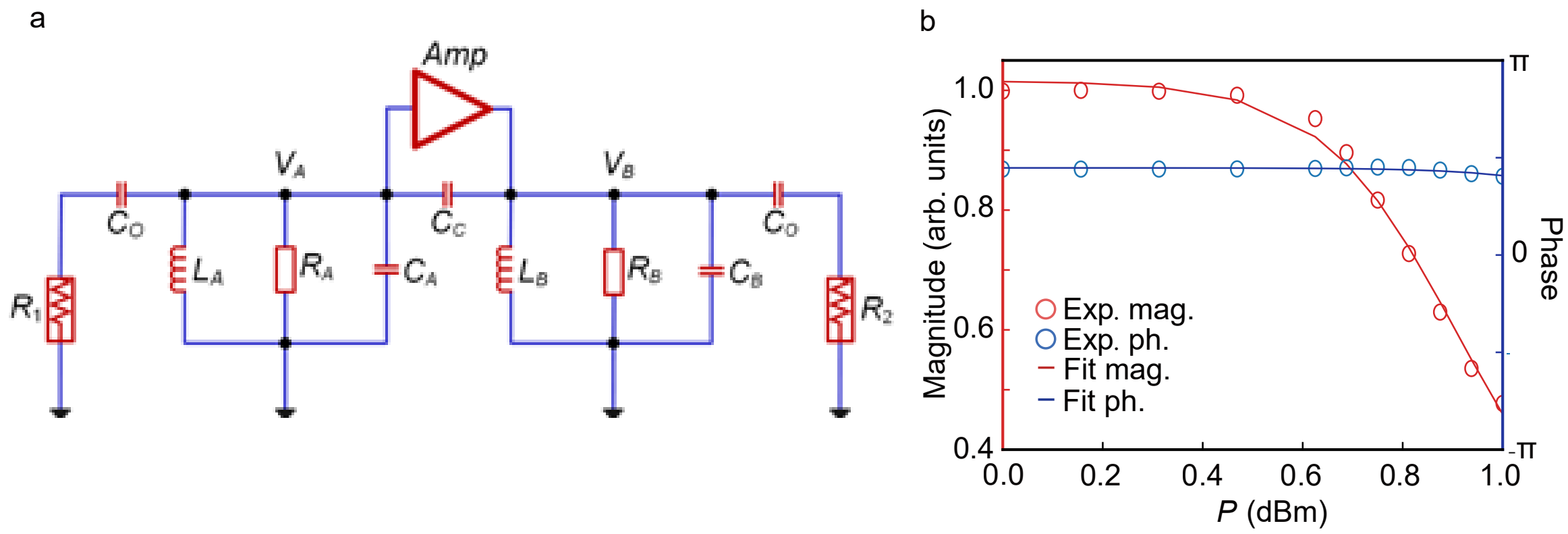

Supplementary Fig. 3 Circuit model representation. a, The equivalent RLC model of the two-resonator system. The source and detector introduce inner resistors R_1, R_2 and coupling capacitor C_O. R_X, L_X, C_X with $X = A, B$ are properties of the resonators. Reciprocal coupling involves C_C, while an amplifier facilitates nonreciprocal coupling. b, Measured magnitude (red dots) and phase (blue dots) of the amplifier under different input powers. The solid curves are the corresponding fit using the nonlinear model.

SUPPLEMENTARY NOTE 4: EXPERIMENTAL DETAILS OF RESONATOR COMPONENTS

The microwave resonators in this work are fabricated with printed circuit board (PCB) technology. Both sides of the microstrip board are coated with 35-μm-thick copper layers, and the dielectric substrate is composed of 0.8-mm-thick FR4 material with a dielectric constant of 4.6 and a loss tangent of 0.02. The resonator is H-shaped [7], where each section has the same length (2 cm), width (0.1 cm) and characteristic impedance (about 64 Ω). The unloaded resonance frequency (1.2 GHz) is decreased by coupling capacitors. A resistor is patched in the middle of the resonator for system stability. The LNA chip (MGA-53543) is designed with a bias circuit while back-to-back varactors (SMV2201) are blocked with an isolated inductor. Supplementary Fig. 4 depicts the circuit elements in the sample. Their specific values are $R_1 = 1\,\Omega$, $R_2 = 2.2\,\Omega$, $L_1 = 39\,\text{nH}$, $L_2 = 15\,\text{nH}$, $C_1 = 0.8\,\text{pF}$, $C_2 = 0.6\,\text{pF}$, $C_3 = 1000\,\text{pF}$. Non-Hermiticity is enhanced by introducing resistor R_1. Capacitor C_2 transmits the signal between the resonator and amplifier. Inductors L_1 and L_2 effectively isolate the RF signal, allowing only the DC bias voltage to pass through. R_2 and C_3 form a filtering circuit, which effectively suppresses noise from active chips. Several practical limitations of the current setup should be noted. Firstly, variations in discrete

components (particularly capacitors) introduce disorder in the coupling parameters. While moderate disorder does not qualitatively alter the observed phenomena, sufficiently large fluctuations may affect the nonlinearity-induced topological phase transitions [9]. Secondly, the nonlinear response is governed by the characteristics of the RF amplifier. In the present implementation, the high-linearity MGA-53543 amplifier provides a controlled saturable response with measurable third-harmonic generation. Different amplifier types with narrower linear ranges may result in stronger harmonic content and a reduced controllable regime. Lastly, signal noise must be minimized to ensure system stability, especially at high input power. Otherwise, large power fluctuation or uncontrolled power growth can invalidate the theoretical modelling and even damage the sample.

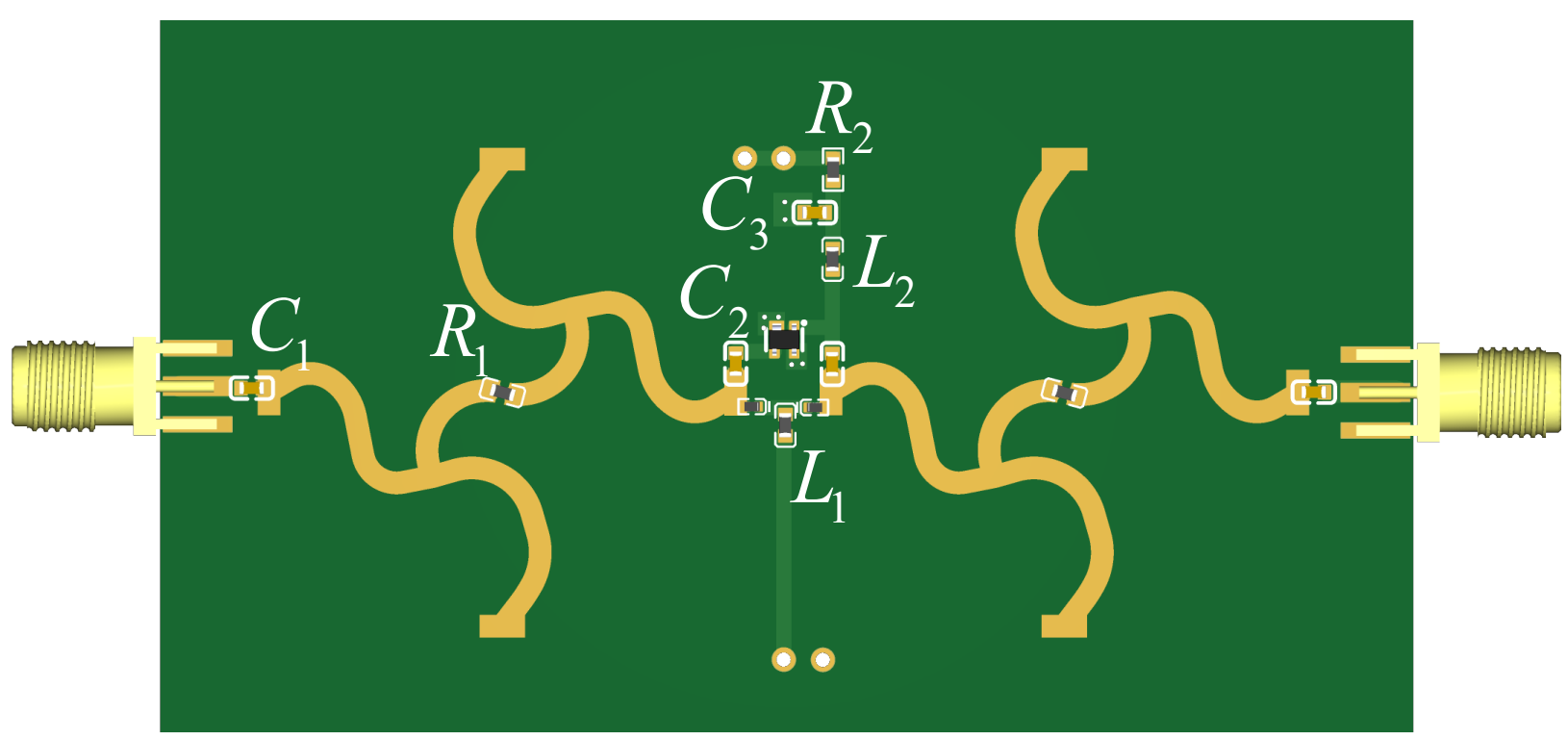

Supplementary Fig. 4 Sample details.

SUPPLEMENTARY NOTE 5: HARMONICS FROM NONLINEARITY

Nonlinear response and harmonic generation mechanism.

In this section, we present a simple model to understand the harmonic generation mechanism in our system intuitively. The nonreciprocal coupling is implemented using an LNA structure, which generates frequency components of $n\omega_0$ ($n = 2, 3...$) when the amplitude of the input signal is high. The general output response of a nonlinear circuit can be expressed using the Taylor series [10] as:

$$v_o = a_0 + a_1 v_i + a_2 v_i^2 + a_3 v_i^3 + \cdots \tag{S16}$$

where v_i and v_o denote the time-domain input and output signals, respectively. The response is characterized by the following coefficients: a_0 is the DC offset component, a_1 is the fundamental linear gain, and a_i $(i > 1)$ are the nonlinear higher-order harmonic coefficients. Since LNAs typically work with low input power, the contribution of higher-order terms is less significant than that of lower-order terms. Therefore, the output can be approximated by the first three power series terms. As symmetrical saturation suppresses the even harmonics, a_2 is neglected [11]. Taking the input v_i as a sinusoidal input,

$$v_i = A\cos\omega_0 t. \tag{S17}$$

The output response v_o can be obtained as,

$$v_o = a_0 + \left(a_1 A + \frac{3a_3A^3}{4}\right)\cos\omega_0 t + \frac{a_3A^3}{4}\cos 3\omega_0 t. \tag{S18}$$

When the chip works in saturation, a_1 and a_3 have opposite polarities. With low input power, the third term is negligible, maintaining linear gain a_1. However, when the input power increases, the fundamental term decreases due to gain compression, while the third-order term rises sharply. Consequently, the third harmonic becomes significant and should be taken into account. The electrical field distributions of the fundamental and third harmonics are depicted in Supplementary Fig. 5. Here, the third-harmonic distribution directly inherits the reversal of the fundamental harmonic. Since there are no eigenmodes at the third harmonic frequency, the higher-order harmonic distribution is governed by the fundamental mode, enabling nonlinear control of its spatial localization.

Time-domain characterization and steady-state response.

To visually verify the aforementioned nonlinear saturation effects and ensure the reliability of our spectrum measurements, we record the time-domain transient response of the system under discrete power steps at port 11 (Supplementary Fig. 6). The 1 GHz excitation is generated by a Siglent SSG5060X-V signal generator, and the time-domain response is recorded using a Tektronix DPO7254 series digital phosphor oscilloscope (4 GHz bandwidth and 20 GS/s sampling capability).

For the measurements shown in Supplementary Fig. 6, the oscilloscope is operated in a long time-window mode (40 ms/div, record length 20000 points) to capture the slow response dynamics under discrete power steps. Under these settings the carrier at 1 GHz is not cycle-resolved. Instead, the recorded trace reflects the slow-time evolution of the signal amplitude.

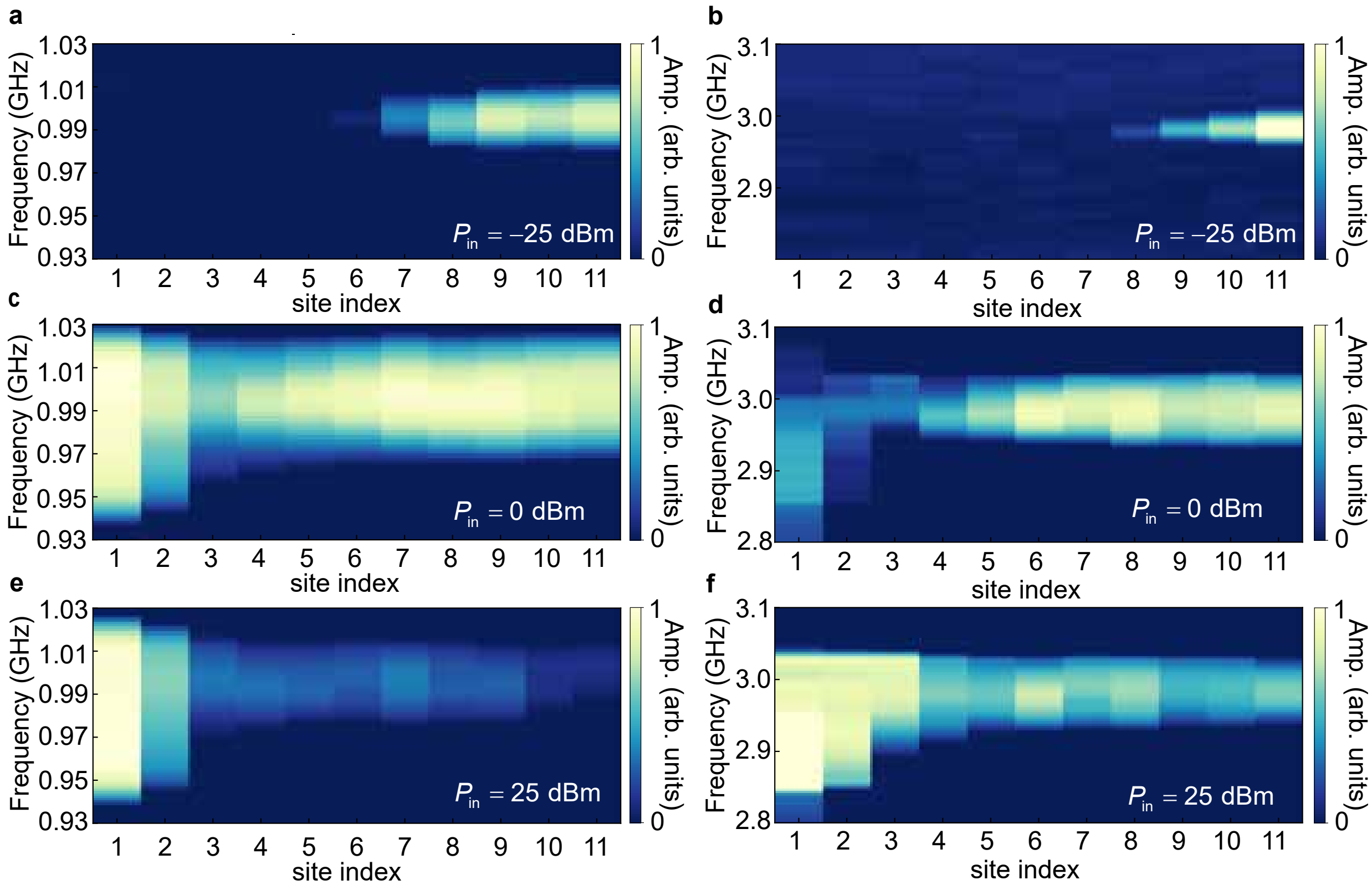

Supplementary Fig. 5 Observation of harmonics reversal of the NHSE via port measurements. Measured spatial field distributions of the fundamental harmonic at input powers of $P_{\text{in}} = -25\,\text{dBm}$ (a), $0\,\text{dBm}$ (c), and $25\,\text{dBm}$ (e). Corresponding spatial field distributions of the third harmonic at $P_{\text{in}} = -25\,\text{dBm}$ (b), $0\,\text{dBm}$ (d), and $25\,\text{dBm}$ (f). Each panel is independently normalized to its maximum response at the corresponding input power.

For a narrowband RF signal,

$$v(t) = A(t)\cos(2\pi f_0 t + \phi(t)), \tag{S19}$$

where $f_0 \approx 1$ GHz and $A(t)$ varies on the millisecond timescale, a long time-base measurement effectively tracks the envelope amplitude. Therefore, the plotted waveform should be interpreted as the slow-time intensity evolution of the 1 GHz signal. Importantly, the present configuration is not intended to reconstruct the carrier waveform within each RF cycle. The analysis focuses exclusively on steady-state amplitude levels and their dependence on the input power, which are fully encoded in the slow-time envelope. The magnitude of the envelope is recorded for input powers ranging from -30 dBm to 20 dBm. As summa-

Input Power (dBm)	Normalized Amplitude (dB)
-30	0.000
-20	1.393
-10	7.729
0	15.933
10	20.862

Supplementary Table 2 Normalized steady-state output amplitude referenced to the value at −30 dBm input.

rized in Supplementary Table 2, the port voltage exhibits a nonlinear response to the input excitation. At 20 dBm input power, noticeable waveform distortion appears at both the peaks and troughs, indicating clear nonlinear compression. Additionally, the signal settles within approximately 1 ms after the intensity is switched, ruling out a transient origin of the observed effect.

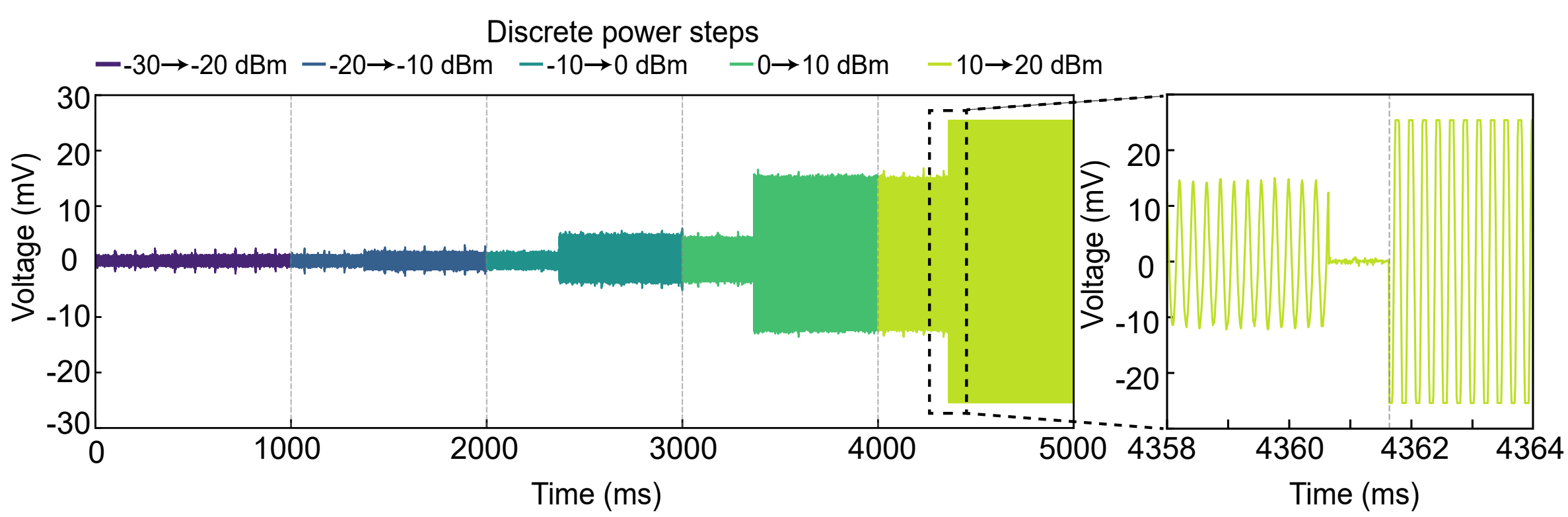

Supplementary Fig. 6 Long time-base transient response of port 11 under discrete power steps. The trace represents the slow-time evolution of the signal amplitude.

SUPPLEMENTARY NOTE 6: ROBUSTNESS OF THE LOCALIZATION REVERSAL

In this section, we show that the reversal of the NHSE is a robust phenomenon. For this purpose, we calculate the average position $\bar{x}_c$ (defined in Eq. (3) in the main text) of

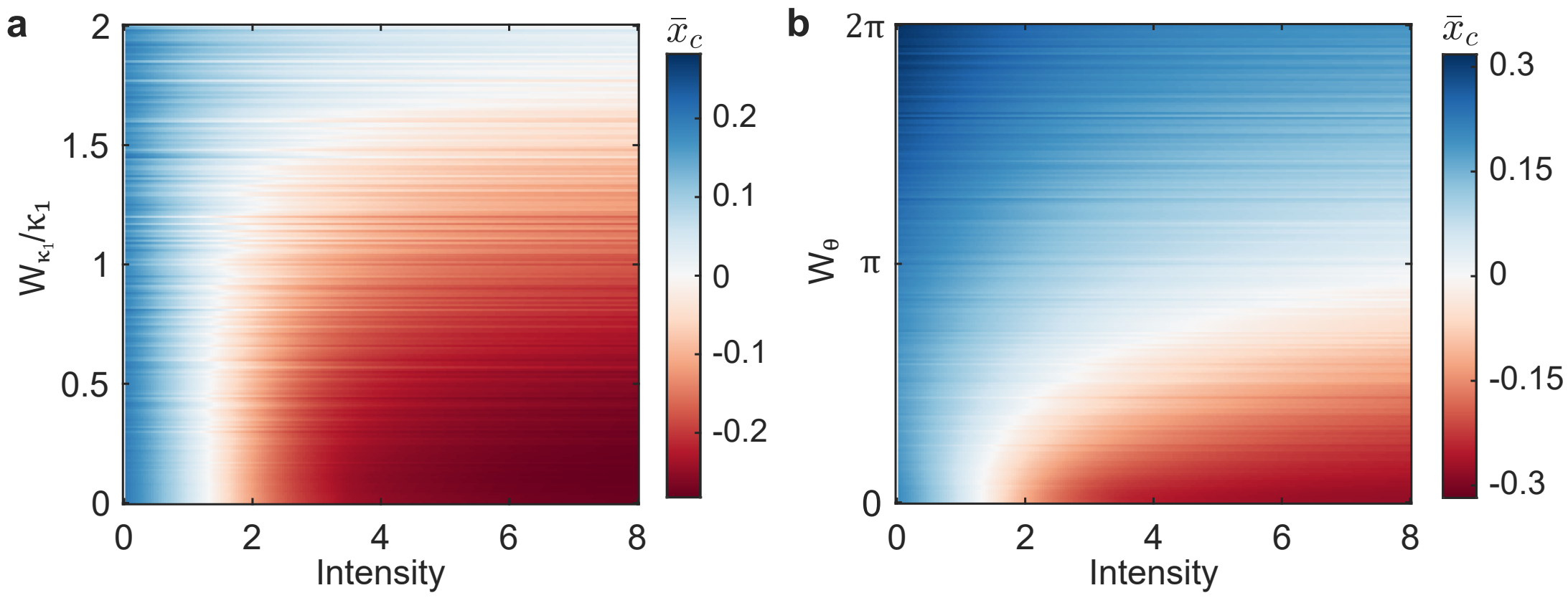

Supplementary Fig. 7 Robustness of the localization reversal. Plots of the average position of the OBC eigenmodes as a function of total intensity and disorder strength. a, Disorder in reciprocal coupling κ_1. b, Disorder in coupling phase θ. Parameters are consistent with those in Fig. 1 of the main text. The results are averaged over 100 disorder realizations.

the OBC eigenmodes as a function of the total intensity and the disorder strength. We consider two types of Anderson-type disorder: (1) disorder in the reciprocal coupling κ_1, introduced as $\kappa_1' = \kappa_1 + \alpha$ with α a random number drawn from $[-W_{\kappa_1}/2, W_{\kappa_1}/2]$; (2) disorder in the coupling phase θ, introduced as $\theta' = \theta + \beta$ with β a random number drawn from $[-W_\theta/2, W_\theta/2]$. Here, W_{κ_1} and W_θ serve as the corresponding disorder strength. As shown in Supplementary Fig. 7, the reversal of the NHSE persists when the disorder strength is below approximately $1.74\kappa_1$ (Supplementary Fig. 7a) and 0.91π (Supplementary Fig. 7b), respectively, demonstrating strong robustness of this phenomenon.

SUPPLEMENTARY NOTE 7: CHARACTERIZATION OF THE SKIN DEPTH

While the main text characterizes the localization direction of the NHSE by calculating the mean position, here we further compute the skin depth to quantify the degree of localization. The skin depth ξ is obtained by fitting the wavefunction profile to the form $\psi(x) \sim e^{-x/\xi}$, where a smaller ξ corresponds to stronger localization, and a larger ξ indicates weaker localization. For the left- and right-localized skin modes, we choose the corresponding boundary site as the fitting origin to ensure all ξ values are positive. The results in Supplementary Fig. 8 demonstrate a consistent trend between theoretical calculations and

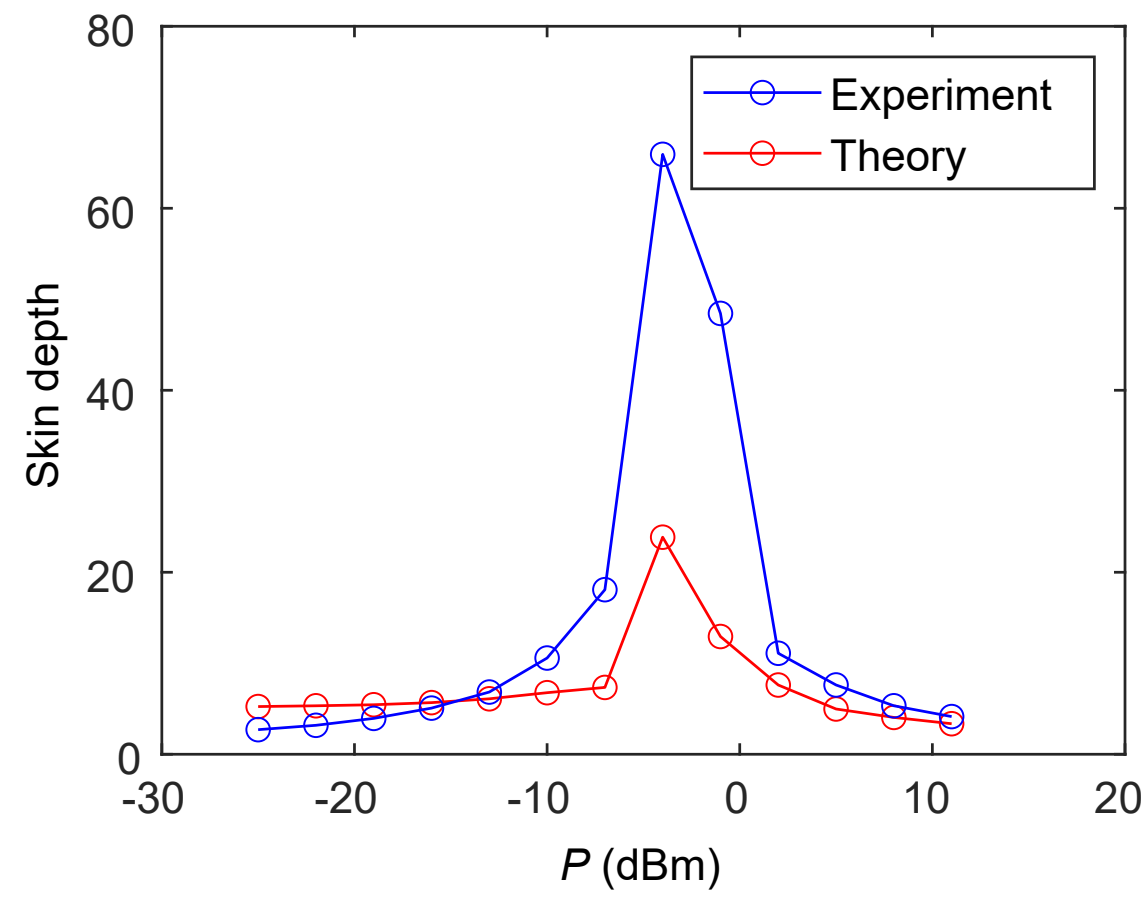

Supplementary Fig. 8 Skin depth. The skin depth extracted from theory and experiment as a function of input power.

experimental measurements. The peaks in the curves, corresponding to large skin depths, serve as hallmarks of the localization reversal.

The skin depth ξ characterizes how far a skin mode extends from the localization boundary, defined through the exponential decay

$$\psi(x) \sim e^{-x/\xi}, \tag{S20}$$

where x is the distance from the localization boundary. A smaller ξ means the mode is strongly confined near the boundary, while a larger ξ indicates a more spatially extended mode. At the topological phase transition point, ξ diverges, meaning the mode becomes completely delocalized. Beyond this point, the mode relocalizes at the opposite boundary. To extract ξ, we take the natural logarithm of both sides:

$$\ln \psi(x) = -\frac{1}{\xi}\, x + C, \tag{S21}$$

where C is a constant. This transforms the exponential decay into a linear relation. A least-squares linear fit to $\{(x_j, \ln \psi_j)\}$ across all lattice sites yields the slope k, from which the skin depth is obtained as $\xi = -1/k$. For each input power, the localization boundary is identified as the site with the maximum field amplitude.

SUPPLEMENTARY NOTE 8: NONLINEAR NONRECIPROCAL SSH MODEL

In this section, we present the theoretical framework for the nonlinear nonreciprocal SSH model discussed in the main text. The model, schematically shown in Fig. 5a in the main text, consists of a one-dimensional dimerized chain with alternating intracell and intercell couplings. Each unit cell contains two sites, labeled A and B. The coupled-mode equations for the field amplitudes $\Psi_{n,\mathrm{A}}$ and $\Psi_{n,\mathrm{B}}$ at unit cell n are given by

$$i\frac{d\Psi_{n,\mathrm{A}}}{dt} = f_0\Psi_{n,\mathrm{A}} + t_l\Psi_{n,\mathrm{B}} + t_w\Psi_{n-1,\mathrm{B}}, \tag{S22}$$

$$i\frac{d\Psi_{n,\mathrm{B}}}{dt} = f_0\Psi_{n,\mathrm{B}} + t_r(I_{n,\mathrm{A}})\Psi_{n,\mathrm{A}} + t_w\Psi_{n+1,\mathrm{A}}, \tag{S23}$$

where f_0 is the resonance frequency, t_l is the linear leftward intracell coupling, and t_w is the linear reciprocal intercell coupling. The nonlinear rightward intracell coupling t_r consists of two contributions: one is the coupling arising from reciprocal capacitance with an amplitude equal to t_l, and the other is the unidirectional saturable nonlinearity whose form is given by Eq.(2) in the main text. We thus obtain:

$$t_r(I_{n,\mathrm{A}}) = \left(\frac{t_0 - t_\infty}{1 + I_{n,\mathrm{A}}/t_c} + t_\infty\right)e^{i\theta} + t_l, \tag{S24}$$

where $I_{n,\mathrm{A}} = |\Psi_{n,\mathrm{A}}|^2$ is the field intensity at site A of unit cell n, and t_0, t_∞, t_c, θ are defined as in Eq. (2) of the main text. Note that the nonreciprocal coupling t_r is unidirectional (from site A to site B), consistent with the amplifier-based implementation described in the main text.

Searching for time-harmonic solutions of the form $\Psi_{n,\mathrm{A}}(t) = \psi_{n,\mathrm{A}}e^{-iEt}$ and $\Psi_{n,\mathrm{B}}(t) = \psi_{n,\mathrm{B}}e^{-iEt}$, the coupled-mode equations reduce to the nonlinear eigenproblem $H\psi = E\psi$, where $\psi = (\psi_{1,\mathrm{A}}, \psi_{1,\mathrm{B}}, \psi_{2,\mathrm{A}}, \psi_{2,\mathrm{B}}, \ldots, \psi_{M,\mathrm{A}}, \psi_{M,\mathrm{B}})^T$ and M is the number of unit cells. The Hamiltonian for a finite chain under open boundary conditions is

$$H = \begin{pmatrix} f_0 & t_l & & & & \\ t_r^{(1)} & f_0 & t_w & & & \\ & t_w & f_0 & t_l & & \\ & & t_r^{(2)} & f_0 & \ddots & \\ & & & \ddots & \ddots & t_l \\ & & & & t_r^{(M)} & f_0 \end{pmatrix}, \tag{S25}$$

where $t_r^{(n)} \equiv t_r(I_{n,\mathrm{A}})$ denotes the nonlinear intracell coupling at unit cell n, which depends on the local field intensity.

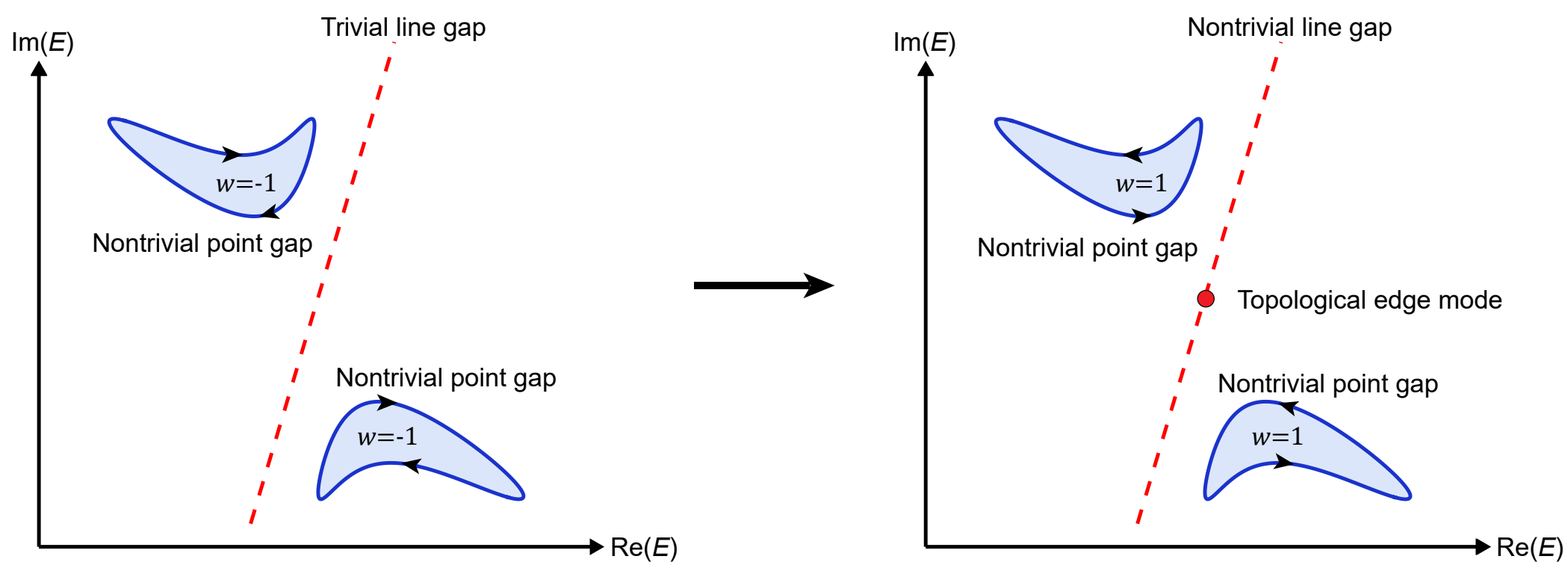

Supplementary Fig. 9 Schematic illustration of point gap and line gap in the nonlinear non-reciprocal SSH model. Left: trivial line-gap phase with winding number $w = -1$. Right: nontrivial line-gap phase with $w = +1$ after the point-gap transition. Details are provided in Supplementary Note 8.

In such nonlinear nonreciprocal SSH models, both point-gap and line-gap topological phase transitions can be realized. As shown in Supplementary Fig. 9, under our initial parameter settings, the intracell couplings satisfy $|t_r| > |t_l|$, so the NHSE is initially localized on the right side, corresponding to a winding number $w = -1$. Meanwhile, the condition $|t_r| \cdot |t_l| > |t_w|^2$ places the system in a trivial line-gap phase. As $|t_r|$ decreases with the increase of site intensity, the NHSE reverses, corresponding to a winding number $w = 1$, a signature of the point-gap phase transition. Furthermore, the reduction of $|t_r|$ induces the emergence of topological edge states, signaling the occurrence of the line-gap phase transition. This emergence is experimentally verified by the opposite-localization signature on odd and even sublattices, as observed in Fig. 5g of the main text.

SUPPLEMENTARY REFERENCES

* These authors contributed equally to this work.

† yangyihao@zju.edu.cn

‡ haoranxue@cuhk.edu.hk

[1] Z. Gong, Y. Ashida, K. Kawabata, K. Takasan, S. Higashikawa, and M. Ueda, Topological phases of non-hermitian systems, Phys. Rev. X **8**, 031079 (2018).

[2] E. N. Economou, *Green's Functions in Quantum Physics*, Springer Series in Solid-State Sciences (2013).

[3] Y. Ashida, Z. Gong, and M. Ueda, Non-Hermitian physics, Adv. Phys. **69**, 249 (2020).

[4] C. Mahaux and H. A. Weidenmüller, *Shell-Model Approach to Nuclear Reactions* (1969).

[5] L. Zhang, Y. Yang, Y. Ge, Y.-J. Guan, Q. Chen, Q. Yan, F. Chen, R. Xi, Y. Li, D. Jia, *et al.*, Acoustic non-Hermitian skin effect from twisted winding topology, Nat. Commun. **12**, 6297 (2021).

[6] S. Assawaworrarit, X. Yu, and S. Fan, Robust wireless power transfer using a nonlinear parity-time-symmetric circuit, Nature **546**, 387 (2017).

[7] C. W. Peterson, W. A. Benalcazar, T. L. Hughes, and G. Bahl, A quantized microwave quadrupole insulator with topologically protected corner states, Nature **555**, 346 (2018).

[8] K. Bai, T.-R. Liu, L. Fang, J.-Z. Li, C. Lin, D. Wan, and M. Xiao, Observation of nonlinear exceptional points with a complete basis in dynamics, Phys. Rev. Lett. **132**, 073802 (2024).

[9] B.-B. Wang, Z. Cheng, H.-Y. Zou, Y. Ge, K.-Q. Zhao, Q.-R. Si, S.-Q. Yuan, H.-X. Sun, H. Xue, and B. Zhang, Observation of disorder-induced boundary localization, Proc. Natl. Acad. Sci. **122**, e2422154122 (2025).

[10] H. Zhang and E. Sánchez-Sinencio, Linearization techniques for CMOS low noise amplifiers: A tutorial, IEEE Trans. Circuits Syst. I: Regul. Pap. **58**, 22 (2010).

[11] J. W. M. Rogers and C. Plett, *Radio Frequency Integrated Circuit Design* (2010).
