# Supplementary material for: Observation of localization reversal and harmonic generation in nonlinear non-Hermitian skin effect
Source: Nat Commun. 2026 Jun 26;17:8028. doi: 10.1038/s41467-026-74610-y (PMC13454480; doi:10.1038/s41467-026-74610-y)
Supplement: Supplementary file 2 [file 41467_2026_74610_MOESM2_ESM.pdf]

nature portfolio

Peer Review File

Observation of localization reversal and harmonic generation in nonlinear non-Hermitian skin effect

Corresponding Author: Dr Haoran Xue

This file contains all reviewer reports in order by version, followed by all author rebuttals in order by version.

Version 0:

Reviewer comments:

Reviewer #1

(Remarks to the Author)
In this manuscript the authors present experimental demonstration of a nonlinear and non-Hermitian system which consists of coupled microwave resonators. In particular, they demonstrate nonlinearity-induced topological phase transition (with reversal of the skin mode localization): this is enabled due to a specially engineered nonlinear coupling which exists only along one direction. Apart from reversal of the skin mode localization, the authors also demonstrate harmonic generation produced by skin modes.

I find the manuscript to be very interesting (and timely) to the community, as it presents a convenient and robust platform for the study of the interplay between non-linearity and non-Hermicity, in particular by enabling the non-reciprocal coupling to be nonlinear. Numerical analysis and experimental results are clear and convincing.

I do have few minor comments, as follows. Large emphasis in the paper is given to the elaboration of the theoretical model and investigation of its properties, and this is good for the clarity of the paper. However, the most important part of this paper is that it shows that one can have nonlinear nonreciprocal couplings in a system of microwave resonators. I think this should be elaborated in more details even in the main text. At present, the mechanism which enables it is somewhat hidden in the supplementary material, and I think it could be explained more clearly. Secondly (and related to this), the authors should discuss the possibility of extending their setup from 1D to 2D; I think this would be important to make the paper even more relevant. Lastly, it would be beneficial if the authors could comment on other limitations of the setup (e.g, maximum power/nonlinearity, noise etc.).

To conclude, I find the work presented here to be important and results to be excellent. My recommendation is that the manuscript should be published in Nature Communications.

Reviewer #2

(Remarks to the Author)
In this manuscript, the Authors report an experimental study of the nonlinear non-Hermitian skin effect. While the topic may be of interest to a relatively specialized community (it is not actually a new one), the manuscript appears to me largely unreadable and unreproducible. Therefore, I cannot recommend it for publication. My main (although not all) reasons are summarized below.

1. Equation (1) introduces creation and annihilation operators, but their physical meaning is not specified. It is unclear what these operators are meant to create or annihilate, whether they are bosonic or fermionic, and what commutation relations they satisfy. Moreover, their connection to the experimental setup is not explained. The manuscript does not discuss the role of quantum effects or justify the use of a classical approximation. This lack of clarification makes it difficult to understand how the theoretical model relates to the experiment.

2. The statement “Without loss of generality, we set the linear reciprocal coupling as unity’’ (before Eq. (2)) is questionable. In nonlinear systems, rescaling coupling coefficients is not generally innocuous: modifying the magnitude of these coefficients typically requires a corresponding rescaling of the field amplitudes, which changes physically relevant

parameters such as effective nonlinearity. Therefore, setting this coefficient to unity does reduce generality, and the authors should justify why this simplification does not affect the validity of their conclusions.

3. In Eq. (2), the field intensities I_i are introduced, yet their relation to the creation and annihilation operators appearing in Eq. (1) is not explained. This link is crucial for the system to be regarded as nonlinear.

4. In the second paragraph on p. 3 the Authors introduce the eigenvalue problem \hat{H}\psi=E\psi. \psi is not defined here. What is it?

5. Formula (3) appears to be valid only for odd N. This restriction must be clearly stated. If the authors use this expression exclusively for the case N=21, then writing it in a “general” form is somewhat misleading. In that case, it would be more appropriate to present the formula specifically for N=21 rather than implying general validity that does not hold.

6. A more serious issue concerns the mismatch between experiment and numerics. The experiment is performed with N=11 sites (Fig. 3a), yet theoretical calculations are carried out for N=21sites. This choice does not provide additional generality; instead, it creates a discrepancy between the numerical and experimental results.

7. In the same paragraph, when discussing the threshold there appear formulas that follow from nowhere. Where do they come from?

8.In general, the transmission problem and the eigenmode problem are distinct (although related). In Fig. 2(d), the authors present transmission coefficients, yet these coefficients are neither defined nor discussed in the main text.

9. I do not understand the meaning of the color plots in Figures 3b to 3d. What does f represent? Usually, frequency is a characteristic of a mode rather than a site. Without clear explanations, the figure appears meaningless. In the supplementary information the authors say that f “is the frequency of the driving signal “. But there is no driving term in the Hamiltonian (1) nor f can be an eigenvalue.

10. Turning now to the supplementary information, where does (S1) come from? What is the equation for the Green's function? The authors neither provide references where this formula can be found nor derive it.

11. In the main text the Authors use \hat{H} while in (S1) they use H. What is the relation between these two quantities?

12. Where do formulas (S2) - (S4) come from? How can they be computed?

13. In (S7) the Authors say “Assuming a time dependence of…”. Of what? Furthermore, what is the relation between the frequencies \omega and f?

14. It is not explained how Y_{21} appers in (S7) and how it is related to the rest of the parameters used in the main text. Also why are Y_{ii} small?

15. What are v_0 and v_i in (S13)? What are a_i? All these quantities are not defined.

16. I cannot understand this phrase “As symmetrical saturation suppresses the even harmonics,” What does “saturation” mean here?

17. The whole discussion in Supplementary note 4 might be meaningful only if A is small enough. What does “low input power” mean in mathematical terms?

18. I do not understand why the authors use round brackets (..) in some places and square brackets [..] in others. Are these parts written by different co-authors and noone in the team could unify notations, or is there a more significant reason for this distinction?

To conclude, hindered by all the problems listed above, I could not find any discussion of new physics that this work might reveal.

Reviewer #3

(Remarks to the Author)
The authors report an experimental observation of a nonlinearity-induced reversal of the non-Hermitian skin effect (NHSE) in a microwave metamaterial platform, accompanied by harmonic generation. The work addresses an interesting and timely direction at the intersection of nonlinearity and non-Hermitian topology. The experimental platform is well developed, and the results are potentially impactful. However, in its current form, the manuscript requires substantial clarification and strengthening before it can be considered for publication.

Below, I provide major and minor comments that I believe will help improve the clarity, rigor, and interpretability of the work.

The introduction motivates nonlinear non-Hermitian physics, but the manuscript should more clearly state what is fundamentally new about localization reversal compared to previously known nonlinear effects (e.g., self-induced

topological transitions, nonlinear spectral shifts).

1) The manuscript states that a nonlinearity-induced point-gap topological transition underlies the observed localization reversal. However, the physical origin and mathematical structure of this nonlinear effect remain under-specified.

a) Please clarify what specific nonlinear mechanism is active in the circuit: is it effectively Kerr-type, saturable, gain-modulating, or coupling-modifying?

b) Can the authors derive or present a minimal effective Hamiltonian showing how nonlinearity drives a topological transition?

c) The connection between nonlinearity and point-gap topology should be more explicit. For example: How do the winding number or point-gap invariants evolve with pump intensity? Is the reversal strictly tied to a change in a topological index, or can it occur via a more conventional nonlinear redistribution of gain/loss?

d) Fig 1 (f) the OBC spectrum has a pleatau along the real line. Does it signify something?

e) It would be interesting to know, when there are both topological modes and skin modes(like in non-reciprocal SSH model), how the localization reversal phenomena would work?

2) The reported reversal of skin localization is the central experimental signature, but alternative explanations must be addressed.

a) Could pump-induced gain/loss redistribution mimic a localization reversal even without a topological transition?

b) How robust is the effect to structural disorder, component tolerances, or asymmetry in the circuit?

c) Please quantify the localization length/skin depth (experiment vs theory) as a function of pump power.

3) It is unclear whether the system reaches a steady state before measurements. How long after adjusting the pump do the authors wait to measure? Are there transient or hysteretic effects that could complicate interpretation? Are there threshold intensities or hysteresis effects? For example, once the localisation has switched, does reducing the pump cause reversal back to the original end, or is there memory/hysteresis?

4)The authors claim this opens “a new route towards nonlinear topological physics in non-Hermitian systems”. It may help to clarify the boundaries of that claim: is the effect specific to microwave metamaterials, or could it also apply in, e.g., photonic circuits, acoustic systems, exciton–polariton systems? A short paragraph discussing generality and limitations would help.

5) It might be useful to comment on how the effect scales: for larger system sizes, different strengths of nonlinearity, or in higher dimensions, would one expect similar reversal behaviour?

6) Are there trade-offs or regimes where the nonlinearity could destroy the skin effect (rather than reverse it)?

7) While harmonic generation is highlighted in the title, its connection to NHSE reversal is not clearly established.

a) Are higher harmonics also subject to localization reversal?

b) Do harmonics carry their own topological signatures, or are they by-products of nonlinearity?

c) Please include comparative spectra of fundamental and harmonic intensities across the pump-induced transition.

Additional Minor but Helpful Suggestions

a) Define “localization reversal” more precisely early in the paper--does it refer to mode center shifting, change in decay direction, or a sign change in the skin depth?

b) Improve figure captions by including pump intensity values, normalization conventions, and experimental conditionEnsure consistency in notation between theory and experiment sections (e.g., use same symbols for nonlinear strength)

c) The authors may Include a schematic of how the nonlinearity modifies the spectrum in the complex plane with proper winding--this would make the topological picture much clearer.

Version 1:

Reviewer comments:

Reviewer #2

(Remarks to the Author)

In the revised version of the manuscript, the authors address some of my concerns. However, they did not attempt to address my main concern—namely that I could not find any discussion of what new physics this work might reveal. Without this clarification, the work appears primarily technical.

Moreover, the authors have introduced new material that, in my view, deviates significantly from the original content and makes the work even less reproducible than it was before the revision. Even more surprising is the removal of formulas that seem important, such as Y_{ii} (formerly in Eq. (S7)). This gives the impression that at least some of the equations serve a decorative rather than a substantive role in the manuscript; otherwise, it is difficult to understand why they would be omitted or left undefined (see below).

Starting with the last point: in Note 1 the authors define the winding number in Eq. (S1) using a “reference point” E_B. This reference point is not defined anywhere in the main text or in the Supplementary Note, and the specific value of E_B used to produce Fig. S1 is not stated. More importantly, $H\left(\Phi\right)$ appearing in Eq. (S1) is not defined. What is it supposed to represent and how computed? In the main text (Methods) one finds $H\left(\psi\right)$ which is clearly a different object, because Φ and ψ denote entirely different quantities (an argument and a field, respectively). As written, a reader has no way to reconstruct how Eq. (S1) is obtained or how Fig. S1 was computed.

The new paragraph entitled “Nonlinear nonreciprocal SSH model” is even more obscure. It appears to describe a different setting (schematically shown in Fig. 5a), yet it provides only general statements and a few plots in Fig. 5, without the supporting formulas, definitions, or theoretical framework needed to connect the model to the displayed results. As a consequence, a reader cannot follow the authors’ reasoning or reproduce the results presented in this paragraph.
Further, in Note 7 the authors claim that they fitted “the wave-function profile near the localization peak” with an exponential form. However, they do not show the fit itself. Instead, Fig. S8 shows a rather poor agreement between theory and experiment: the predicted peak depth is more than three times smaller than the experimental value. Moreover, even this limited comparison cannot be reproduced, because the authors provide no fitting procedure, parameters, fitting window, or other essential details.

Now I revise answers to some of my points (with corresponding numbering).

2. I am afraid the authors did not get the point. “Without loss of generality” means that a parameter can be scaled out completely (or eliminated by a redefinition of variables), so that it does not affect the generality of the results. This is not the same as choosing a value that is merely “typical” or representative.

What Fig. R1 illustrates is that the chosen parameters may be typical within some range, but it does not demonstrate that the conclusions hold in full generality. To justify “without loss of generality,” the authors would need to show an explicit scaling argument (or prove invariance of the relevant dimensionless quantities), rather than only providing a numerical example or a parameter sweep over a limited interval.

Furthermore, later in the text the parameter κ_1 appears agin, What is it, considering that it was set “without loss of generality” $\kappa_{1,n}=1$?

5. Eq. (3) does not look meaningful as written. It is unclear what is meant by “the average over all modes” <.>? How is it computed exactly? This is particularly problematic in the nonlinear case: the number of stationary solutions can exceed that of the linear limit (bistability being the simplest example). In that situation, “average over all modes” is ambiguous unless the authors explain which branch(es), if there are more than one, are included.

If, instead, the authors actually mean an average over the linear eigenmodes only, then $\bar{x}_c$ is just a fixed number determined by the linear spectrum and should be explicitly provided, along with the precise procedure used to compute it. I emphasize that I am talking about averaging <.>, rather than about trivial formula (R3).

7. The parameter κ_1 is not defined in the text.

8. The Mahaux–Weidenmüller formula is typically derived for linear systems and is not applicable to nonlinear ones. Therefore, the meaning of the Note 2 remains unclear, unless the authors can justify the use of the Mahaux–Weidenmüller formula for a nonlinear system, or provide a reference where such an extension is derived.

10. The authors’ justification has a problem. In general, Fourier analysis is not directly applicable to nonlinear systems such as the one considered.

12. Remains the nonlinearity issue mentioned above.

14. Quantity Y_{ii} disappeared from the new SI without explanations why.

15. v_0 and v_i in the SI are not related to any of the quantities in the main text, thus their meaning in the context of the present work remains unexplained

In summary, I do not consider the present work suitable for publication.

Reviewer #3

(Remarks to the Author)
I thank the authors for their careful revisions. I am generally satisfied with their responses, and I believe they have made a genuine effort to address the referee reports and improve the clarity and overall quality of the manuscript. That said, I feel that the draft would still benefit from additional clarification and justification. I outline a few remaining points below:

The authors state that “the peaks in the curves, corresponding to large skin depths, serve as hallmarks of the localization reversal.” This statement would benefit from a more explicit explanation. As it stands, the connection is not entirely clear. My understanding is that a large skin depth corresponds to a stronger non-Hermitian skin effect, i.e., a higher degree of localization toward the boundary. It would therefore be helpful if the authors could clarify how an increase in skin depth signals a localization transition or reversal, and clearly distinguish this from conventional notions of localization strength.

The inclusion of the non-linear non-reciprocal SSH model is a valuable addition. However, the discussion of the associated figures (in particular Fig. 5) requires further clarification:

i) Do the topological edge states correspond to zero real energy? If so, it is not immediately clear how this can be identified from Fig. 5(b). A more explicit explanation in the text would be helpful.

ii) The discussion of line-gap and point-gap transitions, and their respective effects on edge states and skin modes, remains somewhat opaque. A more transparent and pedagogical explanation would significantly improve readability. The authors state that “the skin modes are localized around the right end (Fig. 5c,e).” It would be useful to clearly indicate whether both panels (c) and (e) indeed represent skin modes, and if so, how they differ from each other.

iii) The notion of a “line gap” in the present context is not sufficiently clear. Does this refer to a situation where two spectral loops are separated along the real-energy axis? A more explicit definition, possibly accompanied by a schematic illustration or more detailed discussion in the text, would greatly aid the reader.

Overall, while the manuscript has improved, addressing the points above would further enhance its clarity and accessibility.

Version 2:

Reviewer comments:

Reviewer #3

(Remarks to the Author)
I have reviewed the authors’ revised manuscript and their detailed response to my previous comments. I am satisfied that all of my concerns have been thoroughly addressed. In particular, I think that the findings presented in this work are novel and carry significant impact for the community. The experimental demonstration of fundamental non-Hermitian theoretical results is both timely and important. Hence, I recommend it for publications.

Open Access This Peer Review File is licensed under a Creative Commons Attribution 4.0 International License, which permits use, sharing, adaptation, distribution and reproduction in any medium or format, as long as you give appropriate credit to the original author(s) and the source, provide a link to the Creative Commons license, and indicate if changes were made.
In cases where reviewers are anonymous, credit should be given to 'Anonymous Referee' and the source.
The images or other third party material in this Peer Review File are included in the article's Creative Commons license, unless indicated otherwise in a credit line to the material. If material is not included in the article's Creative Commons license and your intended use is not permitted by statutory regulation or exceeds the permitted use, you will need to obtain permission directly from the copyright holder.
To view a copy of this license, visit https://creativecommons.org/licenses/by/4.0/

Response Letter to Reviewers

We are grateful for the constructive comments on this manuscript (NCOMMS-25-80794) from all reviewers. In the text below, the comments from each reviewer are quoted in *blue italics*, and followed by our response. We have also revised the manuscript and the Supplemental Information accordingly, and these updates are highlighted in red in those files. In the text below, references to these updates are also highlighted in red. For the reviewers' convenience, key figures added to or discussed in the revised manuscript and Supplementary Information are reproduced in this response letter and are labeled as Fig. R1, Fig. R2, etc. Throughout this response letter, we refer to these reproduced figures using the R-numbering system.

Reviewer #1

Reviewer comments:

In this manuscript the authors present experimental demonstration of a nonlinear and non-Hermitian system which consists of coupled microwave resonators. In particular, they demonstrate nonlinearity-induced topological phase transition (with reversal of the skin mode localization): this is enabled due to a specially engineered nonlinear coupling which exists only along one direction. Apart from reversal of the skin mode localization, the authors also demonstrate harmonic generation produced by skin modes.

I find the manuscript to be very interesting (and timely) to the community, as it presents a convenient and robust platform for the study of the interplay between non-linearity and non-Hermiticity, in particular by enabling the non-reciprocal coupling to be nonlinear. Numerical analysis and experimental results are clear and convincing.

Response:

We are grateful to Reviewer #1 for the positive comments and the favorable recommendation for publication of this work in Nature Communications.

Reviewer comments:

I do have few minor comments, as follows. Large emphasis in the paper is given to the elaboration of the theoretical model and investigation of its properties, and this is good for the clarity of the paper. However, the most important part of this paper is that it shows that one can have nonlinear nonreciprocal couplings in a system of microwave resonators. I think

this should be elaborated in more details even in the main text. At present, the mechanism which enables it is somewhat hidden in the Supplementary Information, and I think it could be explained more clearly.

Response:

We thank the reviewer for this constructive suggestion and agree that the mechanism of the nonlinear nonreciprocal coupling should be elaborated in more detail in the main text. In the revised manuscript, we have added more description of the nonlinear nonreciprocal coupling on page 4, right column, which reads: "Then it is unidirectionally amplified and coupled to resonator 2 via the other lumped capacitor. The amplitude gain and phase shift introduced by the LNA are captured by the complex coefficient $\tilde{\kappa}_2$. At low input power, the LNA operates in the linear regime, and $\tilde{\kappa}_2$ remains effectively constant. As the input power increases, nonlinear gain compression occurs, where the fundamental amplification is reduced and waveform distortion emerges, leading to a power-dependent complex coupling coefficient. In this way, a nonlinear nonreciprocal coupling is realized in the present setup."

Reviewer comments:

Secondly (and related to this), the authors should discuss the possibility of extending their setup from 1D to 2D; I think this would be important to make the paper even more relevant.

Response:

We thank the reviewer for this insightful comment. In fact, the present four-arm H-shaped resonator design naturally generalizes to two-dimensional networks, as each arm can serve as an independent coupling channel, enabling planar connectivity. Related 2D implementations have already been demonstrated in linear regimes (see [Nature 555, 346 (2018)] and [Phys. Rev. Lett. 135, 206602 (2025)]), and we are indeed working on a 2D nonlinear realization. To emphasize the possibility of extending our setup to 2D, we have added the following discussions in the conclusion part of the main text: "Moreover, the current four-arm H-shaped resonator design naturally extends to two-dimensional networks, as each arm offers an independent coupling channel, facilitating planar connectivity. Hence, it would be highly desirable to extend our system to two dimensions, where novel physics emerging from the interplay among nonlinearity, non-Hermiticity, and band topology can be further explored."

Reviewer comments:

Lastly, it would be beneficial if the authors could comment on other limitations of the setup (e.g, maximum power/nonlinearity, noise etc.).

Response:

Following the reviewer's suggestion, we have expanded the discussion of the practical limitations of the setup. A brief summary has been incorporated into the main text on page 4, right column, which reads: "In practice, the experimentally accessible parameter window of the coupling is limited by component tolerances, amplifier nonlinear operating range, and measurement noise, which may induce resonator detuning and introduce additional effective gain or loss. However, by choosing high-quality LNA and conducting careful calibration, the desired parameter values can be reached."

In addition, a more detailed discussion has been added to the Supplementary Note 4. The added text reads as follows:

"Several practical limitations of the current setup should be noted. Firstly, variations in discrete components (particularly capacitors) introduce disorder in the coupling parameters. While moderate disorder does not qualitatively alter the observed phenomena, sufficiently large fluctuations may affect the nonlinearity-induced topological phase transitions. Secondly, the nonlinear response is governed by the characteristics of the RF amplifier. In the present implementation, the high-linearity MGA-53543 amplifier provides a controlled saturable response with measurable third-harmonic generation. Different amplifier types with narrower linear ranges may result in stronger harmonic content and a reduced controllable regime. Lastly, signal noise must be minimized to ensure system stability, especially at high input power. Otherwise, large power fluctuation or uncontrolled power growth can invalidate the theoretical modelling and even damage the sample."

Reviewer comments:

To conclude, I find the work presented here to be important and results to be excellent. My recommendation is that the manuscript should be published in Nature Communications.

Response:

We thank Reviewer #1 again for the constructive comments and positive remarks.

Reviewer #2

Reviewer comments:

In this manuscript, the Authors report an experimental study of the nonlinear non-Hermitian skin effect. While the topic may be of interest to a relatively specialized community (it is not actually a new one), the manuscript appears to me largely unreadable and unreproducible. Therefore, I cannot recommend it for publication. My main (although not all) reasons are summarized below.

Response:

We sincerely thank Reviewer #2 for the very careful reading of our manuscript and the comprehensive comments, which have helped us significantly improve the manuscript. We acknowledge that the initial submission contained insufficient clarification in several definitions and derivations, which may have affected its readability. In the revised version, we have substantially improved the presentation by explicitly defining all variables, providing detailed derivations where necessary, and clarifying the connection between theory and experiment. A detailed point-by-point response is provided below.

On the other hand, we believe that the topic of our work, i.e., nonlinear non-Hermitian topological physics, is new and timely, as also acknowledged by the other two reviewers. As we discussed in the introduction part of the main text, the study in this direction is just starting to bloom, and experimental works are still rare. Meanwhile, researchers from diverse directions are joining this direction, including those from condensed matter, quantum physics, photonics, and acoustics (see Refs. [56-65] in the main text). From this perspective, we think this direction can be considered as of broad interest. To further strengthen our work's novelty and significance, we have expanded our discussion to a nonlinear non-Hermitian SSH model, which shows a nonlinearity-induced simultaneous point-gap and line-gap phase transition that has never been studied before (see Fig. 5 in the revised manuscript). We hope these improvements on the technique issues and scientific novelty can resolve the reviewer's concerns.

Reviewer comments:

1. Equation (1) introduces creation and annihilation operators, but their physical meaning is not specified. It is unclear what these operators are meant to create or annihilate, whether they are bosonic or fermionic, and what commutation relations they satisfy. Moreover, their connection to the experimental setup is not explained. The manuscript does not

discuss the role of quantum effects or justify the use of a classical approximation. This lack of clarification makes it difficult to understand how the theoretical model relates to the experiment.

Response:

Our system describes classical electromagnetic wave propagation in a coupled resonator array. The operator notation used in Eq. (1) in the original manuscript follows the standard convention in classical coupled-mode theory, especially in electromagnetic systems, where creation and annihilation operators are bosonic and act on the complex field amplitudes of the resonant mode. However, from the reviewer's comment, we realize that the previous notation may lead the readers to take our system as a quantum one due to the use of creation and annihilation operators. To prevent potential confusion, we reformulated Eq. (1) in the main text as a dynamical coupled-mode equation without using quantum-like notations. The revised part is also given below:

"Consider a classical nonlinear Hatano-Nelson model as depicted in Fig. R1a, which generically describes an array of N coupled photonic resonators with linear reciprocal couplings and nonlinear nonreciprocal couplings. The system is governed by the coupled-mode equations:

$$i\frac{d\Psi_n}{dt} = f_0\Psi_n + t_{r,n}\Psi_{n-1} + t_{l,n}\Psi_{n+1}, \tag{R1}$$

where n is the site index, Ψ_n is the complex field amplitude at the n-th site, ..."

Reviewer comments:

2. The statement "Without loss of generality, we set the linear reciprocal coupling as unity" (before Eq. (2)) is questionable. In nonlinear systems, rescaling coupling coefficients is not generally innocuous: modifying the magnitude of these coefficients typically requires a corresponding rescaling of the field amplitudes, which changes physically relevant parameters such as effective nonlinearity. Therefore, setting this coefficient to unity does reduce generality, and the authors should justify why this simplification does not affect the validity of their conclusions.

Response:

We thank the reviewer for the concern regarding the rescaling of the coupling coefficient. Indeed, in nonlinear systems such as Kerr-type nonlinear systems, rescaling coupling coefficients may affect the relative nonlinearity. However, our system features a saturable

nonlinear coupling rather than a Kerr-type one. Since the nonreciprocal coupling strength depends on the amplifier response, it can be physically tuned by regulating the RF circuits to compensate for normalization. As a result, the simplification does not compromise the generality or physical validity of the model. To support our claim, we show in Fig. R1 the numerical results when the linear reciprocal coupling is set as $t_l = 2$. As can be seen, both the phase diagram and the eigen spectra agree well with the results given in Fig. 1 in the main text.

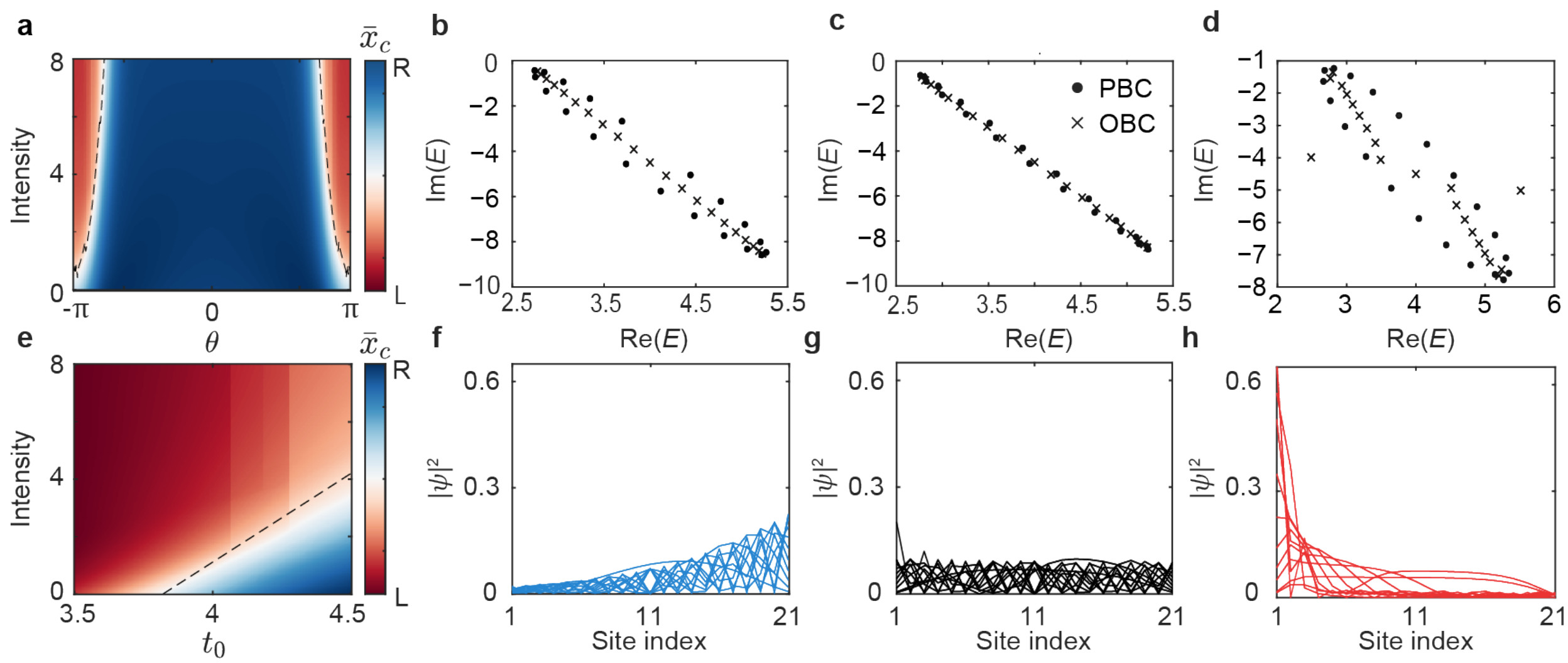

Fig. R1 Nonlinear Hatano-Nelson model with $t_l = 2$. **a**,**e** Plot of the average position of all OBC eigenvectors as functions of intensity I and θ (and t_0), with a fixed $t_0 = 4.1$ ($\theta = -0.9\pi$). The black dashed line denotes the point-gap closing points obtained from PBC spectra. **b-d** Plots of eigenvalues on the complex plane under PBC (dots) and OBC (crosses) for the three intensity values highlighted by the blue (b), black (c), and red (d) star markers in **a** and **e**. **f-h** The corresponding OBC eigenvectors for **b-d**. In all calculations, we take $f_0 = -4.5i$, $t_l = 2$, $t_\infty = 0.4$ and $t_c = 1$.

Reviewer comments:

3. In Eq. (2), the field intensities I_i are introduced, yet their relation to the creation and annihilation operators appearing in Eq. (1) is not explained. This link is crucial for the system to be regarded as nonlinear.

Response:

We thank the reviewer for the comment. In the revised manuscript, we have added the definition of field intensity after Eq. (2), which reads: "Here, $I_n = |\Psi_n|^2$ is the field intensity

at the n-th site."

Reviewer comments:

4. In the second paragraph on p. 3 the Authors introduce the eigenvalue problem $H\psi = E\psi$. ψ is not defined here. What is it?

Response:

We thank the reviewer for this question. Here, ψ denotes the vector of complex field amplitudes across all sites, i.e., $\psi = (\psi_1, \psi_2, \ldots, \psi_N)^T$. The eigenvalue problem is derived from the coupled mode equation (i.e., Eq. (1) in the revised manuscript) by taking the time-harmonic solution $\Psi(t) = \psi e^{-iEt}$. The corresponding Hamiltonian (under open boundary conditions) is given by

$$H = \begin{pmatrix} f_0 & t_{l,1} & 0 & \cdots & 0 \\ t_{r,1} & f_0 & t_{l,2} & \cdots & 0 \\ 0 & t_{r,2} & f_0 & \ddots & \vdots \\ \vdots & \vdots & \ddots & \ddots & t_{l,N-1} \\ 0 & 0 & \cdots & t_{r,N-1} & f_0 \end{pmatrix}. \tag{R2}$$

To enhance the clarity of this part, we have made the following revisions in the main text on page 3, left column: "we search for time-harmonic solutions of the form $\Psi_n(t) = \psi_n e^{-iEt}$ to Eq. (1). This leads to the nonlinear eigenproblem $H\psi = E\psi$, where E is the eigenvalue, $\psi = (\psi_1, \psi_2, \ldots, \psi_N)^T$ is the eigenvector, and H is the Hamiltonian containing the on-site and coupling terms (see Methods for more numerical details)."

Reviewer comments:

5. Formula (3) appears to be valid only for odd N. This restriction must be clearly stated. If the authors use this expression exclusively for the case $N = 21$, then writing it in a "general" form is somewhat misleading. In that case, it would be more appropriate to present the formula specifically for $N = 21$ rather than implying general validity that does not hold.

Response:

Eq. (3) in the main text also applies to systems with an even number of lattice sites. For

any number of lattice sites N, such a summation of a single eigenmode can be expressed as

$$\sum_{j=1}^{N}|\psi_j|^2\frac{(j-(N+1)/2)}{(N-1)/2}=\frac{1}{(N-1)/2}\left(|\psi_1|^2,|\psi_2|^2,\ldots,|\psi_{N-1}|^2,|\psi_N|^2\right)\begin{pmatrix}-\frac{N-1}{2}\\ -\frac{N-3}{2}\\ \vdots\\ \frac{N-3}{2}\\ \frac{N-1}{2}\end{pmatrix}, \quad \text{(R3)}$$

which is applicable to both even and odd numbers of lattice sites. For instance, in the case of $N = 20$, when the states are uniformly distributed across all sites, the summation $\sum_{j=1}^{20}(j-10.5)$ yields zero, leading to $x_c = 0$, which corresponds to a uniformly distributed state profile.

To make Eq. (3) more accessible, we have simplified the formula as follows:

$$\bar{x}_c=\langle\sum_{j=1}^{N}|\psi_j|^2\frac{(j-(N+1)/2)}{(N-1)/2}\rangle, \quad \text{(R4)}$$

where ψ_j is the amplitude of the eigenmode at site j, $\langle\cdot\rangle$ denotes the average over all eigenmodes.

Reviewer comments:

6. A more serious issue concerns the mismatch between experiment and numerics. The experiment is performed with $N = 11$ sites (Fig. 3a), yet theoretical calculations are carried out for $N = 21$ sites. This choice does not provide additional generality; instead, it creates a discrepancy between the numerical and experimental results.

Response:

The number of sites in the theoretical model does not substantially affect the core findings (i.e., the nonlinearity-induced topological phase transition). In our calculations, we take $N = 21$ mainly to make the PBC eigenvalue spectrum more visually distinct and the phase transition process clearer. In the main text, Figure 3 also presents calculation results with $N = 11$ (Fig. 3e-h), which align well with the experimental results and agree qualitatively with the calculation results with $N = 21$. To emphasize this point, we have added the following remark in the main text on page 5, left column: "Note that we use fewer resonators ($N = 11$) to facilitate the experimental implementation, and the localization reversal phenomenon persists under the size change of the system."

Reviewer comments:

7. In the same paragraph, when discussing the threshold there appear formulas that follow from nowhere. Where do they come from?

Response:

We thank the reviewer for the comment. The threshold compares the effective rightward and leftward coupling strengths at different intensity levels, where $|\tilde{\kappa}_{2,n}| \approx t_0$ at low intensity and $|\tilde{\kappa}_{2,n}| \approx t_\infty$ at high intensity. To avoid abrupt phrasing and to present the parameter conditions more clearly, we have made the following revisions in the manuscript on page 3, left column: "A threshold, given by $|\kappa_1 + \tilde{\kappa}_{2,n}(I_n \to 0)| > \kappa_1$, is required, such that rightward couplings are dominant at zero intensity. In addition, the high-intensity coupling needs to satisfy $|\kappa_1 + \tilde{\kappa}_{2,n}(I_n \to \infty)| < \kappa_1$ to make the reversal happen."

Reviewer comments:

8. In general, the transmission problem and the eigenmode problem are distinct (although related). In Fig. 2(d), the authors present transmission coefficients, yet these coefficients are neither defined nor discussed in the main text.

Response:

Indeed, the transmission and the eigenmode problems are related but not identical. In the microwave experiment, the transmission coefficients provide the primary experimentally accessible observables and serve as indirect probes of the underlying eigenmode properties. In Supplementary Note 2, we derive the transmission coefficients from the effective Hamiltonian via the Green's function formalism, which establishes their connection to the system's tight-binding parameters. For clarity, we have added the following sentence in the main text on page 5, left column, to emphasize the connection between the transmission spectra and tight-binding parameters: "Using a Green's function method, we can establish the relationship between the transmission spectra and the tight-binding coefficients, allowing us to extract model parameters from experimental transmission data (see Supplementary Note 2)."

Reviewer comments:

9. I do not understand the meaning of the color plots in Figures 3b to 3d. What does f represent? Usually, frequency is a characteristic of a mode rather than a site. Without clear explanations, the figure appears meaningless. In the supplementary information the authors say that f "is the frequency of the driving signal". But there is no driving term in the Hamiltonian (1) nor f can be an eigenvalue.

Response:

The color plots in Fig. 3b-d represent the measured transmission amplitude at each site in the frequency window of our interest. As the reviewer has noted in Comment 8, "the transmission problem and the eigenmode problem are distinct (although related)." In our experiment, the reversal of NHSE and harmonic generation is characterized through transmission measurements, as commonly used in microwave experiments. The parameter f in Fig. 3b-d denotes the frequency of the external signal used in transmission measurement. Each color plot shows the field amplitude distribution across the array at a series of driving frequencies. Eq. (1) provides a general theoretical model for analyzing eigenmodes, independent of specific excitation, while the driving frequency f is introduced experimentally as a probing parameter. We note that such a characterization process is commonly adopted in diverse classical and quantum experiments: the system is theoretically analyzed using eigen-equations (e.g., dispersion, eigenmode profile and band topology) and experimentally probed by exciting at selected frequencies and positions.

Reviewer comments:

10. Turning now to the supplementary information, where does (S2) come from? What is the equation for the Green's function? The authors neither provide references where this formula can be found nor derive it.

Response:

Eq. (S2) in the Supplementary Information is the standard frequency-domain Green's function widely used in classical-wave physics, quantum physics and condensed matter physics. A brief derivation based on the coupled mode theory is given below (also see Chapter 2 of Ref. [1]).

For a time-harmonic excitation, the complex amplitudes of the resonators $\psi(t)$ satisfy the temporal coupled-mode equation:

$$\frac{i}{2\pi}\frac{d}{dt}\psi(t) = H_{\text{eff}}\psi(t) + F(t), \tag{R5}$$

where $\psi(t)$ is the vector of resonator amplitudes, H_{eff} is the effective Hamiltonian given in Eq. (4) of the main text which governs the classical wave dynamics in the non-Hermitian framework, and $F(t)$ represents the external driving term applied through the input port. Taking the Fourier transform $\psi(f) = \int_{-\infty}^{\infty} \psi(t)e^{i2\pi ft}dt$, we obtain the algebraic relation,

$$[fI - H_{\text{eff}}]\,\psi(f) = F(f), \tag{R6}$$

where f denotes the driving frequency and I is the identity matrix of the same dimension as H_{eff}. The Green's function is therefore defined as the resolvent of the effective Hamiltonian,

$$G(f) = [fI - H_{\text{eff}}]^{-1}. \tag{R7}$$

Physically, $G(f)$ describes the linear response of the system in frequency space.

To address the reviewer's comment, we have added two references (i.e., Refs. [1, 2]) when giving Eq. (S2) in the Supplementary Note 2.

Reviewer comments:

11. In the main text the Authors use $\hat{H}$ while in (S2) they use H. What is the relation between these two quantities?

Response:

We apologize for this inconsistency in the notation. They are both Hamiltonians. In the revised version, we use the notation H throughout the work. Specifically, the H in the eigen equation $H\psi = E\psi$ on page 3 of the main text refers to the lattice Hamiltonian under either open or periodic boundary conditions; while the H_{eff} in Eq. (4) of the main text and Eq. (S2) of the Supplementary Information is the Hamiltonian for two coupled resonators with dissipation taken into account.

Reviewer comments:

12. Where do formulas (S3) - (S5) come from? How can they be computed?

Response:

The former Eqs. (S3)-(S5) are derived in the Supplementary Information using the Green's function formalism and the input-output relation $S = I - W^{\dagger}GW$. The resulting transmission coefficients are fully consistent with those obtained from the standard coupled-mode theory approach. We have added a short clarification in the Supplementary Note 2. Following the Green's function, the derivation of the transmission coefficients is as follows:

"Consider two input/output channels connected to the two SMA ports of the system as shown in Fig. 2a in the main text. Under the Mahaux-Weidenmüller formula [3], we can obtain the transmission coefficients as

$$S(f) = I - W^{\dagger}G(f)W, \tag{R8}$$

where W is the channel–mode coupling matrix. Assuming symmetric external coupling rate

γ_1 and that port 1 (port 2) couples only to resonator 1 (resonator 2), we take

$$W = \sqrt{2\gamma_1} \begin{pmatrix} 1 & 0 \\ 0 & 1 \end{pmatrix}, \tag{R9}$$

which leads to scattering coefficients upon substituting the matrix elements of $G(f)$."

Reviewer comments:

13. In (S7) the Authors say "Assuming a time dependence of...". Of what? Furthermore, what is the relation between the frequencies ω and f?

Response:

Thank you for your question. Here, we assume the input signal to be a continuous wave with a time dependence of $e^{-i\omega t}$. The angular frequency ω is related to the linear frequency f by $\omega = 2\pi f$. To avoid confusion, we have unified the notation in the revised version by using the linear frequency f throughout the text.

Reviewer comments:

14. It is not explained how Y_{21} appears in (S7) and how it is related to the rest of the parameters used in the main text. Also why are Y_{ii} small?

Response:

The admittance matrix originates from the transmission characteristics of the RF amplifier, which enhance the forward admittance Y_{21} while suppressing the backward admittance Y_{12}. From an engineering perspective, the diagonal terms Y_{ii} are intentionally suppressed to minimize self-coupling, which could introduce additional loss and parasitic effects.

Reviewer comments:

15. What are v_0 and v_i in (S13)? What are a_i? All these quantities are not defined.

Response:

Thank you for raising this issue. We have added the corresponding definitions in the revised Supplementary Note 5, which reads: "where v_i and v_o denote the time-domain input and output signals, respectively. The response is characterized by the following coefficients: a_0 is the DC offset component, a_1 is the fundamental linear gain, and a_i $(i > 1)$ are the nonlinear higher-order harmonic coefficients."

Reviewer comments:

16. I cannot understand this phrase "As symmetrical saturation suppresses the even harmonics," What does "saturation" mean here?

Response:

The term “saturation” refers to the nonlinear compression regime of the amplifier output when the input signal amplitude exceeds the linear operating range, so that the output no longer scales linearly with the input and harmonic components are generated. In the time-domain signal, this saturation manifests as the flattened patterns around the peaks and dips of the original sinusoidal curve, as illustrated in Fig. R2a,b. In particular, Fig. R2a and Fig. R2b show the symmetrical and asymmetrical saturation patterns, respectively.

To reveal the difference between symmetrical and asymmetrical saturations, we consider a sinusoidal input $v_i = A\cos(2\pi f t)$ and express the nonlinear response as a Taylor expansion,

$$v_o = a_0 + a_1 v_i + a_2 v_i^2 + a_3 v_i^3 + a_4 v_i^4 + a_5 v_i^5 + \cdots . \tag{R10}$$

For symmetrical saturation, the effective transfer characteristic is odd-symmetric around the operating point, namely,

$$v_o(-v_i) = -v_o(v_i). \tag{R11}$$

Substituting $-v_i$ into Eq. (R10) and comparing the resulting expression term by term with $-v_o(v_i)$, one finds that the constant term and all even-order coefficients must vanish. That is,

$$a_0 = 0, \qquad a_{2n} = 0 \quad (n = 1, 2, 3, \dots). \tag{R12}$$

Therefore, under symmetrical saturation, the output contains only the fundamental and odd harmonics, while asymmetrical saturation gives rise to both odd and even harmonics. The corresponding frequency-domain responses are shown in Fig. R2c,d, which agree well with our theoretical analysis. Note that here the parameters are chosen as representative theoretical values only for illustrative purposes, rather than for quantitative fitting of the experimental data.

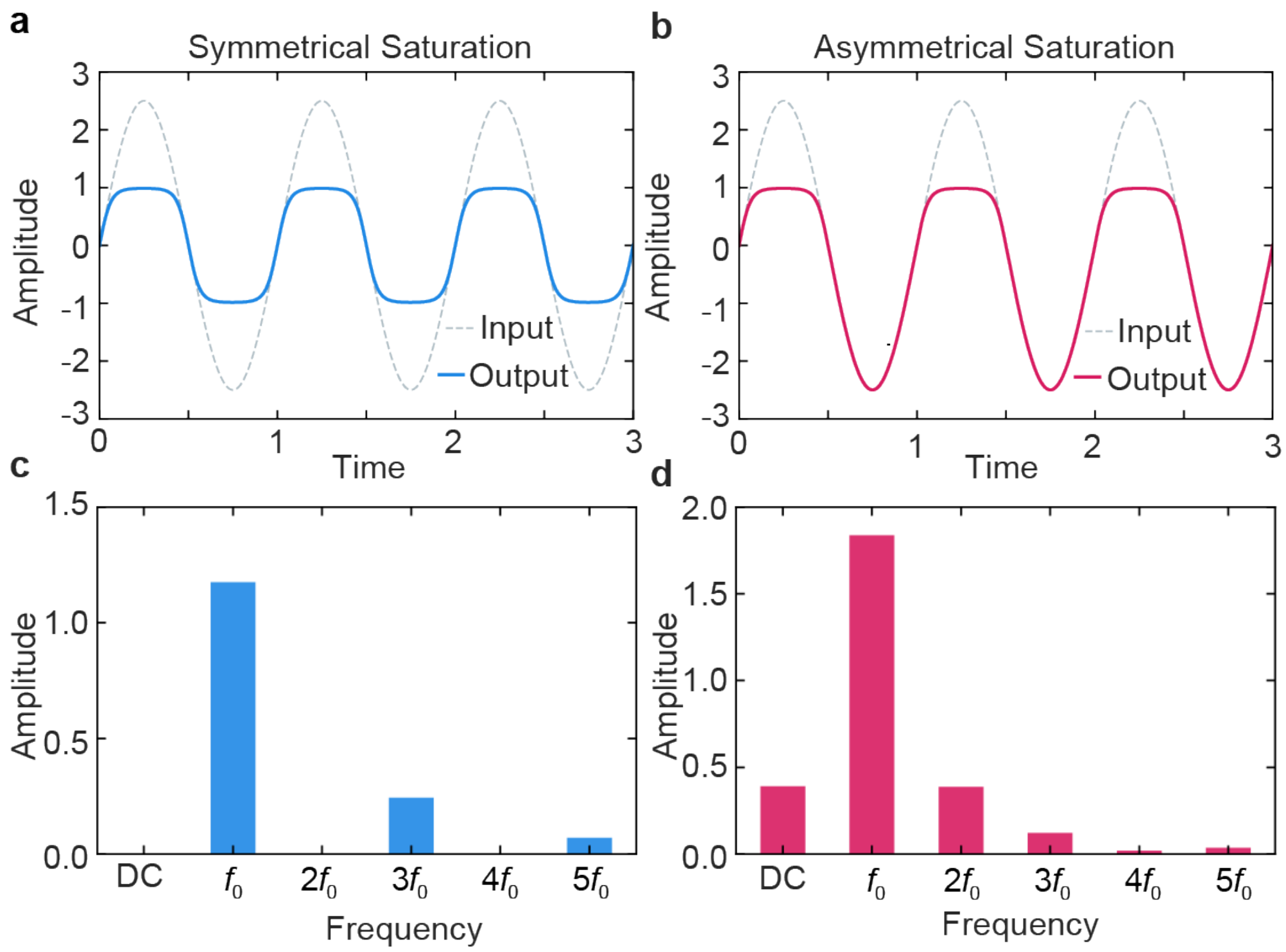

Fig. R2 Time-domain waveforms for **a** symmetrical saturation and **b** asymmetrical saturation, with the corresponding frequency-domain spectra shown in **c** and **d**, respectively.

Reviewer comments:

17. The whole discussion in Supplementary note 5 might be meaningful only if A is small enough. What does "low input power" mean in mathematical terms?

Response:

Firstly, in RF systems, the input signal is usually maintained in a low-power regime, so A remains sufficiently small that the cubic term represents a correction rather than overwhelming the fundamental response. Furthermore, in mathematical terms, "low input power" corresponds to the regime $|a_1 A| \gg \left|\frac{3a_3A^3}{4}\right|$, so that the fundamental component remains approximately linear.

We would like to mention that the former Supplementary Note 5 is intended for an intuitive understanding of the harmonic generation phenomenon rather than a mathematical framework. To remind the readers of this, we have added the following sentence at the beginning of this section: "In this section, we present a simple model to understand the harmonic generation mechanism in our system intuitively."

Reviewer comments:

18. I do not understand why the authors use round brackets (..) in some places and square brackets [..] in others. Are these parts written by different co-authors and none in the team could unify notations, or is there a more significant reason for this distinction?

Response:

We thank the reviewer for the careful observation. In the revised manuscript, we have clarified and unified the notation scheme. Square brackets are now consistently used for matrix expressions and operator structures, while round brackets are reserved for function arguments and algebraic grouping. The notation has been unified throughout the manuscript.

Reviewer comments:

To conclude, hindered by all the problems listed above, I could not find any discussion of new physics that this work might reveal.

Response:

We sincerely appreciate Reviewer #2's efforts in reviewing our work, and hope our detailed responses and extensive revisions can resolve the reviewer's concerns.

Reviewer #3

Reviewer comments:

The authors report an experimental observation of a nonlinearity-induced reversal of the non-Hermitian skin effect (NHSE) in a microwave metamaterial platform, accompanied by harmonic generation. The work addresses an interesting and timely direction at the intersection of nonlinearity and non-Hermitian topology. The experimental platform is well developed, and the results are potentially impactful. However, in its current form, the manuscript requires substantial clarification and strengthening before it can be considered for publication.

Below, I provide major and minor comments that I believe will help improve the clarity, rigor, and interpretability of the work.

Response:

We thank Reviewer #3 for the positive assessment and the constructive suggestions, which indeed helped us improve our manuscript significantly. Below, we provide point-to-point responses to the reviewer's comments.

Reviewer comments:

The introduction motivates nonlinear non-Hermitian physics, but the manuscript should more clearly state what is fundamentally new about localization reversal compared to previously known nonlinear effects (e.g., self-induced topological transitions, nonlinear spectral shifts).

Response:

We thank the reviewer for this suggestion. The fundamental difference between the localization reversal in our work and previously known nonlinear effects is that the localization reversal phenomenon is a result of the point-gap topological phase transition unique to non-Hermitian systems. We have added the following statement in the revised introduction to emphasize the novelty of the localization reversal phenomenon: "These findings extend the study of nonlinear topological phenomena to the point-gap regime."

Reviewer comments:

1) The manuscript states that a nonlinearity-induced point-gap topological transition underlies the observed localization reversal. However, the physical origin and mathematical structure of this nonlinear effect remain under-specified.

Response:

We thank the reviewer for this important comment. In the revised manuscript, we have clarified both the physical origin and the mathematical structure of the nonlinear effect. In brief, the nonlinearity arises from the saturation of the RF amplifiers, which modifies the effective nonreciprocal coupling strength rather than inducing an onsite Kerr-type frequency shift. The intensity-dependent coupling enters the effective Hamiltonian self-consistently and drives a change in the point-gap topology, which we now quantify explicitly through the winding number calculation. The detailed mechanism, topological characterization, and further implications are addressed below.

Reviewer comments:

a) Please clarify what specific nonlinear mechanism is active in the circuit: is it effectively Kerr-type, saturable, gain-modulating, or coupling-modifying?

Response:

We thank the reviewer for this insightful question. The nonlinear mechanism in our system arises mainly from saturable nonlinearity and coupling-modifying. The nonreciprocal couplings in this system are implemented using RF amplifiers, whose gain saturates at high input intensity, thereby modifying the effective coupling strength and giving rise to nonlinear behavior. Specifically, the physical origin of the nonlinearity in our system is the maximum amplitude limit of the physical amplifiers. When the input microwave signal is weak, the amplifier operates in the linear regime, outputting a smooth sine wave. However, when the input power exceeds this linear range, the amplifier reaches its physical output capacity limit. Consequently, the gain of the nonreciprocal structure decreases, which dynamically compresses the nonreciprocal coupling coefficient $\tilde{\kappa}_2$. Furthermore, this saturation process distorts the time-domain waveform, directly resulting in the generation of higher-order harmonics. We have incorporated detailed discussions into the revised Supplementary Information. Specifically, in Supplementary Note 5.1, we have comprehensively refined the theoretical derivation of the saturable nonlinearity. While in Supplementary Note 5.2, we have explicitly demonstrated the intensity-dependent gain compression and the corresponding waveform distortion in the time domain.

Reviewer comments:

b) Can the authors derive or present a minimal effective Hamiltonian showing how nonlinearity drives a topological transition?

Response:

The topological phase transition cannot be captured by a minimal effective Hamiltonian for two reasons. Firstly, the point-gap topological phase transition is accompanied by a point gap closing that does not occur locally in the k-space. Hence, the typical procedure in the line gap case to obtain a minimal effective Hamiltonian (i.e., expanding around a k point), like a Dirac Hamiltonian with its mass flipped across the transition, cannot be applied to the point gap case. Secondly, for the systems with nonlinear couplings, the k-space Hamiltonian is not well-defined because the site intensities and the coupling strengths are nonuniform in space.

To more rigorously characterize and demonstrate the topological phase transition in our system, we have introduced and employed a method to calculate the winding number based on the real-space PBC Hamiltonian. We will present the computational details and results of this method in the response to the following question, which clearly delineates the topological phase transition as a function of nonlinear intensity.

Reviewer comments:

c) The connection between nonlinearity and point-gap topology should be more explicit. For example: How do the winding number or point-gap invariants evolve with pump intensity? Is the reversal strictly tied to a change in a topological index, or can it occur via a more conventional nonlinear redistribution of gain/loss?

Response:

We thank Reviewer #3 for the insightful comment. Indeed, the localization reversal can be tied to the change in the point-gap winding number. Following the method in Ref. [4] (originally proposed for calculating the winding number in disordered systems), we have calculated the winding number for the nonlinear PBC Hamiltonian. Specifically, a magnetic flux Φ is introduced into the PBC structure, which is implemented by multiplying the rightward and leftward coupling terms by phase factors $e^{-i\frac{\Phi}{N}}$ and $e^{i\frac{\Phi}{N}}$, respectively. This yields the transformed Hamiltonian $H(\Phi)$. Based on this, the winding number is defined as

$$w \equiv \int_0^{2\pi} \frac{d\Phi}{2\pi i} \partial_\Phi \ln \det(H(\Phi) - E_B)$$

Here, E_B is the reference point. The winding number w is calculated as the number of times the complex energy spectrum winds around E_B as the magnetic flux Φ varies from 0 to 2π. The corresponding calculation results are presented in Fig. R3. It should be emphasized

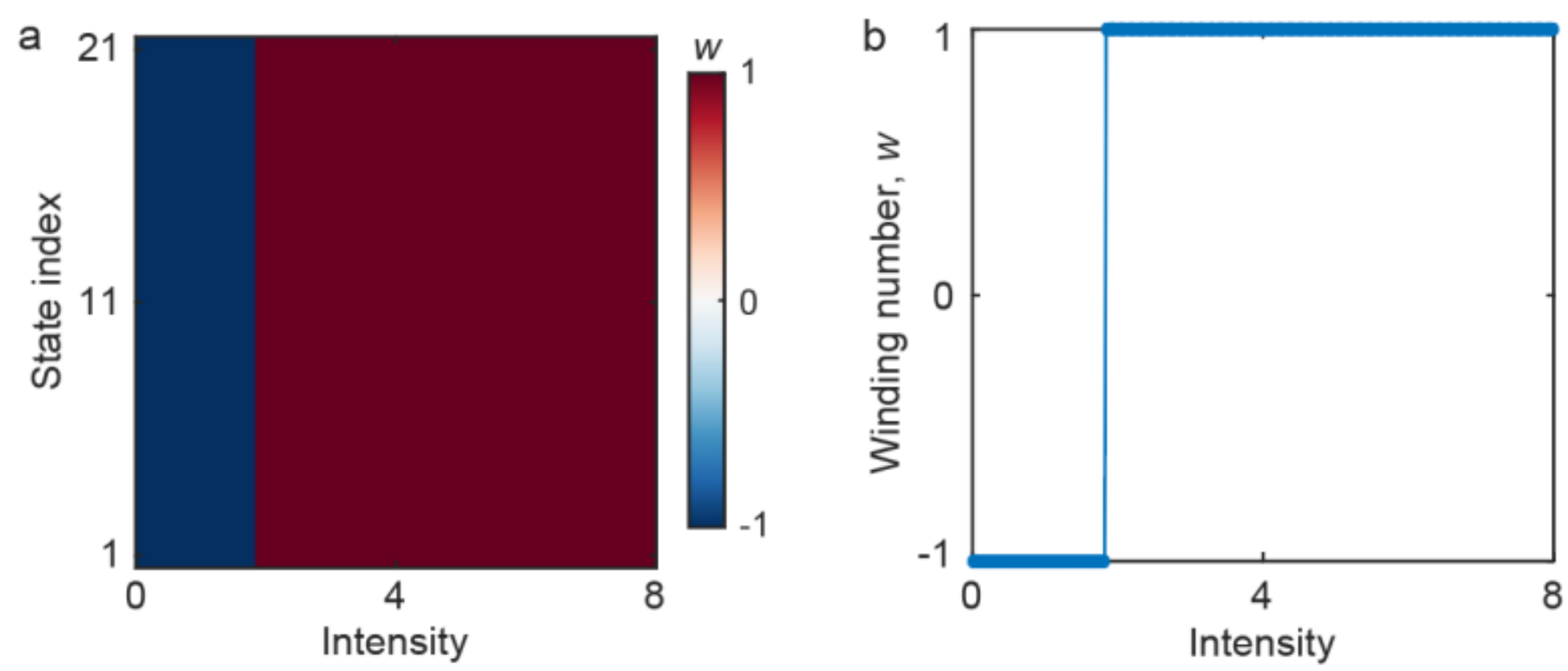

Fig. R3 **a** Winding number of the PBC Hamiltonian for each eigenvector as a function of total intensity. **b** Average winding number across all states at each intensity in panel (a). Parameters are consistent with those in Fig. 1 of the main text.

that in nonlinear systems, multiple steady states may exist for a given intensity. As shown in Fig. 1d-f in the main text, each PBC eigenvalue corresponds to a PBC Hamiltonian. In Fig. R3a, we have calculated the winding numbers for all PBC Hamiltonians at each intensity, and the results demonstrate excellent consistency. Furthermore, Fig. R3b shows the evolution of w with intensity in this nonlinear system, where the phase transition occurs at an intensity of 1.82. This is in good agreement with the PBC phase transition intensity of 1.72 shown in Fig. 1c of the main text.

In the Supplementary Note 1, we have added the following content:

"In this section, we introduce a topological index (i.e., the winding number) to characterize the point-gap topological phase transition and the localization reversal in the nonlinear Hatano-Nelson model. To this end, we introduce a magnetic flux Φ in the PBC Hamiltonian via multiplying the rightward and leftward coupling terms by the phase factors $e^{-i\frac{\Phi}{N}}$ and $e^{i\frac{\Phi}{N}}$, respectively, where N is the number of lattice sites [4]. This allows us to define the winding number of the system as:

$$w \equiv \int_0^{2\pi} \frac{d\Phi}{2\pi i} \partial_\Phi \ln \det \left(H(\Phi) - E_B\right), \tag{R13}$$

where E_B is the reference point. Here, the intensity-dependent coupling terms in the PBC Hamiltonian are obtained according to the intensity distribution of a certain PBC eigenmode. We note that the eigenmodes with the same total intensity yield the same winding number. As shown in Fig. S1, the winding number of the PBC Hamiltonian undergoes a transition

from -1 to 1 at an intensity around 1.82, corresponding to a change in the NHSE from right-sided to left-sided localization. This result is consistent with the findings presented in Fig. 1 in the main text."

Reviewer comments:

d) Fig 1 (f) the OBC spectrum has a plateau along the real line. Does it signify something?

Response:

The plateau observed in the OBC eigenvalue spectrum is an interesting feature of the nonlinear system. To visualize this phenomenon more clearly, we used color to distinguish the eigenvalues and their corresponding eigenmodes. The results distinctly reveal that the eigenmodes can be categorized into three groups based on their spatial localization: strongly localized modes (marked in red), localized modes (marked in purple), and poorly localized modes (marked in yellow), as plotted in Fig. R4. The modes along the plateau are still skin modes, but have very strong localization. Notably, two of them lie outside the PBC circle (which are missing in the original version due to numerical resolution). We speculate that this spectrum deformation comes from the fact that each nonlinear eigenmode corresponds to a different Hamiltonian due to the different spatial distribution. Hence, the OBC spectrum does not appear like the linear one. However, as this feature does not contradict our main story (i.e., the skin effect reversal), we feel it is better to leave this interesting aspect for future study.

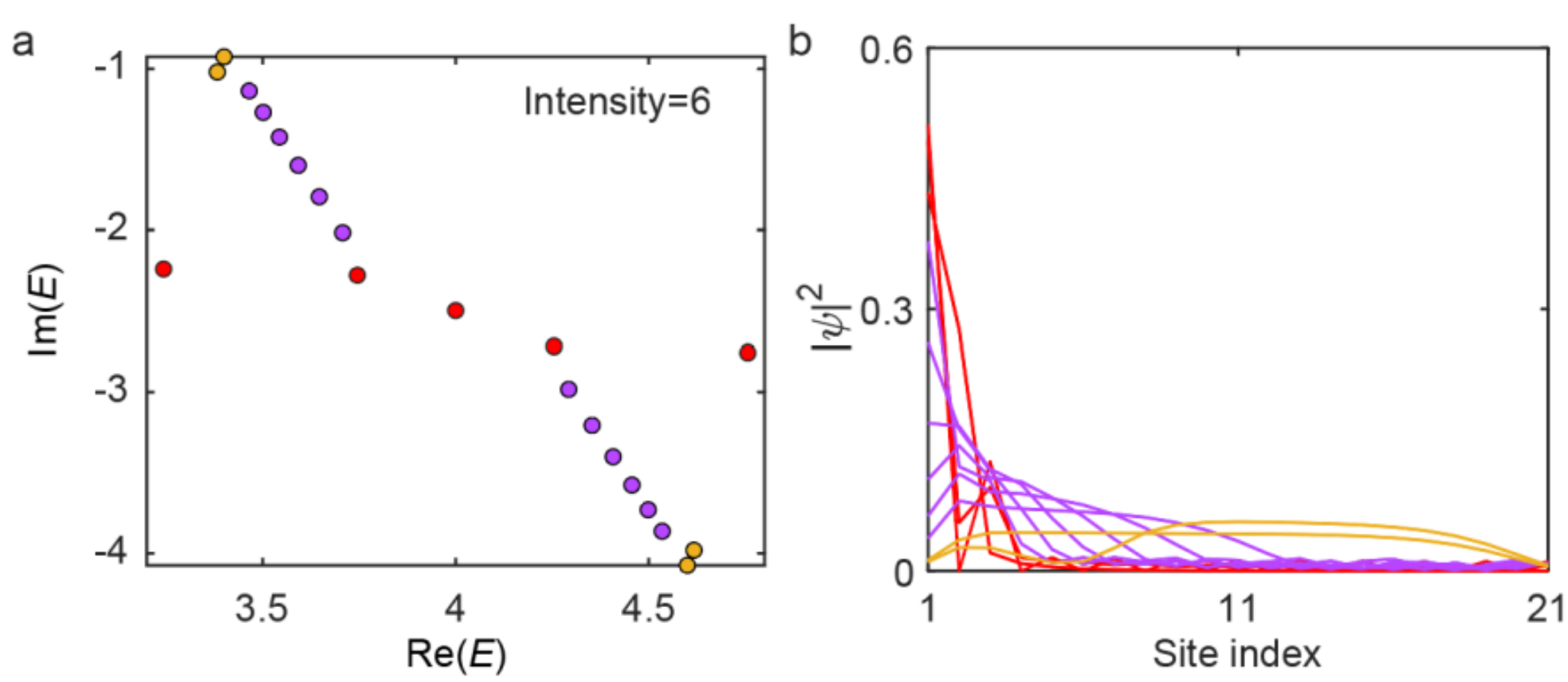

Fig. R4 Eigenvalues **a** and eigenvectors **b** of the OBC system at intensity = 6.

Reviewer comments:

e) It would be interesting to know, when there are both topological modes and skin modes (like in non-reciprocal SSH model), how the localization reversal phenomena would work?

Response:

We thank the reviewer for raising this interesting question. Indeed, the nonreciprocal SSH model, which has features that are absent in the Hatano-Nelson model (e.g., line-gap topology and breakdown of conventional bulk-boundary correspondence), is a fascinating system worthy of in-depth investigation.

In the revised version, we consider a nonlinear non-Hermitian SSH model that incorporates both nonlinearity and explicit nonreciprocal coupling, which is illustrated in Fig. R5a. The initial conditions are set as $|t_r| > |t_l|$ and $|t_r(I_n = 0)|\,|t_l| > |t_w|^2$ to ensure a right-localized NHSE and a topologically trivial phase at the outset. As the site intensity I_n increases, the value of $|t_r(I_n)|$ decreases, leading to a reversal of the NHSE direction (point-gap phase transition) and the emergence of topological edge modes (line-gap phase transition) in the system (Fig. R5b-h). In other words, this system features both types of topological phase transition driven by nonlinearity. Furthermore, the induced topological edge modes are reshaped by the NHSE and are distributed over the bulk, consistent with previous findings in linear systems (e.g., [Nature 608, 50–55 (2022)]).

We have conducted new calculations and experiments on this nonlinear non-Hermitian SSH model. A new section ("Nonlinear nonreciprocal SSH model") and a new figure (Fig. 5) have been added to the main text. The corresponding contents are also provided below for the reviewer's convenience:

"Finally, we extend our discussion to a nonlinear nonreciprocal SSH, whose linear version exhibits a series of novel phenomena from the interplay between point-gap and line-gap topologies, such as the breakdown of bulk-boundary correspondence and morphing of topological edge modes [5, 6]. As shown in Fig. R5a, a rightward nonlinear coupling t_r (following Eq. (2)) is introduced to the intracell coupling. All other couplings remain linear. The eigenvalues and eigenmodes as functions of total intensity are plotted in Fig. R5b–f for a finite chain under OBC. With initial parameter conditions $|t_r| \cdot |t_l| > |t_w|^2$ and $|t_r| > |t_l|$, the system is topologically trivial at low intensities, and the skin modes are localized around the right end (Fig. R5c,e). As the total intensity increases, the real parts of the eigenvalues of two modes gradually approach each other, corresponding to a line-gap phase transition. However, due to inevitable mode coupling in finite-length chains, these two modes are not

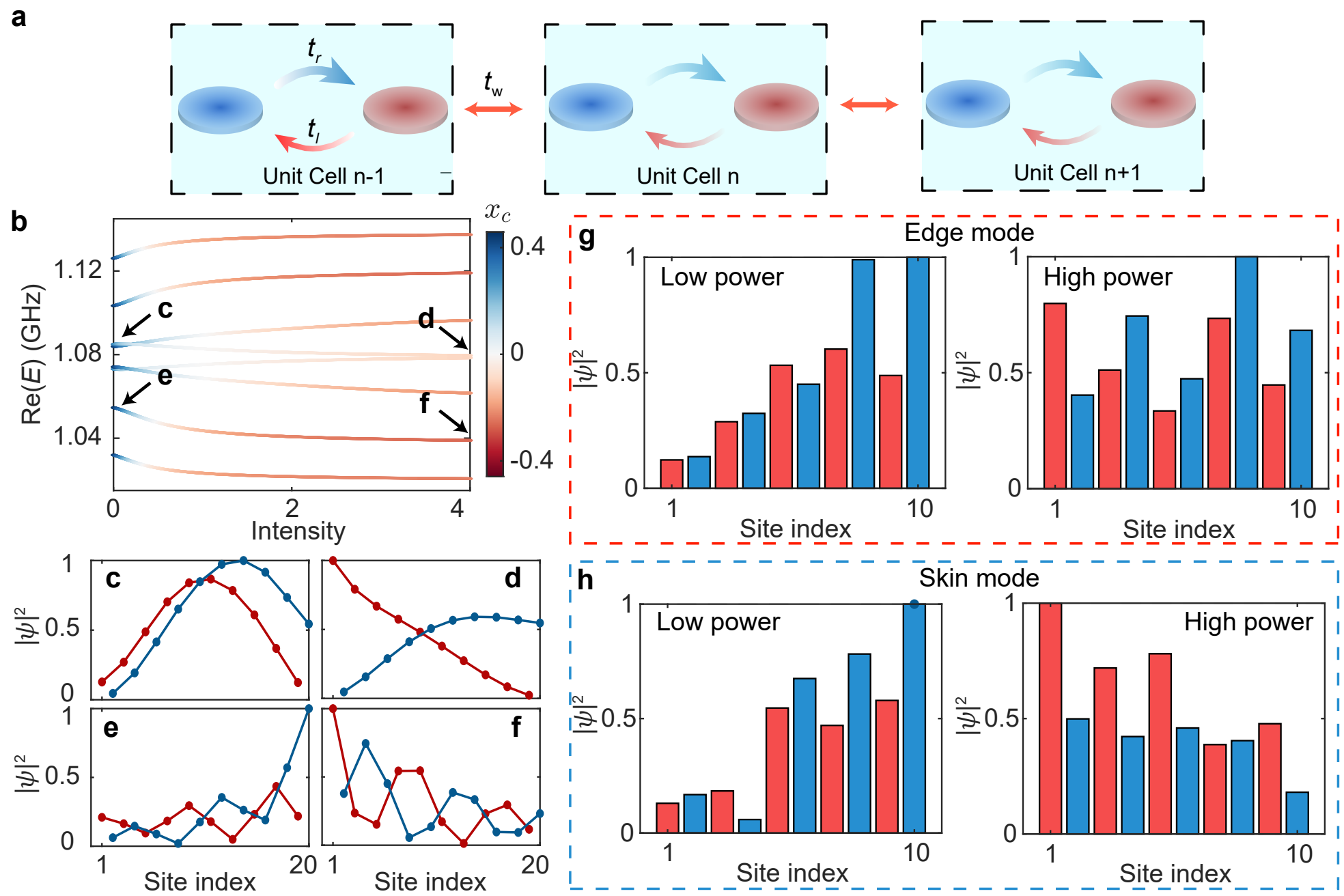

Fig. R5 Nonlinear nonreciprocal SSH model. a Schematic of the nonlinear SSH model. **b** Evolution of the real part of eigenvalues with respect to the total intensity I. **c-f** The evolution of the eigenvectors, with their corresponding eigenvalues and intensities labeled in **b**. Red and blue correspond to the odd and even sites, respectively. **g**, **h** The experimentally measured field distributions are obtained by exciting the system with an input field whose frequency and phase correspond to the edge mode (g), and the skin mode (h). The parameters are $t_l = t_w = 66$ MHz, $t_0 = 98.9$ MHz, $t_\infty = 32$ MHz, $\theta = -2.13$, and $t_c = 0.022$.

exactly degenerate [7]. Meanwhile, a point-gap phase transition also occurs, as can be seen from the blue-to-red color change in Fig. R5b and the eigenmodes plots in Fig. R5e,f. To further distinguish between edge modes and skin modes, we label the odd and even lattice sites with different colors. When the coupling strengths satisfy $|\ln(|t_r|/|t_l|)| < 2$, the edge modes exhibit opposite localization directions on odd and even sites, whereas the skin modes do not possess this property [8]. In our model, the nonlinear coupling strength ensures that this condition holds for all sites under arbitrary system intensities. As shown in Fig. R5d,f, at high intensities, the edge modes exhibit opposite localization directions on odd and even sites, while the skin modes localize in the same direction. This confirms the emergence of edge modes. In the experiment, we clearly observe that, as the input power increased, the field distribution on the odd and even sites at the frequency corresponding to the edge mode changes from same-side localization to opposite-side localization (Fig. R5g), accompanied

by a reversal of the NHSE (Fig. R5h). This indicates the simultaneous realization of both point-gap and line-gap phase transitions in the nonlinear SSH model."

In addition, we have added the following sentences in the abstract and introduction parts accordingly. In the abstract, we have added the sentence: "Furthermore, we extend our setup to build a nonlinear non-Hermitian Su–Schrieffer–Heeger model with both nonlinearity-induced point-gap and line-gap phase transitions." In the introduction (page 2, right column), we have added the sentence: "Moreover, we construct a nonlinear non-Hermitian Su–Schrieffer–Heeger (SSH) model [9] and discover that the point-gap and line-gap topology can be controlled by nonlinearity simultaneously."

Reviewer comments:

2) The reported reversal of skin localization is the central experimental signature, but alternative explanations must be addressed.

Response:

We thank the reviewer for raising this important concern. We agree that alternative explanations for the observed localization reversal must be carefully examined. In the revised manuscript, we have addressed this issue from both theoretical and experimental perspectives, including uniform pumping conditions, disorder robustness analysis, and quantitative characterization of the skin depth. The detailed clarifications are provided below.

Reviewer comments:

a) Could pump-induced gain/loss redistribution mimic a localization reversal even without a topological transition?

Response:

We thank the reviewer for this question. In fact, the issue has been taken into consideration both theoretically and experimentally. In the theoretical model, we assume a smooth and spatially uniform pumping process, independent of the injection point. In the experiment, we evenly distribute the input power into 11 resonators by a power splitter, which minimizes the effect of pump-induced gain/loss redistribution. The single-port excitation is used only in the harmonic-generation experiment, and this choice is made intentionally. We emphasize that the localization behavior at the fundamental frequency under single-port excitation is consistent with that obtained under multi-port excitation. As clarified in the main text: "The single-port excitation scheme is adopted intentionally, because if

multiple ports are driven simultaneously, each excitation channel may introduce weak parasitic higher-harmonic components from the source and feeding network, thereby leading to unwanted harmonic contributions in the measured signal."

Reviewer comments:

b) How robust is the effect to structural disorder, component tolerances, or asymmetry in the circuit?

Response:

We thank the reviewer for this important comment regarding the robustness of the NHSE reversal. Our calculations reveal that the reversal of the NHSE in this system exhibits remarkable robustness. Specifically, the reversal persists even when the reciprocal coupling is subject to disorder with a strength up to 1.74 times its value, or when the coupling phase contains a disorder amplitude up to 0.91π. This strong robustness protects the experimental observations against the inevitable fabrication imperfections in practical devices.

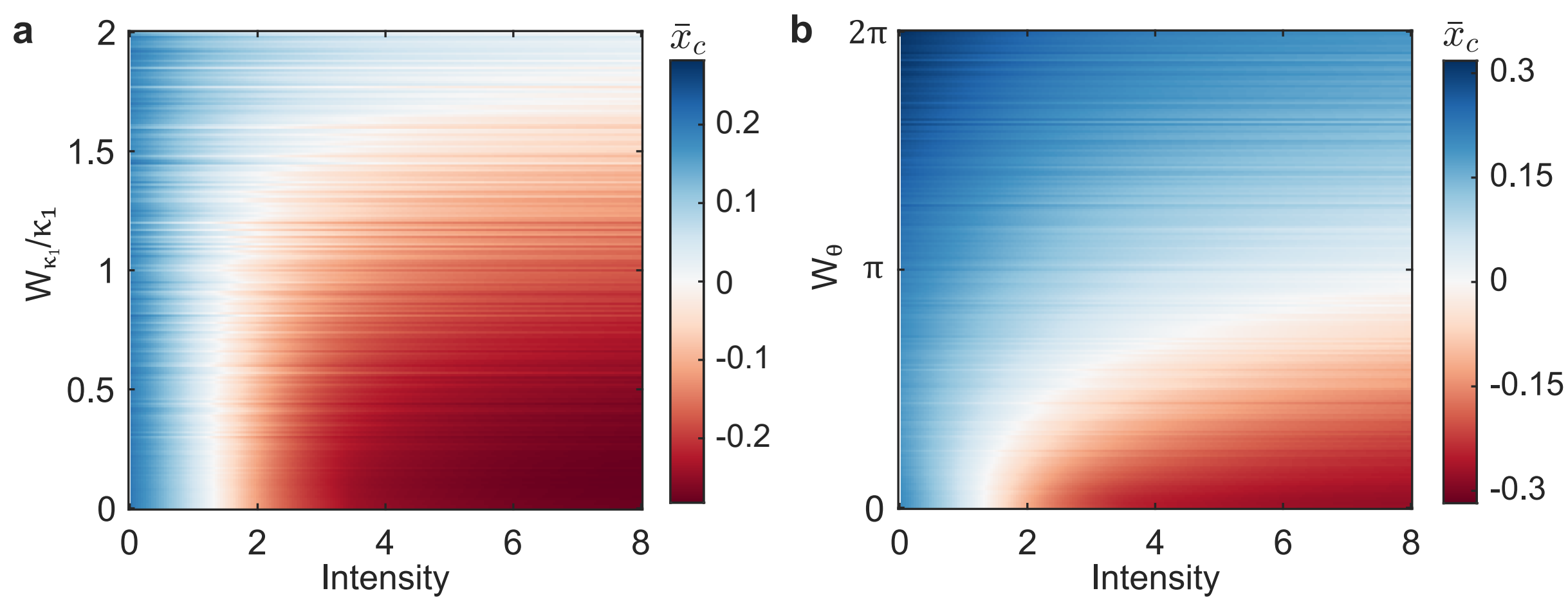

Fig. R6 The average position of the OBC eigenmodes as a function of total intensity and disorder strength. a Disorder in reciprocal coupling κ_1. **b** Disorder in coupling phase θ. Parameters are consistent with those in Fig. 1 of the main text. Results are averaged over 100 disorder realizations.

In the Supplementary Note 6, we have added a discussion on the robustness of the localization reversal, which reads: "In this section, we show that the reversal of the NHSE is a robust phenomenon. For this purpose, we calculate the average position $\bar{x}_c$ (defined in Eq. (3) in the main text) of the OBC eigenmodes as a function of the total intensity and the disorder strength. We consider two types of Anderson-type disorder: (1) disorder

in the reciprocal coupling κ_1, introduced as $\kappa_1' = \kappa_1 + \alpha$ with α a random number drawn from $[-W_{\kappa_1}/2, W_{\kappa_1}/2]$; (2) disorder in the coupling phase θ, introduced as $\theta' = \theta + \beta$ with β a random number drawn from $[-W_\theta/2, W_\theta/2]$. Here, W_{κ_1} and W_θ serve as the corresponding disorder strength. As shown in Fig. R6, the reversal of the NHSE persists when the disorder strength is below approximately $1.74\kappa_1$ (Fig. R6a) and 0.91π (Fig. R6b), respectively, demonstrating strong robustness of this phenomenon."

Reviewer comments:

c) Please quantify the localization length/skin depth (experiment vs theory) as a function of pump power.

Response:

We thank the reviewer for the suggestion. We have calculated the skin depth as a function of pump power for both the experimental setup and the theoretical model. Both theoretical and experimental results show consistent trends.

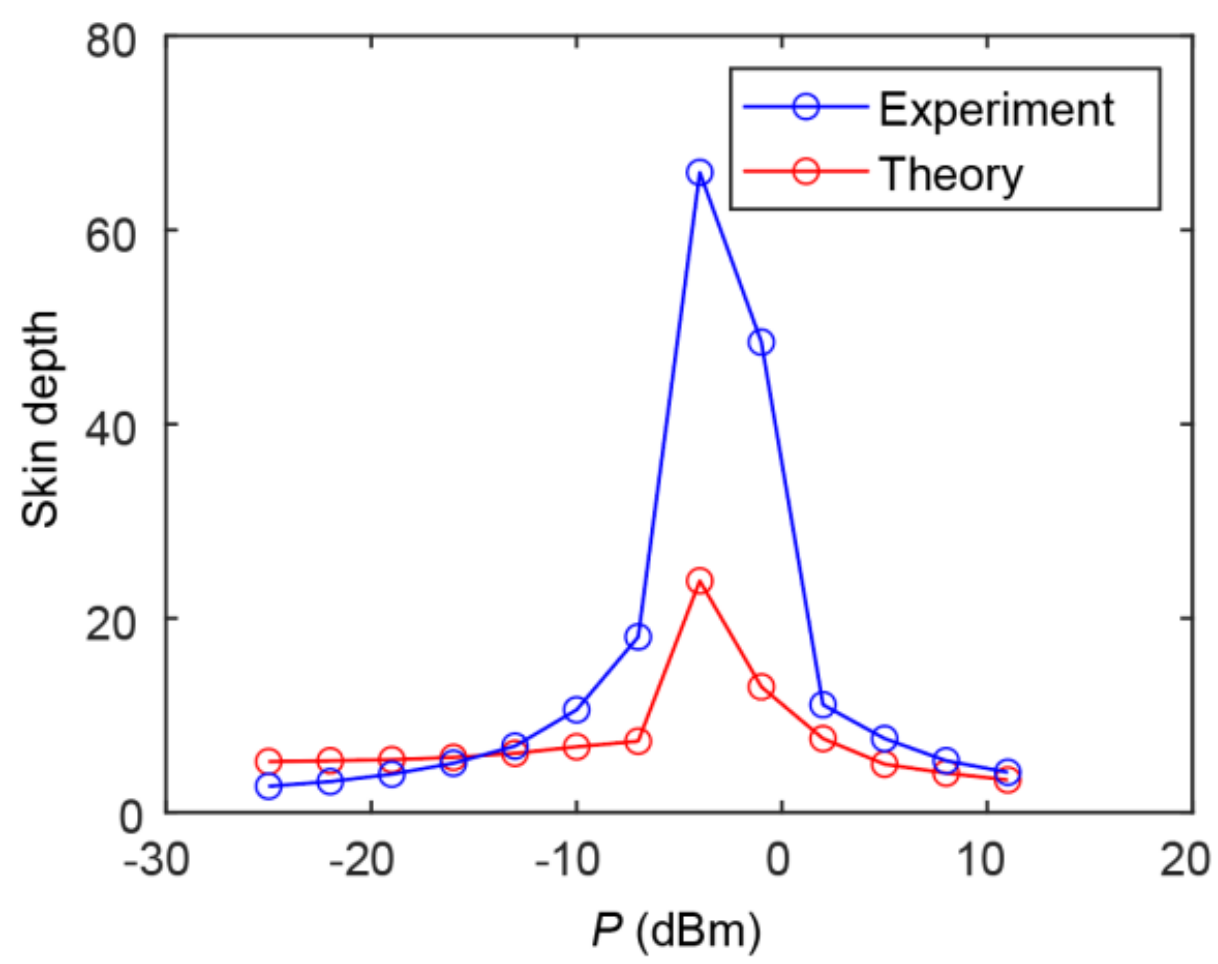

Fig. R7 Skin depth. The skin depth extracted from theory and experiment as a function of input power.

In the Supplementary Note 7, we have added a discussion on this point, which reads: "While the main text characterizes the localization direction of the NHSE by calculating the mean position, here we further compute the skin depth to quantify the degree of localization. The skin depth is extracted by fitting the wave-function profile near the localization peak with the form $\psi(x) \sim e^{-x/\xi}$, where ξ denotes the skin depth. For the left- and right-localized skin modes, we choose the corresponding boundary site as the fitting origin to ensure all ξ values are positive. The results in Fig. R7 demonstrate a consistent trend

between theoretical calculations and experimental measurements. The peaks in the curves, corresponding to large skin depths, serve as hallmarks of the localization reversal."

Reviewer comments:

3) It is unclear whether the system reaches a steady state before measurements. How long after adjusting the pump do the authors wait to measure? Are there transient or hysteretic effects that could complicate interpretation? Are there threshold intensities or hysteresis effects? For example, once the localisation has switched, does reducing the pump cause reversal back to the original end, or is there memory/hysteresis?

Response:

In the experiment, we wait for 100 ms after adjusting the pump before taking measurements, and the system reaches a steady state before our measurements. The corresponding time-domain waveform and clarifications on this issue have been added to the Supplementary Note 5, which reads:

"To visually verify the aforementioned nonlinear saturation effects and ensure the reliability of our spectrum measurements, we record the time-domain transient response of the system under discrete power steps at port 11 (Fig. R8). The 1 GHz excitation is generated by a Siglent SSG5060X-V signal generator, and the time-domain response is recorded using a Tektronix DPO7254 series digital phosphor oscilloscope (4 GHz bandwidth and 20 GS/s sampling capability).

For the measurements shown in Fig. R8, the oscilloscope is operated in a long time-window mode (40 ms/div, record length 20000 points) to capture the slow response dynamics under discrete power steps. Under these settings the carrier at 1 GHz is not cycle-resolved. Instead, the recorded trace reflects the slow-time evolution of the signal amplitude.

For a narrowband RF signal,

$$v(t) = A(t)\cos(2\pi f_0 t + \phi(t)), \tag{R14}$$

where $f_0 \approx 1$ GHz and $A(t)$ varies on the millisecond timescale, a long time-base measurement effectively tracks the envelope amplitude. Therefore, the plotted waveform should be interpreted as the slow-time intensity evolution of the 1 GHz signal. Importantly, the present configuration is not intended to reconstruct the carrier waveform within each RF cycle. The analysis focuses exclusively on steady-state amplitude levels and their dependence on the input power, which are fully encoded in the slow-time envelope. The magnitude of the en-

Input Power (dBm)	Normalized Amplitude (dB)
-30	0.000
-20	1.393
-10	7.729
0	15.933
10	20.862

Table R1 Normalized steady-state output amplitude referenced to the value at −30 dBm input.

velope is recorded for input powers ranging from −30 dBm to 20 dBm. As summarized in Table R1, the port voltage exhibits a nonlinear response to the input excitation. At 20 dBm input power, noticeable waveform distortion appears at both the peaks and troughs, indicating clear nonlinear compression. Additionally, the signal settles within approximately 1 ms after the intensity is switched, ruling out a transient origin of the observed effect."

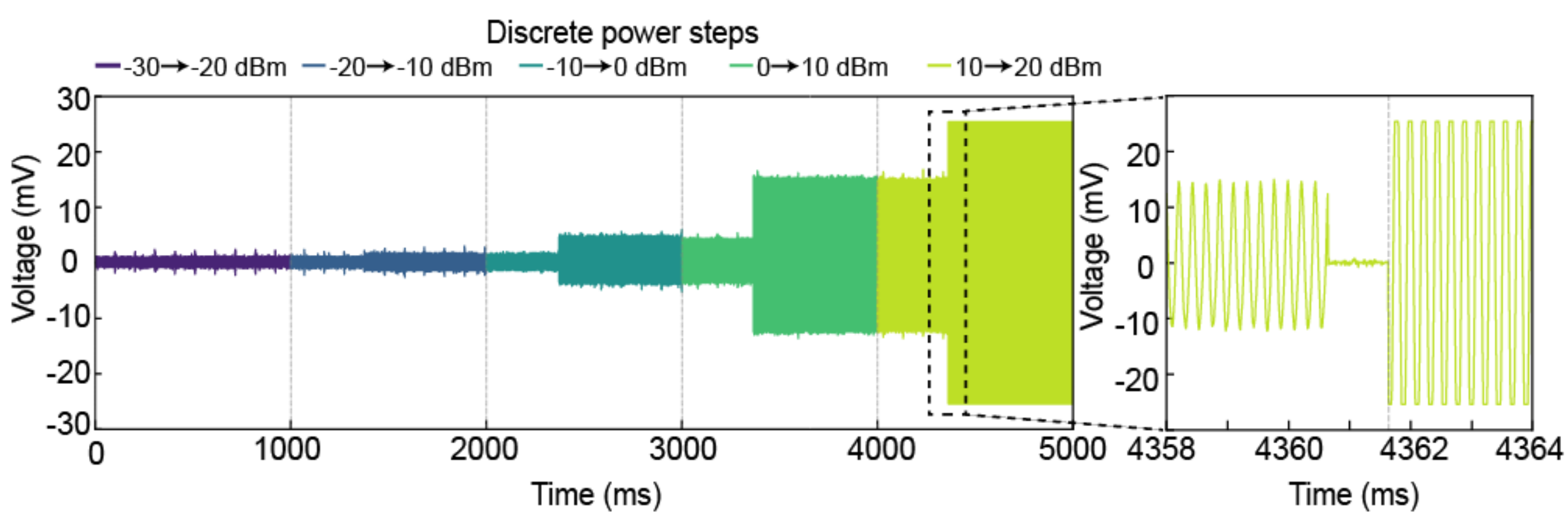

Fig. R8 Long time-base transient response of port 11 under discrete power steps. The trace represents the slow-time evolution of the signal amplitude.

Beyond confirming steady-state operation, we further examined the localization behavior upon increasing and decreasing pump power, observing no memory or hysteresis effects, as shown in Fig. R9.

Reviewer comments:

4) The authors claim this opens "a new route towards nonlinear topological physics in non-Hermitian systems". It may help to clarify the boundaries of that claim: is the effect specific to microwave metamaterials, or could it also apply in, e.g., photonic circuits, acoustic

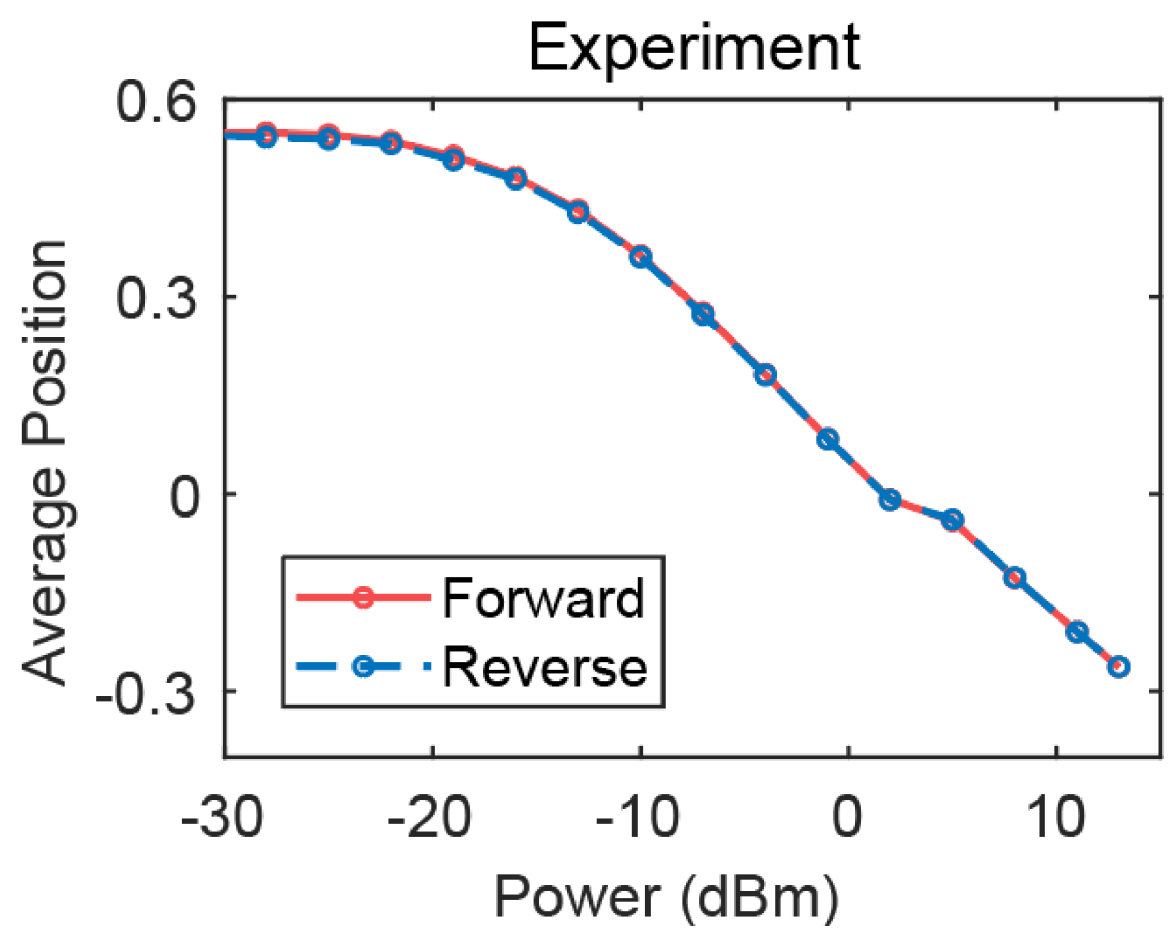

Fig. R9 Experimental average localization position of the energy distribution measured during increasing and decreasing pump-power sweeps, showing no observable hysteresis

systems, exciton–polariton systems? A short paragraph discussing generality and limitations would help.

Response:

We thank the reviewer for this helpful suggestion. We agree that clarifying the scope of our claim is important. The underlying mechanism—engineered nonreciprocal coupling combined with saturable nonlinearity is not restricted to microwave metamaterials in principle, although the practical implementation and accessible parameter window depend on platform-specific constraints. Regarding generality, we have added the following discussion to the conclusion part: "The key element in our system, i.e., amplifier-based nonreciprocal coupling combined with saturable nonlinearity, is not restricted to this specific implementation and is in principle applicable to other active wave platforms. Moreover, the current four-arm H-shaped resonator design naturally extends to two-dimensional networks, as each arm offers an independent coupling channel, facilitating planar connectivity [10, 11]. Hence, it would be highly desirable to extend our system to two dimensions, where novel physics emerging from the interplay among nonlinearity, non-Hermiticity, and band topology can be further explored." Regarding limitations, we have appended the following text to the section "Implementation of nonlinear nonreciprocal coupling at microwave frequencies" in the main text: "In practice, the experimentally accessible parameter window of the coupling is limited by component tolerances, amplifier nonlinear operating range, and measurement

noise, which may induce resonator detuning and introduce additional effective gain or loss. However, by choosing high-quality LNA and conducting careful calibration, the desired parameter values can be reached."

Reviewer comments:

5) It might be useful to comment on how the effect scales: for larger system sizes, different strengths of nonlinearity, or in higher dimensions, would one expect similar reversal behaviour?

Response:

Firstly, the localization reversal phenomenon is not affected by system size. As explained in the response to Reviewer #2's comment 6, we have the numerical results for $N = 21$ (Fig. 1) and $N = 11$ (Fig. 3e-h), both of which show the reversal behavior. To emphasize this point, we have added the following remark in the main text on page 5, left column: "Note that we use fewer resonators ($N = 11$) to facilitate the experimental implementation, and the localization reversal phenomenon persists under the size change of the system."

Secondly, the strength of nonlinearity does have a noticeable impact. Figure R10 shows the phase diagrams calculated under different nonlinearity strengths (controlled by t_c in our model). As can be seen, the localization reversal occurs in all cases. However, with stronger nonlinearity (i.e., smaller t_c), the reversal happens more easily (i.e., at lower intensity).

Lastly, we think a similar phenomenon can also occur in higher dimensions, as the basic mechanism, i.e., the intensity-dependent non-Hermitian coupling, is not restricted by dimensionality. However, since the NHSE in higher-dimensional systems is much more complicated

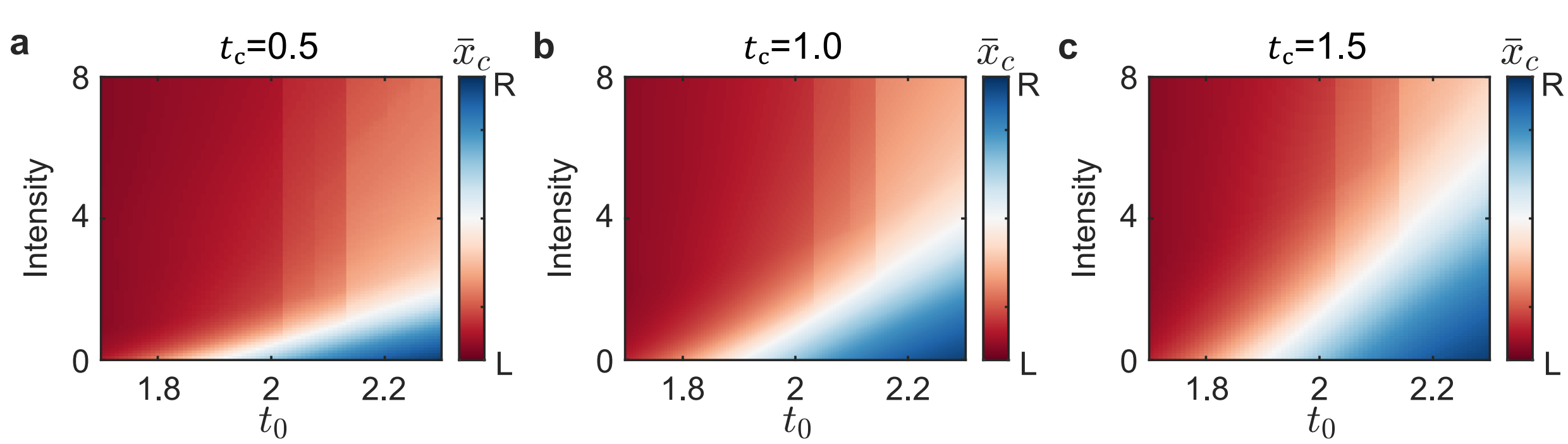

Fig. R10 The average position of all OBC eigenvectors as functions of the intensity I and t_0, for different nonlinear strengths $t_c = 0.5$ **a**, $t_c = 1.0$ **b**, and $t_c = 1.5$ **c**. The remaining parameters are consistent with those in Fig. 1 of the main text.

than the 1D case, we leave this possibility for future studies. In the revised manuscript, we have added two sentences to mention the higher-dimensional possibility in the conclusion part, which reads: "Moreover, the current four-arm H-shaped resonator design naturally extends to two-dimensional networks, as each arm offers an independent coupling channel, facilitating planar connectivity [10, 11]. Hence, it would be highly desirable to extend our system to two dimensions, where novel physics emerging from the interplay among nonlinearity, non-Hermiticity, and band topology can be further explored."

Reviewer comments:

6) Are there trade-offs or regimes where the nonlinearity could destroy the skin effect (rather than reverse it)?

Response:

We thank the reviewer for the insightful question. Indeed, such regimes do exist in our system. The reversal of the NHSE is achieved under carefully chosen parameters, as stated in page 3 of the main text: "A threshold, given by $|\kappa_1 + \tilde{\kappa}_{2,n}(I_n \to 0)| > \kappa_1$, is required, such that rightward couplings are dominant at zero intensity. In addition, the high-intensity coupling needs to satisfy $|\kappa_1 + \tilde{\kappa}_{2,n}(I_n \to \infty)| < \kappa_1$ to make the reversal happen." When the parameters do not satisfy this condition, as shown in Fig. R11, the eigenvectors transition from right localization to delocalization as the intensity increases, thereby leading to the destruction of the skin effect.

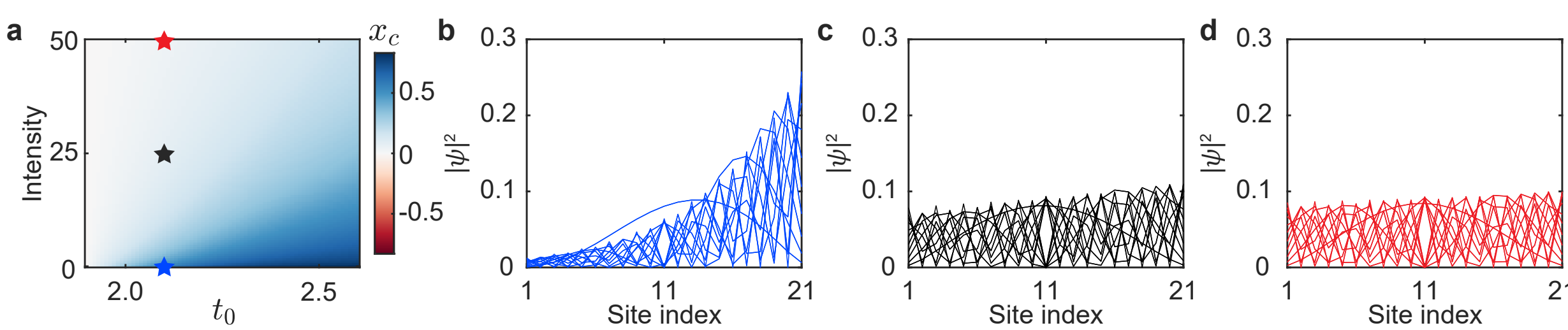

Fig. R11 a Average position of all OBC eigenvectors as functions of the intensity I and t_0. **b-d** Plots of OBC eigenvectors corresponding to the three intensity values marked by the blue **b**, black **c**, and red **d** stars in **a**, respectively. The parameters are $f_0 = -2.5i$, $\kappa_1 = 1$, $t_\infty = 1.9$, $t_c = 0.2$ and $\theta = -0.9\pi$.

Reviewer comments:

7) While harmonic generation is highlighted in the title, its connection to NHSE reversal is not clearly established.

Response:

We thank the reviewer for raising this important point. In our system, harmonic generation originates from the nonlinear response of the same resonator network that hosts the NHSE. As a result, the spatial distribution of the generated harmonics inherits the localization characteristics of the fundamental mode, including its reversal. More detailed clarifications are provided below.

Reviewer comments:

a) Are higher harmonics also subject to localization reversal?

Response:

The 3rd harmonic signal is also subject to localization reversal (there are no bands at the 3rd harmonic frequency), as shown in Fig. 4c,d in the main text.

For higher-order ones (beyond the 3rd order), theoretically, they can also show the reversal when there are no bands at the corresponding frequencies. Otherwise, the field distribution will be reshaped according to the dispersion. Experimentally, the observation of higher-order harmonics (beyond the 3rd) is limited by the specific characteristics of the low-noise amplifiers (LNAs) used in our setup. However, the mechanism of localization reversal is generic to all harmonics generated by this nonlinearity. As discussed in Supplementary Note 5, the generation of harmonics is a local process driven by the energy distribution of the fundamental mode.

Reviewer comments:

b) Do harmonics carry their own topological signatures, or are they by-products of nonlinearity?

Response:

Thank you for your insightful question. We note that harmonics in our experiment do not exhibit their own topological signatures. As mentioned above, there are no bands at the 3rd harmonic frequency. In other words, the harmonic signals are detuned from resonator eigenmodes. As a result, the harmonics behave as by-products of the nonlinear response, rather than showing independent topological behavior. But the scenario raised by the reviewer is insightful and will be helpful for guiding future investigations into harmonic-related topological effects.

Reviewer comments:

c) Please include comparative spectra of fundamental and harmonic intensities across the pump-induced transition.

Response:

We have added comparative measurements of the fundamental and third-harmonic responses across the pump-induced transition in Supplementary Note 5. In particular, Fig. R12 presents the corresponding spatial field distributions at representative input powers, which directly visualize how the third-harmonic response follows the reversal of the fundamental mode. "The electrical field distributions of the fundamental and third harmonics are depicted in Fig. R12. Here, the third-harmonic distribution directly inherits the reversal of the fundamental harmonic. Since there are no eigenmodes at the third harmonic frequency, the higher-order harmonic distribution is governed by the fundamental mode, enabling nonlinear control of its spatial localization."

Reviewer comments:

Additional Minor but Helpful Suggestions

a) Define "localization reversal" more precisely early in the paper–does it refer to mode center shifting, change in decay direction, or a sign change in the skin depth?

Response:

Following the reviewer's suggestion, we have added the following sentence in the revised introduction: "This phase transition leads to a "localization reversal" phenomenon: the modes originally localize at the right boundary are relocated to the left boundary."

Reviewer comments:

b) Improve figure captions by including pump intensity values, normalization conventions, and experimental condition. Ensure consistency in notation between theory and experiment sections (e.g., use same symbols for nonlinear strength)

Response:

We thank the reviewer for this suggestion. We have revised the figure captions to explicitly clarify the relevant theoretical and experimental details, and ensured consistency between the theory and experiment sections.

Reviewer comments:

c) The authors may Include a schematic of how the nonlinearity modifies the spectrum in the complex plane with proper winding–this would make the topological picture much clearer.

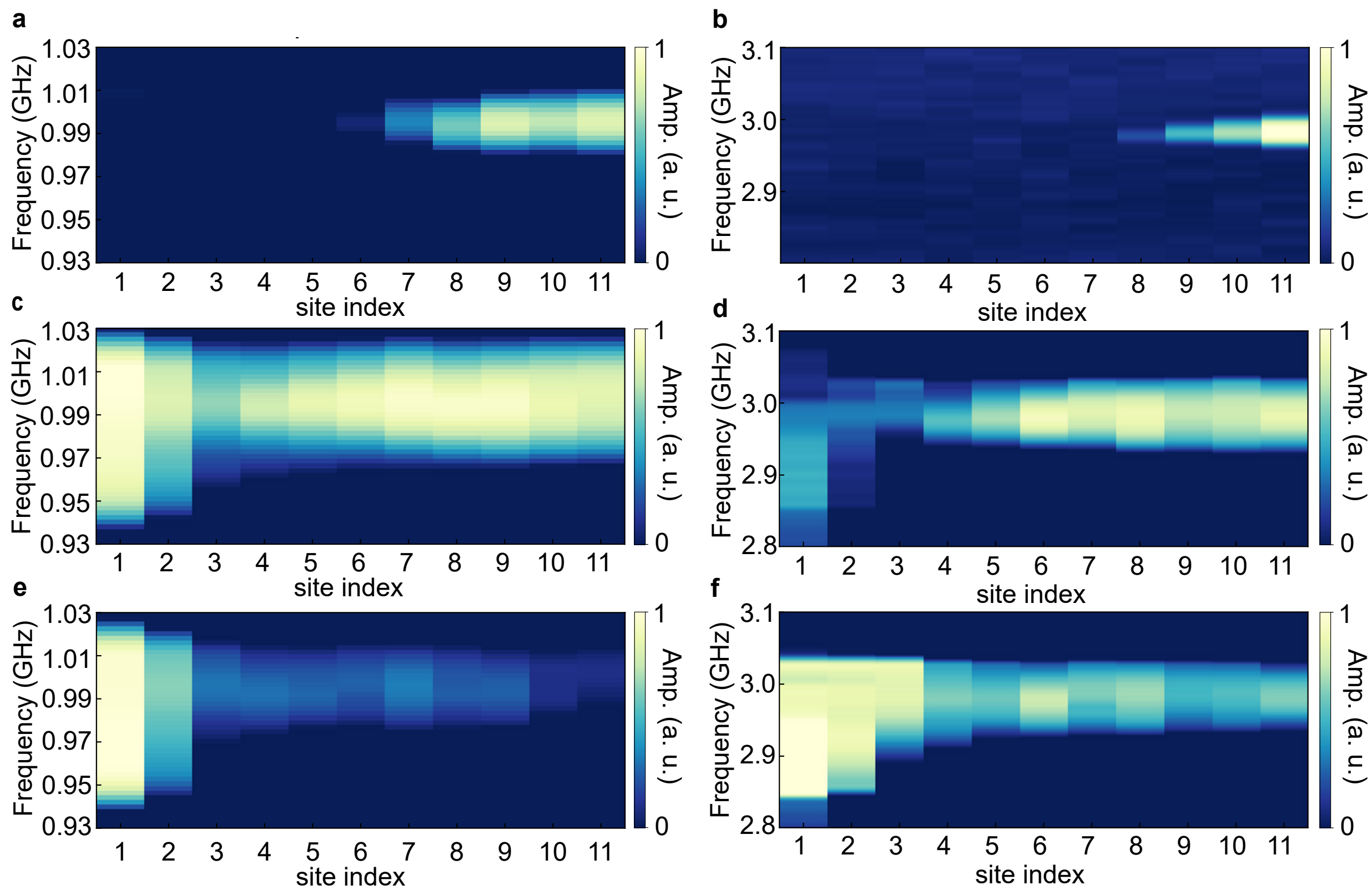

Fig. R12 Observation of harmonics reversal of the NHSE via port measurements. Measured spatial field distributions of the fundamental harmonic at input powers of -25 dBm **a**, 0 dBm **c**, and 25 dBm **e**. Corresponding spatial field distributions of the third harmonic at -25 dBm **b**, 0 dBm **d**, and 25 dBm **f**. Each panel is independently normalized to its maximum response at the corresponding input power.

Response:

As discussed in our response to Comment 1(c), we have clarified the topological picture in two complementary ways. In the main text (Fig. 1d,f), we now explicitly indicate the winding direction of the complex spectrum in the complex plane, which directly visualizes how the spectral winding reverses across the transition. In addition, in Supplementary Note 1 (Fig. S1), we present the winding number as a function of intensity, characterizing the topological origin of the localization reversal more clearly.

[1] E. N. Economou. *Green's functions in quantum physics*. Springer Series in Solid-State Sciences. Springer Berlin Heidelberg, 2013.

[2] Yuto Ashida, Zongping Gong, and Masahito Ueda. Non-Hermitian physics. *Adv. Phys.*, 69(3):249–435, 2020.

[3] Claude Mahaux and Hans A. Weidenmüller. *Shell-model approach to nuclear reactions*. North-Holland Publishing Company Amsterdam, 1969.

[4] Zongping Gong, Yuto Ashida, Kohei Kawabata, Kazuaki Takasan, Sho Higashikawa, and Masahito Ueda. Topological phases of non-Hermitian systems. *Phys. Rev. X*, 8(3):031079, 2018.

[5] Shunyu Yao and Zhong Wang. Edge states and topological invariants of non-Hermitian systems. *Phys. Rev. Lett.*, 121(8):086803, 2018.

[6] Wei Wang, Xulong Wang, and Guancong Ma. Non-Hermitian morphing of topological modes. *Nature*, 608(7921):50–55, 2022.

[7] Yakir Hadad, Alexander B Khanikaev, and Andrea Alu. Self-induced topological transitions and edge states supported by nonlinear staggered potentials. *Phys. Rev. B*, 93(15):155112, 2016.

[8] Weiwei Zhu, Wei Xin Teo, Linhu Li, and Jiangbin Gong. Delocalization of topological edge states. *Phys. Rev. B*, 103(19):195414, 2021.

[9] Wu-Pei Su, John Robert Schrieffer, and Alan J Heeger. Solitons in polyacetylene. *Phys. Rev. Lett.*, 42(25):1698, 1979.

[10] Christopher W Peterson, Wladimir A Benalcazar, Taylor L Hughes, and Gaurav Bahl. A quantized microwave quadrupole insulator with topologically protected corner states. *Nature*, 555(7696):346–350, 2018.

[11] Wenwen Liu, Junyao Wu, Li Zhang, Oubo You, Ye Tian, Hongsheng Chen, Bumki Min, Yihao Yang, and Shuang Zhang. Quantized decay charges in non-Hermitian networks characterized by directed graphs. *Phys. Rev. Lett.*, 135(20):206602, 2025.

Response Letter to Reviewers

We sincerely thank the editor and the reviewers for their continued effort and constructive feedback on this manuscript (NCOMMS-25-80794A). We have carefully addressed all remaining concerns raised by Reviewers #2 and #3, with particular attention to the issues of reproducibility, clarity of definitions, and the physical novelty of our findings. In what follows, the comments from each reviewer are quoted in *blue italics*, and followed by our point-by-point response. The manuscript and Supplemental Information have been further revised accordingly, with all updates highlighted in red. For the reviewers' convenience, key figures added to or discussed in the revised manuscript and Supplementary Information are reproduced in this response letter and labeled as Fig. R1, Fig. R2, etc. Throughout this response letter, we refer to these reproduced figures using the R-numbering system.

Reviewer #2

Reviewer comments:

In the revised version of the manuscript, the authors address some of my concerns. However, they did not attempt to address my main concern—namely that I could not find any discussion of what new physics this work might reveal. Without this clarification, the work appears primarily technical.

Response:

We respectfully disagree with the reviewer's assessment. The new physics of this work is the discovery that nonlinearity can drive a point-gap topological phase transition, a type unique to non-Hermitian systems with no Hermitian counterpart. All previous nonlinearity-induced topological transitions (Refs. [50–54] in the main text) are restricted to the line-gap regime. Our work extends nonlinear topological physics into the point-gap regime for the first time, both theoretically and experimentally.

A direct physical consequence is that the direction of the NHSE can be reversed by tuning input power alone, without any structural modification, establishing energy, rather than geometry, as a new control knob for non-Hermitian skin localization. Furthermore, the harmonic signals generated by the skin modes inherit this reversal, connecting nonlinear frequency conversion with non-Hermitian topology for the first time.

To make these points more prominent, we have revised the introduction and conclusion of the manuscript accordingly. Specifically, in the introduction, we have added the following

statement to clarify the open question addressed by this work: "However, whether nonlinearity can induce a topological phase transition between different nontrivial point-gap phases remains unexplored." In the conclusion, we have expanded the discussion to read: "On the fundamental level, our results demonstrate that nonlinearity can drive point-gap topological phase transitions in non-Hermitian systems, enabling the reversal of the NHSE through input power rather than structural modification. The harmonic signals generated by the skin modes further inherit this reversal, revealing an intrinsic connection between nonlinear frequency conversion and non-Hermitian topology."

Reviewer comments:

Moreover, the authors have introduced new material that, in my view, deviates significantly from the original content and makes the work even less reproducible than it was before the revision.

Response:

The nonlinear SSH model was added in the previous revision following the explicit suggestion of Reviewer #3, who asked "It would be interesting to know, when there are both topological modes and skin modes (like in non-reciprocal SSH model), how the localization reversal phenomena would work?" This extension is not a deviation from the original content, but a natural generalization that demonstrates the broader applicability of our findings beyond the Hatano-Nelson model. The SSH model supports both point-gap and line-gap topological phases, and our results show that nonlinearity can simultaneously drive both types of transitions within the same system, which further strengthens the physical significance of this work.

Reviewer comments:

Even more surprising is the removal of formulas that seem important, such as Y_{ii} (formerly in Eq. (S7)). This gives the impression that at least some of the equations serve a decorative rather than a substantive role in the manuscript; otherwise, it is difficult to understand why they would be omitted or left undefined (see below).

Response:

The Y_{ii} terms were not removed in the revision. They appear in the same location in both versions: in the text following the Kirchhoff equations, where we state that Y_{11}, Y_{22}, and Y_{12} are sufficiently small to be neglected. The Kirchhoff equations are derived from

first principles and do not cause ambiguity. The omission of these terms reflects a well-established property of the unidirectional amplifier, and retaining them in the formulas would be decorative rather than substantive. We address the remaining specific concerns individually below.

Reviewer comments:

Starting with the last point: in Note 1 the authors define the winding number in Eq. (S1) using a "reference point" E_B. This reference point is not defined anywhere in the main text or in the Supplementary Note, and the specific value of E_B used to produce Fig. S1 is not stated. More importantly, $H(\Phi)$ appearing in Eq. (S1) is not defined. What is it supposed to represent and how computed? In the main text (Methods) one finds $H(\psi)$ which is clearly a different object, because Φ and ψ denote entirely different quantities (an argument and a field, respectively). As written, a reader has no way to reconstruct how Eq. (S1) is obtained or how Fig. S1 was computed.

Response:

$H(\psi)$ in the Methods describes the nonlinear dependence of the Hamiltonian on the field amplitude. $H(\Phi)$ in Eq. (S1) is the PBC Hamiltonian with magnetic flux Φ threaded, where the coupling strengths are already fixed by a given eigenmode. They are different quantities, and hence there is no conflict. In the revised Supplementary Note 1, we have added the explicit definition of $H(\Phi)$ and the value of E_B after Eq. (S1).

"Here, $H(\Phi)$ is the Hamiltonian obtained by threading the magnetic flux Φ into the PBC Hamiltonian whose coupling strengths are determined by the intensity distribution of a given PBC eigenmode via Eq. (2) in the main text. E_B is chosen inside the point gap, and the winding number is invariant to its specific value. In our calculations, we take $E_B = 4-2.5\mathrm{i}$."

Reviewer comments:

The new paragraph entitled "Nonlinear nonreciprocal SSH model" is even more obscure. It appears to describe a different setting (schematically shown in Fig. 5a), yet it provides only general statements and a few plots in Fig. 5, without the supporting formulas, definitions, or theoretical framework needed to connect the model to the displayed results. As a consequence, a reader cannot follow the authors' reasoning or reproduce the results presented in this paragraph.

Response:

We would like to point out that the nonlinear SSH model shares the same coupled-mode equation (Eq. (1)) and the same nonlinear coupling model (Eq. (2)) as the Hatano-Nelson model discussed in the main text. The only difference lies in the lattice geometry, where alternating intracell and intercell couplings are adopted. All parameters used in the calculations have been listed in the caption of Fig. 5. Following the reviewer's comment, we have added the explicit theoretical framework in the revised Supplementary Note 8, including the coupled-mode equations and the Hamiltonian matrix for the SSH model.

Reviewer comments:

Further, in Note 7 the authors claim that they fitted "the wave-function profile near the localization peak" with an exponential form. However, they do not show the fit itself. Instead, Fig. S8 shows a rather poor agreement between theory and experiment: the predicted peak depth is more than three times smaller than the experimental value. Moreover, even this limited comparison cannot be reproduced, because the authors provide no fitting procedure, parameters, fitting window, or other essential details.

Response:

The discrepancy between the theoretical and experimental peak values in Fig. S8 arises from the inherent sensitivity of the system near the localization reversal point, where the skin depth diverges. In this regime, even a slight deviation in parameters can lead to a substantial change in the peak value, making precise quantitative agreement inherently difficult. The physically meaningful comparison is the overall trend, which shows consistent agreement between theory and experiment across the full power range. We have added the detailed fitting procedure in the revised Supplementary Note 7, as described below.

"The skin depth ξ characterizes how far a skin mode extends from the localization boundary, defined through the exponential decay

$$\psi(x) \sim e^{-x/\xi}, \tag{R1}$$

where x is the distance from the localization boundary. A smaller ξ means the mode is strongly confined near the boundary, while a larger ξ indicates a more spatially extended mode. At the topological phase transition point, ξ diverges, meaning the mode becomes completely delocalized. Beyond this point, the mode relocalizes at the opposite boundary.

To extract ξ, we take the natural logarithm of both sides:

$$\ln \psi(x) = -\frac{1}{\xi} x + C, \tag{R2}$$

where C is a constant. This transforms the exponential decay into a linear relation. A least-squares linear fit to $\{(x_j, \ln \psi_j)\}$ across all lattice sites yields the slope k, from which the skin depth is obtained as $\xi = -1/k$. For each input power, the localization boundary is identified as the site with the maximum field amplitude."

Reviewer comments:

2. I am afraid the authors did not get the point. "Without loss of generality" means that a parameter can be scaled out completely (or eliminated by a redefinition of variables), so that it does not affect the generality of the results. This is not the same as choosing a value that is merely "typical" or representative.

What Fig. R1 illustrates is that the chosen parameters may be typical within some range, but it does not demonstrate that the conclusions hold in full generality. To justify "without loss of generality," the authors would need to show an explicit scaling argument (or prove invariance of the relevant dimensionless quantities), rather than only providing a numerical example or a parameter sweep over a limited interval. Furthermore, later in the text the parameter κ_1 appears again. What is it, considering that it was set "without loss of generality" $\kappa_{1,n} = 1$?

Response:

In our theoretical model, the reciprocal coupling $\kappa_{1,n}$ is uniform across all sites. We denote this constant by $\kappa_{1,n} \equiv \alpha$. The eigenvalue equation derived from Eq. (1) is $H\psi = E\psi$, where the Hamiltonian has matrix elements

$$H_{n,n} = f_0, \quad H_{n,n+1} = \alpha, \quad H_{n,n-1} = \alpha + \tilde{\kappa}_{2,n}(I_n). \tag{R3}$$

Dividing both sides by α, we obtain

$$\tilde{H}\psi = \tilde{E}\psi, \tag{R4}$$

where $\tilde{H} = H/\alpha$, $\tilde{E} = E/\alpha$, and

$$\tilde{H}_{n,n} = \frac{f_0}{\alpha}, \quad \tilde{H}_{n,n+1} = 1, \quad \tilde{H}_{n,n-1} = 1 + \frac{\tilde{\kappa}_{2,n}(I_n)}{\alpha}, \tag{R5}$$

with the nonlinear coupling in Eq. (2) rescaling as

$$\frac{\tilde{\kappa}_{2,n}(I_n)}{\alpha} = \left(\frac{t_0/\alpha - t_\infty/\alpha}{1 + I_n/t_c} + \frac{t_\infty}{\alpha}\right) e^{i\theta}. \tag{R6}$$

Crucially, H and $\tilde{H}$ share exactly the same eigenvectors ψ, with eigenvalues related by $E = \alpha\,\tilde{E}$. Setting $\alpha = 1$ is therefore equivalent to expressing all energy-scale parameters (f_0, t_0, t_∞, E) in units of α. All physical conclusions drawn from Fig. 1 are preserved:

(i) The average position $\bar{x}_c$ in Eq. (3) depends solely on $|\psi_j|^2$, which is invariant under the rescaling.

(ii) The eigenvalues in Fig. 1d–f are uniformly rescaled by $1/\alpha$, which preserves the winding topology of the PBC spectrum.

(iii) The localization direction and spatial profile of the eigenvectors in Fig. 1g–i are determined by ψ, which remains identical.

Therefore, α can be rigorously factored out, and setting $\alpha = 1$ constitutes a normalization where all parameters are expressed in units of α. The $\kappa_1 = 66.2$ MHz in the experimental sections is the physical value of α retrieved from measurements (Supplementary Note 2).

Reviewer comments:

5. Eq. (3) does not look meaningful as written. It is unclear what is meant by "the average over all modes" $\langle\cdot\rangle$? How is it computed exactly? This is particularly problematic in the nonlinear case: the number of stationary solutions can exceed that of the linear limit (bistability being the simplest example). In that situation, "average over all modes" is ambiguous unless the authors explain which branch(es), if there are more than one, are included.

If, instead, the authors actually mean an average over the linear eigenmodes only, then $\bar{x}_c$ is just a fixed number determined by the linear spectrum and should be explicitly provided, along with the precise procedure used to compute it. I emphasize that I am talking about averaging $\langle\cdot\rangle$, rather than about trivial formula (R3).

Response:

The average $\langle\cdot\rangle$ in Eq. (3) is taken over all eigenmodes obtained at a given total intensity. Equation (3) is not fundamentally different from its original form; the updated expression simply condenses the sum over all eigenmodes into the compact average notation $\langle\cdot\rangle$. All eigenmodes here are obtained using the nonlinear self-consistent method (see Methods for details). For a lattice chain with N sites, this self-consistent method yields the evolution of the N eigenvalues and eigenvectors as functions of the input intensity. Averaging over all

eigenmodes, denoted by $\langle\cdot\rangle$, yields the average position $\bar{x}_c$ at each intensity, which serves as the criterion to identify the reversal of the NHSE.

To avoid ambiguous wording, we have revised the description in the manuscript from "$\langle\cdot\rangle$ denotes the average over all modes" to "$\langle\cdot\rangle$ denotes the average over all eigenmodes".

Notably, in our previous response to Reviewer #3, we experimentally verified that no multistable branches appear in the present system under either forward or backward power scanning. This ensures that the average over all eigenmodes is well-defined and unambiguous in our nonlinear analysis.

Reviewer comments:

7. The parameter κ_1 is not defined in the text.

Response:

κ_1 is the uniform value of $\kappa_{1,n}$ with the site index dropped for brevity. It appears in both the theoretical and experimental sections, representing the same physical quantity in different units. To make this explicit, we have revised the main text (page 3, left column) to read: "Since $\kappa_{1,n}$ is uniform across all sites, we write it as κ_1 hereafter. In the theoretical model, we set $\kappa_1 = 1$ as the normalization unit, while in the experimental sections κ_1 takes its physical value retrieved from measurements."

In addition, we have supplemented the description of Eq. (4) to explicitly define all parameters in the experimental Hamiltonian."where f_0 is the resonance frequency of the individual resonators, κ_1 and $\tilde{\kappa}_2$ are the reciprocal and nonreciprocal couplings."

Reviewer comments:

8. The Mahaux–Weidenmüller formula is typically derived for linear systems and is not applicable to nonlinear ones. Therefore, the meaning of the Note 2 remains unclear, unless the authors can justify the use of the Mahaux–Weidenmüller formula for a nonlinear system, or provide a reference where such an extension is derived.

Response:

We would like to clarify that the nonlinearity in our system resides in the power dependence of the coupling coefficient $\tilde{\kappa}_2$, not in the Hamiltonian structure. For any given input power, the Hamiltonian in Eq. (4) is a well-defined matrix with fixed entries, to which the Mahaux-Weidenmüller formula directly applies. The nonlinear model is then constructed by tracking how the extracted $\tilde{\kappa}_2$ varies with power (Eqs. (S8) and (S9)). This approach

is well established in the literature. For instance, Assawaworrarit *et al.* (Nature **546**, 387 (2017)) employed coupled-mode theory to model a nonlinear parity-time-symmetric circuit with saturable gain, and Bai *et al.* (Phys. Rev. Lett. **132**, 073802 (2024)) adopted the same strategy for nonlinear exceptional points.

Reviewer comments:

10. The authors' justification has a problem. In general, Fourier analysis is not directly applicable to nonlinear systems such as the one considered.

Response:

In Eq. (R5) of our previous response, H_{eff} is a constant matrix for any given operating condition, since $\tilde{\kappa}_2$ depends on the steady-state field intensity $|\psi_n|^2$, not on time t. The equation $\frac{i}{2\pi}\frac{d}{dt}\psi(t) = H_{\text{eff}}\psi(t) + F(t)$ is therefore a linear constant-coefficient differential equation in $\psi(t)$, to which the Fourier transform applies rigorously. The nonlinearity manifests in the dependence of H_{eff} on the operating power, not in the time evolution governed by Eq. (R5).

Reviewer comments:

12. Remains the nonlinearity issue mentioned above.

Response:

The derivation of the scattering coefficients in Supplementary Note 2 follows the same logic as discussed in our response to Comment 8. The Green's function $G(f) = [fI - H_{\text{eff}}]^{-1}$ and the scattering relation $S(f) = I - W^{\dagger}G(f)W$ involve H_{eff} as a fixed matrix for any given operating condition. The nonlinearity enters only through the power dependence of $\tilde{\kappa}_2$ within H_{eff}, not through the formalism itself.

Reviewer comments:

14. Quantity Y_{ii} disappeared from the new SI without explanations why.

Response:

Y_{ii} did not disappear from the revised SI because it was never present in the equations in either version. In both the original and revised Supplementary Note 3, the Kirchhoff equations retain only Y_{21}, with the text immediately below explaining that Y_{11}, Y_{22}, and Y_{12} are negligible. No content was removed between the two versions. For the reviewer's reference, we show the four admittance parameters of the MGA-53543 chip (derived from its official datasheet) in Fig. R1. Across the operating frequency range, $|Y_{21}|$ is at least 22 dB larger than all other admittance parameters. Physically, Y_{11} and Y_{22} contribute only to

the diagonal loss terms of the Hamiltonian. Their absolute values are both below −34 dB, far too small to introduce any appreciable asymmetry between the two resonators, and their effects are absorbed into the intrinsic loss rate γ_0 retrieved from experimental fitting (Supplementary Note 2). Y_{12} is the smallest among all four parameters and can be safely neglected.

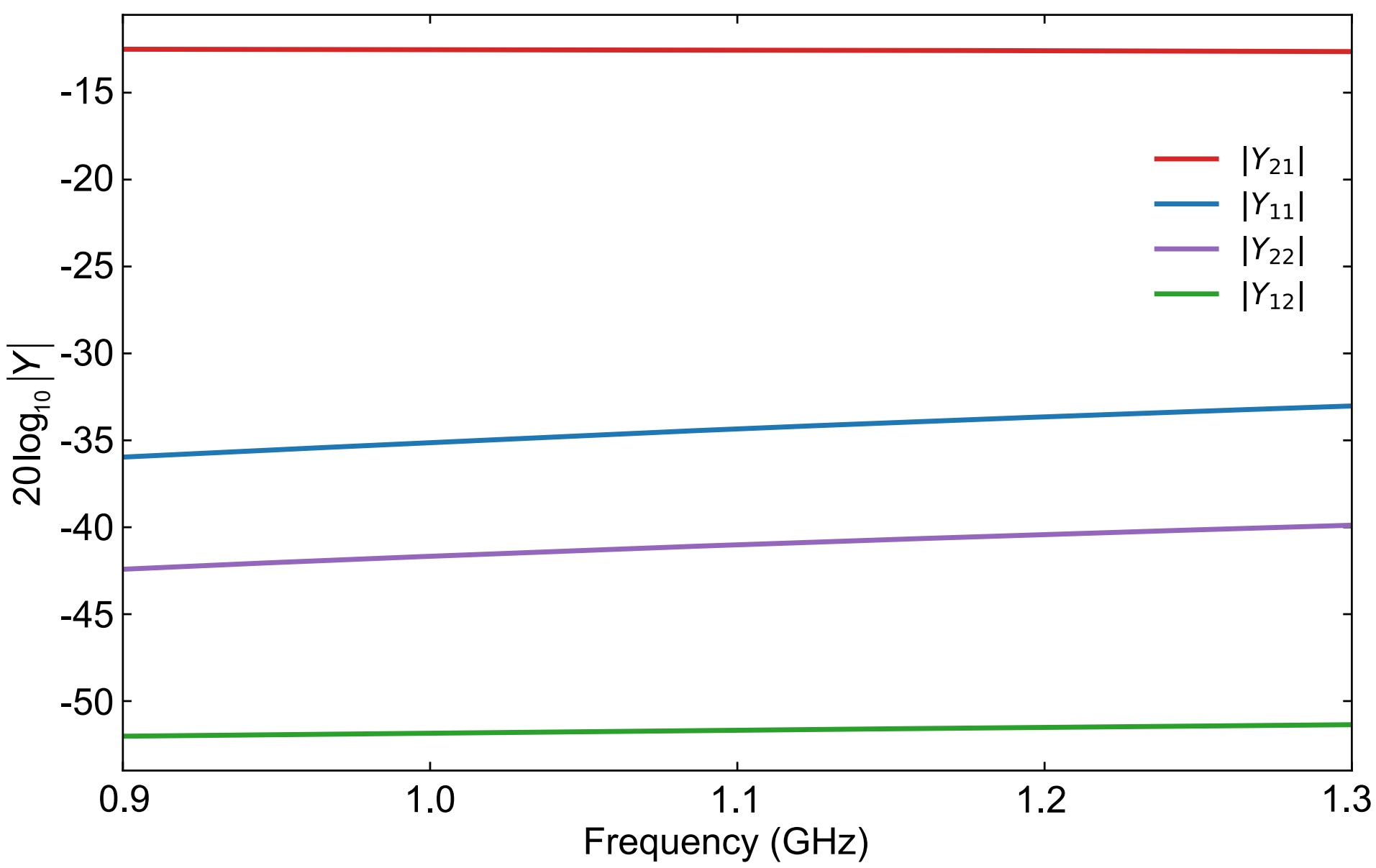

Fig. R1 Admittance parameters of the MGA-53543 amplifier derived from the official datasheet S-parameters. $|Y_{21}|$ is at least 22 dB larger than the other three parameters across the operating frequency range, justifying the approximation of retaining only Y_{21} in the Kirchhoff equations.

Reviewer comments:

15. v_o and v_i in the SI are not related to any of the quantities in the main text, thus their meaning in the context of the present work remains unexplained.

Response:

v_i and v_o are the time-domain input and output voltage signals of the amplifier, as defined in the revised Supplementary Note 5. Physically, v_i is the RF voltage at the amplifier input port, set by the field at that site. Supplementary Note 5 uses these standard RF notations to analyze the harmonic generation within a single amplifier stage. The connection to the main text is through the nonlinear coupling $\tilde{\kappa}_2$, which originates from Y_{21} of this amplifier as derived in Supplementary Note 3.

Reviewer comments:

In summary, I do not consider the present work suitable for publication.

Response:

All concerns raised in this round have been addressed in detail above, with corresponding revisions to the manuscript and Supplementary Information. We hope the reviewer will reconsider the assessment.

Reviewer #3

Reviewer comments:

I thank the authors for their careful revisions. I am generally satisfied with their responses, and I believe they have made a genuine effort to address the referee reports and improve the clarity and overall quality of the manuscript. That said, I feel that the draft would still benefit from additional clarification and justification. I outline a few remaining points below:

Response:

We sincerely thank Reviewer #3 for the positive assessment and the continued constructive feedback. Below we address each remaining point in detail.

Reviewer comments:

The authors state that "the peaks in the curves, corresponding to large skin depths, serve as hallmarks of the localization reversal." This statement would benefit from a more explicit explanation. As it stands, the connection is not entirely clear. My understanding is that a large skin depth corresponds to a stronger non-Hermitian skin effect, i.e., a higher degree of localization toward the boundary. It would therefore be helpful if the authors could clarify how an increase in skin depth signals a localization transition or reversal, and clearly distinguish this from conventional notions of localization strength.

Response:

We thank the reviewer for this valuable comment. We clarify the relation between skin depth, localization strength, and localization reversal as follows. The skin depth ξ characterizes the spatial decay rate of skin modes, defined as $\psi(x) \sim e^{-x/\xi}$. A smaller ξ indicates stronger localization (more confined to the boundary), while a larger ξ indicates weaker localization (more extended states).

As the system approaches the point-gap topological transition, the skin mode becomes delocalized, leading to a divergence (peak) in the skin depth ξ. After crossing the transition

point, the non-Hermitian skin effect reverses its localization direction, and the skin depth returns to a finite value, with modes now localized at the opposite boundary.Thus, the peak in skin depth serves as a clear signature of the localization reversal.

We have added this explicit explanation in the Supplementary Information: "The skin depth ξ is obtained by fitting the wavefunction profile to the form $\psi(x) \sim e^{-x/\xi}$, where a smaller ξ corresponds to stronger localization, and a larger ξ indicates weaker localization."

Reviewer comments:

The inclusion of the non-linear non-reciprocal SSH model is a valuable addition. However, the discussion of the associated figures (in particular Fig. 5) requires further clarification:

i) Do the topological edge states correspond to zero real energy? If so, it is not immediately clear how this can be identified from Fig. 5(b). A more explicit explanation in the text would be helpful.

Response:

We thank the reviewer for this insightful comment. As demonstrated in Hadad *et al.* (Phys. Rev. B **93**, 155112 (2016)), in finite-length chains, inevitable coupling between edge modes gives rise to frequency splitting of the topological states, such that their energies are generally not pinned to zero. To further confirm the emergence of topological edge states, we employ the method proposed in Zhu *et al.* (Phys. Rev. B **103**, 195414 (2021)), identifying them via their characteristic opposite localization profiles on odd and even lattice sites (under the parameter condition of $|\ln(|t_r|/|t_l|)| < 2$), a signature that is also clearly observed in our experimental measurements.

Reviewer comments:

ii) The discussion of line-gap and point-gap transitions, and their respective effects on edge states and skin modes, remains somewhat opaque. A more transparent and pedagogical explanation would significantly improve readability. The authors state that "the skin modes are localized around the right end (Fig. 5c,e)." It would be useful to clearly indicate whether both panels (c) and (e) indeed represent skin modes, and if so, how they differ from each other.

Response:

We thank the reviewer for pointing this out and we clarify that Panels (c) and (e) both represent skin modes. Panels (c) and (e) correspond to two different eigenmodes at low

intensity, both localized at the right boundary due to the nonzero point-gap winding, with Panel (e) exhibiting stronger localization than Panel (c). Panel (f) shows a skin mode at high intensity, which is localized at the left boundary after the point-gap phase transition reverses the winding direction.

Reviewer comments:

iii) The notion of a "line gap" in the present context is not sufficiently clear. Does this refer to a situation where two spectral loops are separated along the real-energy axis? A more explicit definition, possibly accompanied by a schematic illustration or more detailed discussion in the text, would greatly aid the reader.

Response: We sincerely thank the reviewer for this insightful comment. To provide a more explicit definition: a line gap exists when the entire spectrum in the complex energy plane lies strictly on one side of a reference line in the complex plane (shown as the red dashed line in Fig. R2), without intersecting it. This represents the natural non-Hermitian generalization of a conventional band gap in Hermitian systems.

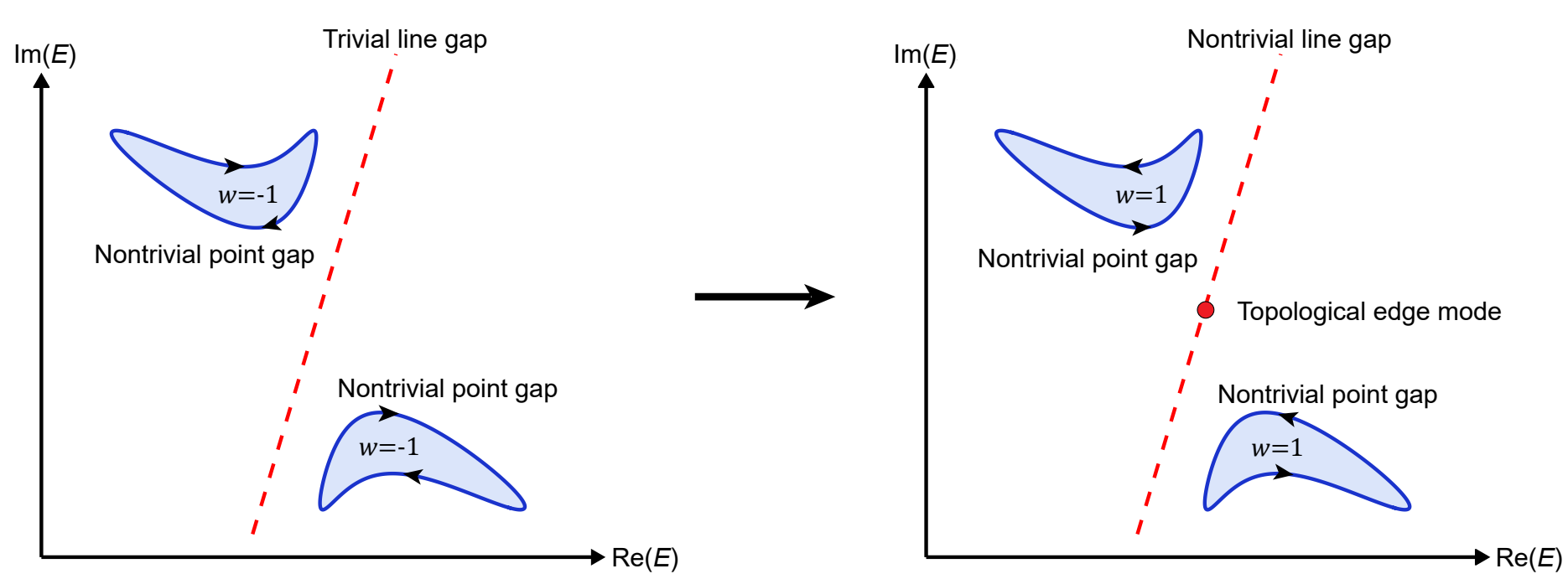

Fig. R2 Schematic illustration of point gap and line gap in the nonlinear non-reciprocal SSH model. Left: trivial line-gap phase with winding number $w = -1$. Right: nontrivial line-gap phase with $w = +1$ after the point-gap transition. Details are provided in Supplementary Note 8.

The line-gap phase transition occurs when the spectral loops cross the reference line, changing the topological character of the line gap from trivial to nontrivial, as illustrated in Fig. R2. This transition governs the emergence of topological edge states. By contrast, the point-gap phase transition corresponds to a change in the winding number of the spectral loop around a reference point in the complex plane, which is experimentally manifested as a reversal of the NHSE.

We note that the spectral signature of the line-gap transition is inconspicuous for the small lattice size (N=10) used in our experiment. Therefore, we use the emergence of topological edge states—a robust and experimentally unambiguous indicator—to identify the line-gap phase transition. Specifically, the emergence of topological edge states is verified by the opposite-localization signature on odd and even sublattices, which was experimentally observed in Fig. 5g of the main text.

We have added the following clarification to the main text: "Specifically, the point-gap topological phase transition manifests as the reversal of the NHSE, while the line-gap phase transition governs the emergence of topological edge states." In addition, a detailed discussion of the point-gap and line-gap phase transitions in the nonlinear nonreciprocal SSH model, together with the corresponding physical mechanism and experimental evidence, has been added to the Supplementary Information: "In such nonlinear nonreciprocal SSH models, both point-gap and line-gap topological phase transitions can be realized. As shown in Fig.S9, under our initial parameter settings, the intracell couplings satisfy $|t_r| > |t_l|$, so the NHSE is initially localized on the right side, corresponding to a winding number $w = -1$. Meanwhile, the condition $|t_r| \cdot |t_l| > |t_w|^2$ places the system in a trivial line-gap phase. As $|t_r|$ decreases with the increase of site intensity, the NHSE reverses, corresponding to a winding number $w = 1$, a signature of the point-gap phase transition. Furthermore, the reduction of $|t_r|$ induces the emergence of topological edge states, signaling the occurrence of the line-gap phase transition. This emergence is experimentally verified by the opposite-localization signature on odd and even sublattices, as observed in Fig. 5g of the main text. "

Reviewer comments:

Overall, while the manuscript has improved, addressing the points above would further enhance its clarity and accessibility.

Response:

We once again thank Reviewer #3 for the constructive suggestions, which have helped us further improve the manuscript. We believe the revisions described above fully address the remaining concerns.

Response Letter to Reviewers

Reviewer #3

Reviewer comments:

I have reviewed the authors' revised manuscript and their detailed response to my previous comments. I am satisfied that all of my concerns have been thoroughly addressed. In particular, I think that the findings presented in this work are novel and carry significant impact for the community. The experimental demonstration of fundamental non-Hermitian theoretical results is both timely and important. Hence, I recommend it for publications.

Response:

We thank Reviewer #3 for the recommendation.
